# Ultrasound- and NIR-Responsive Polydextran/Black TiO_2_ Nanocomposite Hydrogels for Triple-Modal Antibacterial Therapy

**DOI:** 10.1021/acsami.5c15341

**Published:** 2025-08-19

**Authors:** Tzu-Ying Wang, Yu-Ning An, Yi-Cheun Yeh

**Affiliations:** Institute of Polymer Science and Engineering, 33561 National Taiwan University, Taipei 10617, Taiwan

**Keywords:** titanium dioxide, nanocomposite hydrogel, sonodynamic therapy, photodynamic therapy, photothermal therapy

## Abstract

Titanium dioxide (TiO_2_)-containing nanocomposite hydrogels have shown great promise in biomedical applications, where TiO_2_ nanoparticles serve as sensitizers to generate reactive oxygen species (ROS) in sonodynamic therapy (SDT) and photodynamic therapy (PDT). Nevertheless, the limited bandgap of TiO_2_ nanoparticles restricts activation to ultraviolet light, and the recombination of electron–hole pairs diminishes ROS production efficiency. Here, an innovative TiO_2_-containing nanocomposite hydrogel is developed by incorporating black TiO_2_ (bTiO_2_) nanoparticles into a hydrazone-cross-linked polymeric network of polydextran aldehyde (PDA) and polydextran hydrazide (PDH). Particularly, bTiO_2_ nanoparticles are further functionalized with amino groups to form imine cross-links at the particle–polymer interface, enhancing their dispersion and also improving the mechanical properties of the network. The structures and properties of the bTiO_2_-containing PDA/PDH nanocomposite hydrogels are systematically investigated compared to the hydrogels featuring only a polymeric network and those based on white TiO_2_ nanoparticles. The bTiO_2_-containing PDA/PDH nanocomposite hydrogels exhibit increased ROS generation and exceptional photothermal properties when exposed to ultrasound and near-infrared light, significantly boosting their antibacterial efficacy through a combination of SDT, PDT, and photothermal therapy (PTT). Taken together, bTiO_2_-containing PDA/PDH nanocomposite hydrogel is a versatile therapeutic platform that offers dual external stimuli-responsiveness and dynamic features for triple-modal antibacterial applications.

## Graphical abstract

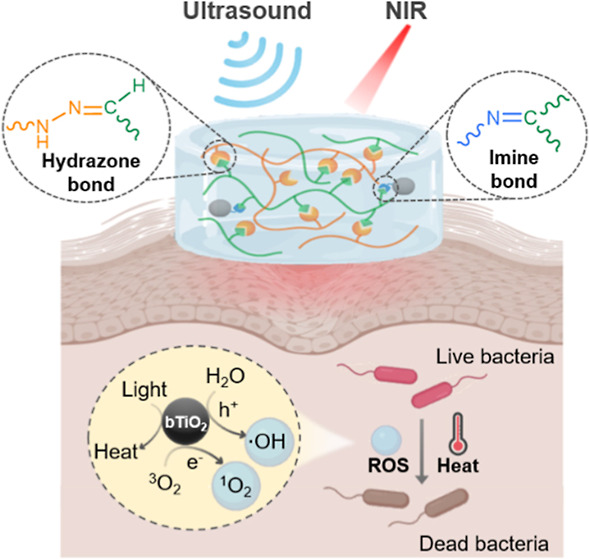

## 1 Introduction

Titanium dioxide (TiO_2_)-containing nanocomposite hydrogels have gained significant attention due to their excellent physical and chemical performance, making them highly promising for various applications (e.g., biomedical applications,^1,2^ sensors,^3,4^ environmental engineering,^5,6^ and energy storage^7,8^ ). As a cost-effective semiconductor material with photocatalytic properties, TiO_2_ nanoparticles can be activated by ultraviolet (UV) light, exciting electrons to the conduction band and leaving holes in the valence band.^9^ These high-energy charge carriers facilitate the formation of radicals, enabling the degradation of organic pollutants and promoting cell apoptosis.^10,11^ When TiO_2_ nanoparticles are incorporated into the hydrogel network, they gain improved stability, flexibility, and biocompatibility, expanding their usability across various fields. For example, Yue et al. developed a nanocomposite hydrogel integrated with TiO_2_ nanoparticles, 2,2,6,6-tetramethylpiperidine-1-oxyl (TEMPO)-oxidized chitin nanofibers, and polyacrylamide.^12^ This hydrogel utilized the photocatalytic properties of TiO_2_ nanoparticles and the recoverability of the hydrogel to enhance the degradation of organic pollutants in wastewater treatment. Gunatilake et al. reported a TiO_2_ nanotube/alginate hydrogel scaffold as a biosensor for detecting lactate and glucose in artificial sweat, enabling real-time and high-sensitive monitoring for wearable health applications.^3^

TiO_2_-containing nanocomposite hydrogels also exhibit excellent antibacterial activity as they can generate reactive oxygen species (ROS) through photocatalysis or ultrasonic activation that damages the cell membrane and DNA of bacteria. For example, Liu et al. developed a hydrogel (H@LGG/T) composed of Pluronic F127 (F127), poly-ε-lysine (EPL), oxidized hyaluronic acid (OHA), and TiO_2_-coated LiLuGeO_4_:Bi^3+^ (LGG/T).^13^ In this system, TiO_2_ nanoparticles absorbed ∼350 nm UV light emitted from the persistent luminescent LGG core to generate ROS for antibacterial effects. The hydrogel was used to treat chronic skin wounds caused by bacterial infection, and results demonstrated that it provided sustained antibacterial activity and significantly promoted wound healing. In addition to PDT, our previous work also reported the fabrication of TiO_2_-containing nanocomposite hydrogels for sonodynamic therapy (SDT).^1^ Mesoporous silica-coated titanium dioxide nanoparticles with thiolated surface functionalization (TiO_2_@MS-SH) were used as multivalent cross-linkers to react with norbornene-functionalized dextran (Nor-Dex) through ultrasound-triggered thiol–norbornene reactions and also acted as ROS generators within the hydrogel network to enable the nanocomposite hydrogels for sonodynamic antibacterial treatment.

Despite the promising antibacterial properties of TiO_2_-containing nanocomposite hydrogels, several challenges remain to complicate their practical use. The nonhomogeneous dispersion of TiO_2_ nanoparticles within the hydrogel network might lower the mechanical properties of the hydrogels and also reduce ROS production. On the other hand, TiO_2_ nanoparticles generally require either UV light^14^ or ultrasound^15^ stimulation to generate ROS due to their wide bandgap and high electron–hole recombination rate. Nevertheless, UV exposure poses potential risks to human tissues and has poor tissue penetration depth, while ultrasound-based activation suffers from poor focusing ability and the need for direct contact.

Here, we hypothesize that surface-functionalized black TiO_2_ nanoparticles could effectively address these challenges. Black TiO_2_ nanoparticles have been demonstrated to present enhanced light absorption to the near-infrared (NIR) region and decrease the electron–hole recombination rate to improve the efficiency of ROS generation compared to white TiO_2_ nanoparticles.^16–18^ Furthermore, functional groups can be added to the surface of black TiO_2_ nanoparticles to improve their interaction with the polymers in the hydrogel network, leading to better dispersion and enhanced mechanical reinforcement. We also propose that the combined activation of ultrasound and NIR light will create a synergistic antibacterial effect by utilizing sonodynamic therapy (SDT), photodynamic therapy (PDT), and photothermal therapy (PTT) to achieve effective bacterial eradication. The excellent tissue penetration of ultrasound facilitates ROS generation in deeper tissues. Beyond activating ROS production, ultrasound also induces inertial cavitation and mechanical shear forces, further disrupting bacterial membranes. For PDT and PTT, light provides superior focusing capability, allowing precise targeting of specific areas. Additionally, as a noncontact treatment modality, it offers flexibility and ease of application in clinical settings. Thus, the triple-modal therapy of SDT, PDT, and PTT presents a more attractive option for bacterial treatment.^19–21^ Nevertheless, this triple-modal therapy has only been shown in nanomaterial-based systems (e.g., TiO_2_ ^22^ and copper sulfide (CuS)^23^) for bacterial eradication, and progress is still needed in developing nanocomposite hydrogels for this combination approach to advance bacterial treatment.

In our design, we synthesized black TiO_2_ (bTiO_2_) nanoparticles with oxygen vacancies to reduce the electron–hole recombination rate for enhanced ROS generation, and also with the ability to absorb NIR light for photothermal conversion. Schiff-base cross-linking chemistry (i.e., hydrazone and imine bonds) was employed to establish polymer–polymer and polymer–nanoparticle interactions for hydrogel fabrication. These dynamic covalent bonds form rapidly under mild conditions and exhibit reversible behavior.^24^ Compared to other cross-linking strategies, Schiff-base reactions eliminate the need for potentially harmful UV irradiation and photoinitiator, as well as allow the hydrogels to have self-healing properties. Additionally, the Schiff-base cross-linking approach offers several key advantages, including the usage of simple functional groups (e.g., hydrazide, amine, and aldehyde), high reaction efficiency, and excellent biocompatibility.^25^ The bTiO_2_ nanoparticles were further modified with amino groups on the surface and incorporated into the hydrazone-cross-linked hydrogel network of polydextran aldehyde (PDA) and polydextran hydrazide (PDH) (Scheme 1). Meanwhile, these amine-modified bTiO_2_ nanoparticles can form imine bonds with the aldehyde groups on PDA, increasing the mechanical properties of the hydrogel. In this study, five types of hydrogels were fabricated, including PDA/PDH hydrogel, PDA/PDH hydrogel containing white TiO_2_ nanoparticles (PDA/PDH/WT), PDA/PDH hydrogel containing amine-modified white TiO_2_ nanoparticles (PDA/PDH/WTN), PDA/PDH hydrogel containing bTiO_2_ nanoparticles (PDA/PDH/BT), and PDA/PDH hydrogel containing amine-modified bTiO_2_ nanoparticles (PDA/PDH/BTN). Their microstructures, mechanical properties, swelling behaviors, ROS generation efficiencies, stimuli-responsiveness, antibacterial performance, and cytocompatibilities were systematically investigated.

**Scheme 1:**
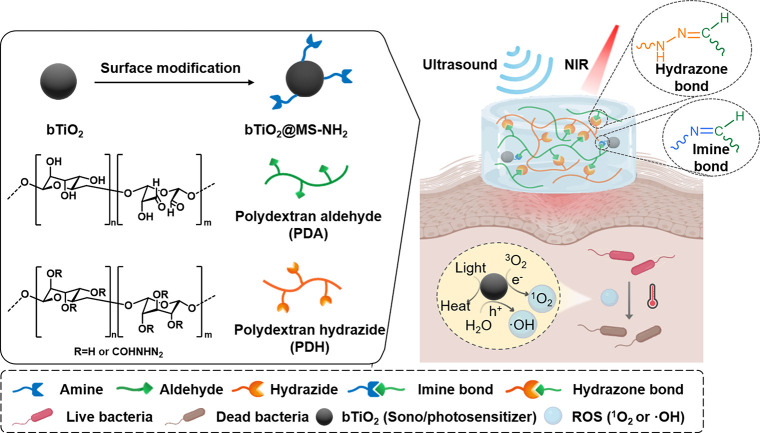
Schematic Illustrations of Material Designs

## 2 Experimental Section

### 2.1 Materials

Polydextran (*M*_w_ ca. 500 kDa and 40 kDa), (1-hexadecyl)­trimethylammonium bromide (CTAB) (98%), diethylene glycol, sodium periodate, hydrazine hydrate, and ninhydrin (99%) were purchased from Alfa Aesar. l-Ascorbic acid was purchased from Fluorochem. 1,1′-Carbonyldiimidazole was purchased from Macklin. Sodium chloride (NaCl) was purchased from SHOWA. P25 TiO_2_ nanoparticles were purchased from UniRegion Bio-Tech. Tetraethyl orthosilicate (TEOS) and titanium trichloride were purchased from Acros Organics. Ammonium hydroxide (NH_4_OH) (28–30%) was purchased from J. T. Baker. Hydrochloric acid (HCl) (∼37%) was purchased from Fisher Chemical. Phosphate-buffered saline (PBS) buffer was purchased from Bioman Scientific CO, Ltd. Imidazole, *p*-nitrosodimethylaniline (RNO), and methylene blue were purchased from Tokyo Chemical Industry CO, Ltd. Dialysis membranes with the molecular weight cutoff (MWCO) of 12–14 kDa were purchased from MFPI, USA. Bacterial strains *Staphylococcus aureus* (BCRC code: 13077) and *Escherichia coli* (BCRC code: 13055) were purchased from Bioresource Collection and Research Center in Taiwan. Soyabean Caesien Digest Medium (tryptone soy broth, TSB) and agar powder (bacteriological grade) were purchased from HIMEDIA. Dulbecco’s modified Eagle medium (DMEM), penicillin/streptomycin, fetal bovine serum (FBS), alamarBlue assay, live/dead cytotoxicity kit, and live/dead kit for bacteria were purchased from ThermoFisher. HEPES (*N*-2-hydroxyethylpiperazine-*N*′-2-ethanesulfonic acid) was purchased from Roche Applied Science. Collagen (Type I) was purchased from Sigma-Aldrich.

### 2.2 Characterization Techniques

The ^1^H nuclear magnetic resonance (NMR) spectra of polymers were characterized using a Bruker AVANCE III HD-600 MHz NMR spectrometer with samples dissolved in D_2_O. Fourier-transform infrared (FT-IR) spectra were obtained using a PerkinElmer Spectrum Two instrument. Gel permeation chromatography (GPC, Enshine SUPER CO-150) was equipped with the TSKgel G5000PW column and the Waters 2414 refractive index detector (RI). Samples were prepared by dissolving them in water, and P-82 (Pullulan) was used as the standard. The measurement was performed at a flow rate of 0.6 mL/min. The particles were characterized using electron paramagnetic resonance (EPR, Bruker EPR-plus), X-ray powder diffraction (XRD, D8-Advance), UV–vis–NIR spectrophotometry (Hitachi U-4100), and X-ray photoelectron spectroscopy (XPS, Thermo Scientific, Theta Probe). The morphology of the particles was characterized by transmission electron microscopy (TEM, Hitachi H7100, 75 kV) and scanning electron microscopy (SEM, Nova NanoSEM 230). The size distribution of the particles was determined by dynamic light scattering (DLS, ELSZ-2000 analyzer). Thermal properties of the materials were analyzed using thermogravimetric analysis (TGA, TA Instruments Q50) and differential scanning calorimetry (DSC, TA Instruments Q-20). The compression modulus and injection profile of hydrogels were characterized by a materials testing system (AGS 500N, Shimadzu). The rheological behavior was assessed using a rheometer (TA Instruments AR 2000EX). The morphology of hydrogels was characterized by SEM (Hitachi TM3000), mercury intrusion porosimeter (MIP, Auto Pore IV 9520), and microcomputed tomography (micro-CT, Skyscan-1076). Ultrasonic responsiveness experiments were performed using an ultrasound machine (US-751, ITO Ito Co., Ltd.). The setup employed a planar ultrasonic transducer with a probe diameter of 37.5 mm, beam nonuniformity ratio (BNR) of 3.0, and effective radiating area (ERA) of 5.0 cm^2^ at 1 MHz. Aquasonic 100 ultrasonic gel was applied as the coupling medium. Temperature changes were monitored with a touch-sensitive paperless recorder (VM7000 WH10, Well) and a thermal camera (FLIR-C8940). The absorbance of ROS generation probes and the fluorescence intensity of the alamarBlue reagent were measured using a microplate reader (Biotek Synergy H1). The live/dead images of cells were observed by an automated fluorescence microscope (Nikon Ti2E). The live/dead images of bacteria were observed by confocal microscopy (Leica TCS SP5).

### 2.3 Synthesis of Black TiO_2_

The synthesis of bTiO_2_ nanoparticles was performed according to the literature.^18^ Ascorbic acid (0.7 g, 4.0 mmol) was dissolved in deionized water (70 mL) and stirred at room temperature for 10 min. Titanium trichloride (TiCl_3_) (3.1 mL, 34.6 mmol) was added to the ascorbic acid solution, the pH of the mixture was adjusted to 4 by using sodium hydroxide (NaOH) (10 M), and the reaction continued at room temperature for 30 min. The mixture was then transferred to an autoclave and subjected to a reaction at 180 °C for 12 h. Finally, the resulting product was purified through centrifugation using a mixed solvent of ethanol and water in an equal volume ratio, followed by drying in a vacuum oven at 60 °C overnight to obtain the final product.

### 2.4 Synthesis of bTiO_2_@MS-NH_2_

The synthesis of bTiO_2_@MS-NH_2_ nanoparticles was performed according to the literature.^26^ Black TiO_2_ (bTiO_2_) particles (0.5 g) were suspended in ethanol (125 mL) and homogenized using ultrasonication. In a separate process, CTAB (0.4 g, 1.1 mmol) was dissolved in a mixture of ethanol (100 mL) and water (40 mL). Subsequently, NH_4_OH (25%, 12 mL, 85.7 mmol) was added to the CTAB solution. The bTiO_2_ suspension was then vigorously stirred as the CTAB solution was introduced. TEOS (0.5 mL, 2.3 mmol) solution in ethanol (25 mL) was added dropwise to the bTiO_2_ and CTAB mixture solution. The resulting mixture was stirred for an additional 20 h before being thoroughly purified through centrifugation using a mixed solvent of ethanol and water in an equal volume ratio. To remove the CTAB template, the particles were refluxed overnight in a solution of 100 mL ethanol and 12 mL HCl (37.5%). The silica-coated TiO_2_ nanoparticles were subsequently reacted with APTES (0.13 mL, 0.6 mmol) and stirred at 60 °C for 24 h. Afterward, the bTiO_2_ particles were washed three times with ethanol, centrifuged, collected, and dried under vacuum conditions.

### 2.5 Syntheses of Polydextran Aldehyde (PDA) and Polydextran Hydrazide (PDH)

PDA and PDH were synthesized according to the literature.^27,28^ Polydextran (*M*_w_ ca. 500 kDa, 10 g) was dissolved in deionized water (250 mL), sodium periodate (4.75 g, 22 mmol) was added to the polydextran solution under dark conditions, and the mixture reacted at room temperature for 24 h. Diethylene glycol (30 mL, 316 mmol) was introduced to terminate the reaction, stirring for 30 min. The PDA product was then purified via dialysis for 3 days using Cellu Sep. T4 dialysis membranes (MWCO 12–14 kDa), followed by freeze-drying to yield the dry sample.

Polydextran (*M*_w_ ca. 40 kDa, 0.5 g) was dissolved in dimethyl sulfoxide (DMSO) (20 mL) at 80 °C for 15 min to ensure complete dissolution. The solution was cooled to room temperature, and 1,1′-carbonyldiimidazole (1 g, 4.4 mmol) was then added. The resulting mixture was stirred at room temperature for 24 h. Subsequently, hydrazine hydrate (3.2 mL, 65.9 mmol) was added, and the reaction was allowed to proceed for an additional 24 h. The final PDH product was purified by dialysis using Cellu Sep. T1 dialysis membranes (MWCO 3.5 kDa) for 3 days, and the purified product was obtained by freeze-drying.

### 2.6 Preparation of Hydrogels

Three solutions were prepared for the hydrogel formation: PDA (14 wt %) and PDH (17.5 wt %) were individually dissolved in phosphate-buffered saline (PBS, pH 7.4) solution, and nanoparticles (10 wt %) were also dispersed in the PBS buffer. For PDA/PDH hydrogel formation, the PDH solution (50 μL) was first added to a trimmed syringe and homogenized. Subsequently, the PDA solution (50 μL) was added into the syringe, and the mixture was stirred for 20 s to ensure complete mixing, and then stabilized for 24 h at 25 °C. For the preparation of the PDA/PDH hydrogel containing TiO_2_ nanoparticles, the PDH solution (40 μL) and nanoparticle suspension (10 μL) were both introduced into a trimmed syringe before mixing with PDA solution (50 μL) for further stabilization at 25 °C for 24 h.

### 2.7 Rheological and Mechanical Studies of Hydrogels

The rheological properties of the hydrogels were analyzed using a rheometer configured with an 8 mm parallel plate and a cone angle of 0°. The hydrogels were molded into a cylindrical shape with a volume of 100 μL and allowed to set for 24 h before testing and positioned centrally on a Peltier plate for analysis. In the oscillatory strain sweep test, strain levels were varied from 0.1% to 1000% at 1 Hz and 25 °C. The self-healing ability of the hydrogels was examined using a cyclic oscillation time sweep test, alternating between low strain (1%) and high strain (500%) at 1 Hz and 25 °C, with each cycle lasting 1 min. Shear-thinning behavior was demonstrated by conducting a step flow test, where the shear rate was increased incrementally from 0.1 to 100 s^–1^.

For the compression tests, the hydrogels (100 μL) were similarly molded in cylindrical shape (diameter = ∼4.5 mm, height = ∼4.8 mm) and left to stabilize overnight prior to measurement. The compressive modulus was determined using a mechanical testing system, applying compression with a 10 N load cell at a rate of 1 mm min^–1^. The compressive modulus was calculated by fitting the stress–strain data within the 10–20% strain range.

### 2.8 Microstructural Analysis of Hydrogels

The lyophilized hydrogels were prepared for microstructural analysis by being frozen at −80 °C for 12 h and lyophilized using a freeze-dryer (0.1 Pa) for 3 days. The morphology of the lyophilized hydrogels was subsequently analyzed using a scanning electron microscope (SEM). Pore size analysis was performed on the SEM images using ImageJ software. The average pore size for each hydrogel sample was calculated based on measurements taken from 30 distinct pores, with 30 individual measurements performed for each pore, and the results were averaged. Furthermore, the porosity and pore size distribution of the lyophilized hydrogels were quantified using a mercury intrusion porosimeter (MIP) and microcomputed tomography (micro-CT).

### 2.9 Swelling Ratio and Water Content of Hydrogels

The hydrogels (100 μL) were lyophilized and subsequently immersed in PBS (1 mL) at 37 °C for 24 h. After the equilibration period, the swollen hydrogels were carefully removed, and any excess surface moisture was gently blotted using filter paper. The hydrogels were then weighed to determine the equilibrium swelling ratio. The equilibrium swelling ratio was calculated using the following equation

1 $$swelling\;ratio=\frac{W_{s}}{W_{d}}$$

The equilibrium water content is calculated by the following equation

2 $$water\;content=\frac{W_{s}-W_{d}}{W_{s}}\times 100\%$$

where *W*_s_ is the weight of hydrogels at the swelling state and *W*_d_ represents the weight of dried hydrogels.

### 2.10 Degradation Rate of Hydrogels

The weight of the lyophilized hydrogels was recorded as the initial dry weight (*W*_0_) (∼13 mg). The samples were then immersed in 1 mL of PBS and incubated at 37 °C on a shaker set to 100 rpm (r.p.m.). At predetermined time intervals, the hydrogels were removed, lyophilized once again, and the final dry weight (*W*_1_) was measured. The degradation ratio was calculated using the following equation

3 $$weight\;remaining\;\left(\%\right)=\frac{W_{1}}{W_{0}}\times 100\%$$

### 2.11 Singlet Oxygen (^1^O_2_) Detection

Hydrogels (100 μL) immersed in DI water (1 mL) were mixed with *p*-nitrosodimethylaniline (RNO) (0.05 mM, 2 μL) and imidazole (0.05 mM, 10 μL), then exposed to either ultrasound (1 MHz, 1 W cm^–2^, and 50% duty cycle) or an NIR laser (808 nm, 1 W cm^–2^). For singlet oxygen detection, the decay of the RNO absorption peak at 440 nm was measured at 1, 3, and 5 min during the exposure period.

### 2.12 Hydroxyl Radical (^•^OH) Detection

The generation of hydroxyl radicals (^•^OH) can be detected using methylene blue (MB) as a probe. Hydrogels were immersed in a MB solution (1 mL) and exposed to either ultrasound (1 MHz, 1 W cm^–2^, and 50% duty cycle) or NIR (808 nm, 1 W cm^–2^) irradiation. The changes in absorbance at 665 nm were recorded to monitor the degradation of MB, indicating the formation of hydroxyl radicals.

### 2.13 Thermal Behavior of Hydrogels under Irradiation

The hydrogels were immersed in DI water and irradiated with an 808 nm laser at various power densities for 5 min. The temperature changes of the hydrogels were monitored with a touch-sensitive paperless recorder and a thermal camera (FLIR-C8940). The heating–cooling curve of the hydrogels was obtained at a power density of 1.0 W cm^–2^.

### 2.14 *In Vitro* Antibacterial Test of Hydrogels

A tryptone soy broth (TSB) solid medium was prepared by dissolving 3% (w/v) TSB and 1% (w/v) agar powder into deionized water, which was subsequently utilized in the spotting assay. For the cultivation and maintenance of *S. aureus* and *E. coli*, bacterial stocks were thawed and streaked onto TSB agar plates, followed by incubation at 37 °C overnight. After incubation, the resulting colonies were selected and transferred into a PBS buffer.

The study assessed the impact of ultrasound and NIR on *S. aureus* (150 μL, O.D._600_ = 0.1) and *E. coli* (150 μL, O.D._600_ = 0.05). The spread plate assay was used to assess the effects of hydrogels on bacterial samples under ultrasound and NIR irradiation. Samples were diluted in a sterilized PBS solution. For colony growth observation, 100 μL of each diluted sample was placed on TSB agar plates and incubated at 37 °C for 14 h.

The morphology of bacteria was observed by SEM. The treatment details were the same as those of the spread plate assay. After treatment, the hydrogels were removed from the bacterial solutions, and the bacterial samples were fixed in glutaraldehyde (2.5 wt %) at 4 °C for 1 h. The fixed samples were dehydrated continuously in ethanol rows (20%, 40%, 60%, 80%, and 100%), each for 15 min. After drying in a critical point dryer for 3 h, the samples were gold-coated for enhanced SEM visualization.

For live/dead staining of bacteria, the details of the treatment were consistent with the spread plate assay. The live/dead kit combined equal volumes of SYTO9 and PI dyes. The bacterial solutions (1 mL) were simultaneously stained for 15 min in the dark with the mixture dye (3 μL). Finally, the images of bacteria were observed by confocal microscopy.

### 2.15 *Ex Vivo* Antibacterial Test

Porcine skin was stored at −20 °C. The skin was thawed, and subcutaneous fat was removed using a scalpel before the experiment. The prepared skin was placed in a Petri dish lined with paper towels saturated with ultrapure sterile water to maintain hydration. The skin surface was subsequently cleaned with 75% ethanol.

Standardized circular wounds were created using a biopsy punch by excising the epidermis while preserving the underlying dermis, generating wounds approximately 0.5–1 mm in depth and 9 mm in diameter. The wound area within the biopsy site was rinsed twice with 200 μL of sterile ultrapure water to remove residual debris. Then, 100 μL of bacterial suspension (*S. aureus* or *E. coli*) was applied to each wound to establish infection. The Petri dish was covered with a lid and incubated at 37 °C for 2 h. Following 2 h infection period, hydrogels (100 μL) or sterile ultrapure water (control group) were applied to the infected wound. For the treatment group, ultrasound and NIR irradiation were administered at an intensity of 1 W cm^–2^ for 5 min. Post-treatment, the samples were further incubated at 37 °C for 30 min.

For bacterial recovery, PBS (100 μL) was introduced into the wound, and the resulting suspension was then collected into a sterile tube. This recovery process was repeated twice, and samples were diluted in a sterile PBS solution. The diluted suspensions were subsequently plated on agar plates and incubated overnight at 37 °C for 18 h. Bacterial colony counts were determined, normalized to the control group, and analyzed to evaluate the antimicrobial efficacy of the treatment.

### 2.16 *In Vitro* Cytocompatibility of Hydrogels

Mouse embryonic fibroblasts (MEFs) were isolated from C57BL/6N mouse embryos. MEFs were cultured under standard conditions in a humidified incubator maintained at 37 °C with 5% CO_2_. The culture medium used was DMEM supplemented with 10% fetal bovine serum (FBS), 1% penicillin/streptomycin, and 1% HEPES.

The cytocompatibility of hydrogels was evaluated according to the ISO 10993 guideline.^29^ The hydrogel extracts were prepared following ISO 10993-12 procedures. Briefly, hydrogels were sterilized under UV irradiation for 1 h and then incubated in serum-containing DMEM (10% FBS and 1% antibiotic) at a hydrogel-to-medium volume ratio of 1:10 for 24 h at 37 °C, 5% CO_2_, and 90% humidity in a sterile incubator. All extracts were used within 24 h after preparation.

For cytocompatibility testing, MEFs were seeded in 96-well plates at a density of 1.5 × 10^4^ cells mL^–1^ (200 μL per well) and incubated with the hydrogel extracts under standard culture conditions (37 °C, 5% CO_2_). In addition, for direct coculture experiments, cells were directly seeded onto hydrogels (30 μL) and cocultured, with an additional 20 μL of collagen solution (2 mg mL^–1^) added to promote cell adhesion. After 24 h of incubation, cell viability was assessed using the alamarBlue assay by adding 200 μL of alamarBlue solution (10% in serum-containing DMEM) to each well and incubating for an additional 4 h.

To quantify cell viability, the fluorescence intensity of the alamarBlue reagent was measured using a microplate reader with excitation at 560 nm and emission at 590 nm. The background fluorescence of alamarBlue was subtracted before further calculations. The fluorescence intensity directly correlates with the metabolic activity of the cells, providing a reliable measure of the cytocompatibility of the hydrogels.

Cell viability was also assessed using a live/dead staining assay to differentiate between live and dead cells. For the assay, MEFs (3 × 10^4^ cells mL^–1^, 200 μL) were seeded into a 96-well plate and incubated with hydrogel extract solutions. To identify live and dead cells, the cells were stained with calcium-AM (2 μM, green fluorescence) and ethidium homodimer-1 (4 μM, red fluorescence), respectively. After a 40 min incubation, the samples were washed twice with PBS solution and subsequently analyzed using a fluorescence microscope.

### 2.17 Statistical Analysis

All experiments were performed in triplicate unless otherwise specified. Statistical analyses were conducted using SPSS statistical software. The significance of the data was assessed through one-way analysis of variance (ANOVA), followed by posthoc tests for pairwise comparisons of group differences. Statistical significance was determined at a threshold of *p* < 0.05. Significant results were indicated: *p < 0.05, **p < 0.01, ***p < 0.001, and ns for no significant difference.

## 3 Results and Discussion

### 3.1 Syntheses and Characterizations of PDA and PDH Polymers

Polydextran aldehyde (PDA) was synthesized through the oxidation of polydextran using sodium periodate.^30^ In the ^1^H nuclear magnetic resonance (NMR) spectrum of polydextran, multiple peaks in the region of 3.2 to 3.9 ppm were assigned to the protons of the glucose units (Figure S1). After oxidation, peaks between 5.0 and 5.7 ppm in the ^1^H NMR spectrum of PDA belonged to the protons of the hemiacetal structures, showing that aldehyde groups were formed within the polydextran chain. The degree of oxidation and the quantification of the aldehyde functionality were determined by reacting PDA with hydroxylamine hydrochloride,^31^ revealing an oxidation level and the concentration of aldehydes in PDA was 26.5% and 2.8 mmol g^–1^, respectively. The molecular weight of PDA was determined by gel permeation chromatography (GPC), showing the average molecular weight (*M*_w_) of PDA was 233 kDa with polydispersity index (PDI) = 1.16.

Carbazate groups were introduced to the polydextran structure through a two-step process.^28^ 1,1′-Carbonyldiimidazole (CDI) was first reacted with polydextran to form the imidazole carbonate intermediates (alkyl carbamate) in polydextran, and then hydrazine was added to displace the imidazole groups and yield carbazate groups (–O–C­(O)–NHNH_2_) in polydextran, forming polydextran hydrazide (PDH). In the ^1^H NMR spectrum of PDH, the peak at 5.28 ppm was attributed to the protons of the –NH– groups, and multiple peaks in the region of 3.2 to 3.9 ppm were assigned to the protons of the glucose units (Figure S2a). In the Fourier-transform infrared (FT-IR) spectra of PDH, a distinct C=O stretching vibration at 1710 cm^–1^ also indicated the successful introduction of the carbazate group (Figure S2b). These results confirmed the successful synthesis of PDH. The amount of amines in PDH was 5.8 × 10^–3^ mmol g^–1^ determined through the ninhydrin test. The *M*_w_ of PDH was 56 kDa with PDI = 1.16.

### 3.2 Syntheses and Characterizations of Black TiO_2_ Nanoparticles

Black TiO_2_ (bTiO_2_) nanoparticles were synthesized through a hydrothermal process by using Ti­(III)-salt as the precursor and l-ascorbic acid as a reductant and structure-directing agent.^18^ The presence of oxygen vacancies in bTiO_2_ nanoparticles was confirmed through electron paramagnetic resonance (EPR) analysis, showing a distinct signal at a g-value of 2.003, indicative of oxygen vacancies with trapped electrons rather than Ti^3+^ ions (*g* = 1.96–1.99) (Figure S3a). X-ray diffraction (XRD) analysis further demonstrated that bTiO_2_ nanoparticles exhibited reduced crystallinity due to the formation of an oxygen-deficient amorphous layer while retaining the anatase crystal structure, as evidenced by characteristic diffraction peaks at 25.4° (101), 48.3° (200), and 55.2° (211) (Figure S3b). The ultraviolet–visible–near-infrared (UV–vis–NIR) absorption spectra of both bTiO_2_ and white TiO_2_ (wTiO_2_) nanoparticles confirmed significant absorption below 400 nm, corresponding to anatase intrinsic band gap absorption of TiO_2_ nanoparticles (Figure S3c). Notably, bTiO_2_ nanoparticles showed an extended absorption range from the visible to the NIR region, consistent with their observed black color.

The TiO_2_ nanoparticles were coated with mesoporous silica shells via the sol–gel method using tetraethyl orthosilicate (TEOS) and cetyltrimethylammonium bromide (CTAB) as a silica precursor and template, respectively, forming TiO_2_@MS.^26^ The TiO_2_@MS nanoparticles were further functionalized with amino groups through a reaction with 3-aminopropyltriethoxysilane (APTES), forming wTiO_2_@MS-NH_2_ and bTiO_2_@MS-NH_2_ nanoparticles. Scanning electron microscopy (SEM), transmission electron microscopy (TEM), and dynamic light scattering (DLS) analysis revealed reduced aggregation of TiO_2_ nanoparticles following the coating of amine-modified silica shells (Figures 1a–c and S4).

**Figure 1:**
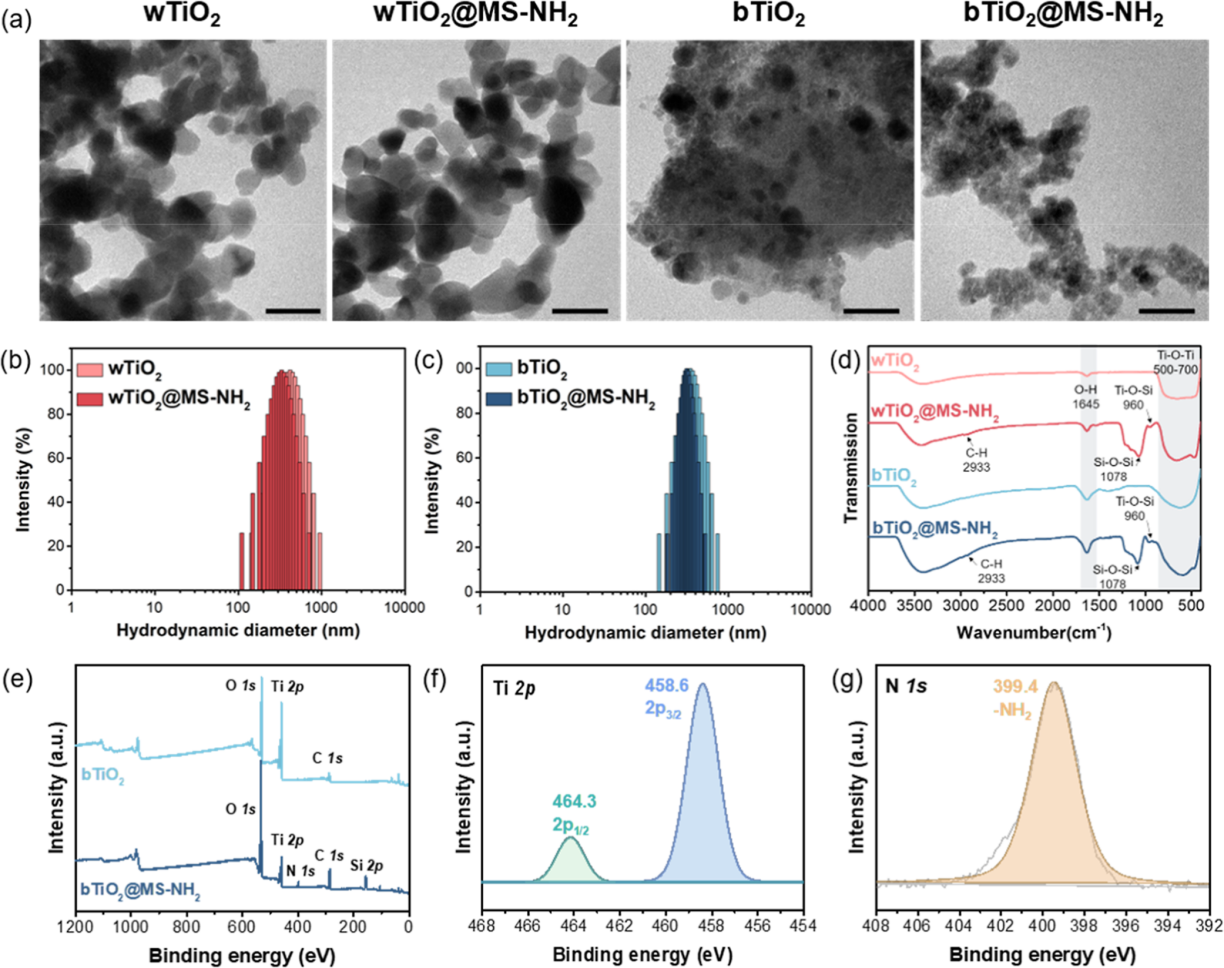
(a) TEM images (scale bar: 50 nm), (b,c) hydrodynamic diameter distributions, and (d) FT-IR spectra of nanoparticles. (e) XPS survey spectra of bTiO_2_ and bTiO_2_@MS-NH_2_ nanoparticles. High-resolution XPS spectra for (f) Ti 2p and (g) N 1s of bTiO_2_@MS-NH_2_ nanoparticle.

The TEM–EDS (transmission electron microscopy with energy dispersive X-ray spectroscopy) elemental mapping results of wTiO_2_@MS-NH_2_ and bTiO_2_@MS-NH_2_ showed that Ti and O signals were uniformly distributed (Figure S5a,b), confirming the presence of TiO_2_. The signals of Si and N elements were also clearly detected and distributed across the observed regions, suggesting the successful incorporation of mesoporous silica shell and amine functional groups. The EDS spectra further confirmed the elemental composition of both samples, revealing dominant peaks corresponding to Ti and O, consistent with TiO_2_ (Figure S5c,d). The Si signals indicated the formation of a silica shell surrounding the TiO_2_ core. In contrast, the N signal was not prominent in the EDS spectra, which can be attributed to the known limitations of EDS in accurately detecting and quantifying light elements such as nitrogen, boron, and beryllium.^32^ However, nitrogen was clearly observed in the elemental mapping results. This is likely because EDS mapping allows for signal accumulation over a user-defined acquisition time, and extending the dwell time enhances the detectability of weak signals from light elements. To validate the presence of nitrogen and confirm the successful amine functionalization, XPS was further employed, which clearly demonstrated the existence of the amine-functionalized surface.

We also evaluated the stability of the nanoparticles in both powder and solution forms over a period of 7 days. In solution, notable precipitation was observed for wTiO_2_ and bTiO_2_ nanoparticles as early as day one, whereas wTiO_2_@MS-NH_2_ and bTiO_2_@MS-NH_2_ nanoparticles exhibited visible precipitation starting on day three (Figure S6). To further assess stability, we measured the hydrodynamic diameters of the nanoparticles at various time points. Prior to each measurement, the samples were sonicated to ensure dispersion. The results demonstrated that the hydrodynamic sizes remained consistent throughout the 7 day period, regardless of whether the nanoparticles were stored in a dry or suspended state (Figure S7 and Table S1).

Previous studies have reported that bTiO_2_ nanoparticles are inherently prone to aggregation.^18^ To address this issue, we employed a surface functionalization strategy aimed at reducing nanoparticle aggregation. However, it is important to note that excessive amine or silica modifications might negatively affect ROS release, as such modifications might alter the surface chemistry of the nanoparticles to inhibit ROS generation or affect their release behavior. To balance effective surface modification with optimal ROS activity, we followed modification ratios recommended in relevant literature during the synthesis process. This approach was intended to achieve sufficient surface functionalization while preserving the desired ROS release characteristics. Consequently, the comparison of nanoparticle aggregation levels in this study was presented in a relative context.

FT-IR spectroscopy confirmed the successful encapsulation of TiO_2_ nanoparticles with silica shells, as evidenced by Si–O–Si and Ti–O–Si bonds at 1078 and 960 cm^–1^, respectively (Figure 1d). Additionally, the appearance of the C–H stretching vibration at 2933 cm^–1^, combined with these Si–O–Si and Ti–O–Si peaks, provided strong evidence of the successful grafting of NH_2_ groups onto the surfaces of wTiO_2_@MS-NH_2_ and bTiO_2_@MS-NH_2_ nanoparticles. The functionalization is likely stabilized by the silica layer, which enhances the attachment and retention of the amino groups on the nanoparticle surfaces. Furthermore, ninhydrin assays indicated amino group contents of 0.011 and 0.010 mmol mg^–1^ for wTiO_2_@MS-NH_2_ and bTiO_2_@MS-NH_2_ nanoparticles, respectively.

The element-valence state and chemical composition of bTiO_2_ and bTiO_2_@MS-NH_2_ nanoparticles were investigated using X-ray photoelectron spectroscopy (XPS) analysis. The full survey XPS spectrum of bTiO_2_@MS-NH_2_ nanoparticle revealed the presence of Ti, O, C, N, and Si elements (Figure 1e). The Ti 2p spectrum showed only Ti 2p_1/2_ (464.3 eV) and Ti 2p_3/2_ (458.6 eV) peaks, with no Ti^3+^ peaks (463.3 eV), which further confirmed that precursor TiCl_3_ did not exist in bTiO_2_@MS-NH_2_ nanoparticles (Figure 1f). The characteristic Ti–O–Si bond was observed at 101.8 and 530.9 eV in the Si 2p and O 1s spectra, respectively (Figure S8a,b), confirming the core–shell structure of bTiO_2_@MS-NH_2_ nanoparticles. The N element state was analyzed, and the typical NH_2_ and C–N binding energies were observed at 399.4 and 286.4 eV in the N 1s and C 1s spectra, respectively (Figures 1g and S8c). These results confirmed that the amino groups have been introduced to the surface of the bTiO_2_@MS-NH_2_ nanoparticles.

The photothermal properties of nanoparticles were investigated by infrared thermal imaging under NIR irradiation (808 nm, 1 W cm^–2^) (Figure S9a). The results showed that the temperatures of bTiO_2_ and bTiO_2_@MS-NH_2_ nanoparticles were increased to ∼125 °C, while the temperatures of wTiO_2_ and wTiO_2_@MS-NH_2_ nanoparticles did not change. Additionally, a thermocouple thermometer was used to verify the temperature increase of the bTiO_2_ and bTiO_2_@MS-NH_2_ nanoparticles during NIR irradiation (Figure S9b). Given the larger quantity of nanoparticle powder required for thermocouple-based temperature measurements, the temperatures of bTiO_2_ and bTiO_2_@MS-NH_2_ nanoparticles were observed to increase up to 160–170 °C, as confirmed by both thermal imaging and thermocouple readings. These results demonstrated that bTiO_2_ and bTiO_2_@MS-NH_2_ nanoparticles were capable of reaching temperatures exceeding 100 °C under NIR irradiation. The bTiO_2_ nanoparticles exhibited excellent photothermal conversion efficiency due to their strong absorption in the NIR region (Figure S3c), and the surface modification did not affect their photothermal conversion performance.

A clinically relevant, medical-grade ultrasound device was used for all sonication procedures, potentially reducing the translational gap between experimental validation and future clinical application of the nanocomposite hydrogels. The ultrasound settings in this work (i.e., 1 MHz, 1 W cm^–2^, 50% duty cycle) were similar to those found in other SDT studies that also used TiO_2_ nanoparticles as sonosensitizers,^33,34^ allowing the comparison between these SDT research works.

The sonodynamic and photodynamic properties of nanoparticles were investigated using *p*-nitrosodimethylaniline (RNO)/imidazole as a probe for singlet oxygen (^1^O_2_) (Figure S10a).^35^ In general, imidazole reacts with ^1^O_2_ to form a peroxide intermediate, which subsequently reacts with RNO and leads to a noticeable decrease in the absorbance of RNO at 440 nm. When TiO_2_ suspensions were under ultrasound (US) irradiation (1 MHz, 1 W cm^–2^, and 50% duty cycle), the absorbance of the RNO probe immersed in the bTiO_2_ and bTiO_2_@MS-NH_2_ solutions was relatively lower compared to that in the wTiO_2_ and wTiO_2_@MS-NH_2_ solutions, indicating bTiO_2_ and bTiO_2_@MS-NH_2_ nanoparticles produced a higher amount of ^1^O_2_ compared to the others. Notably, the surface modification of bTiO_2_ nanoparticles did not affect their ability to generate ^1^O_2_ (Figure S10b). Additionally, the NIR-absorbing bTiO_2_ nanoparticles can produce ^1^O_2_ upon irradiating 808 nm NIR light (Figure S10c). In the cotreatment of US and NIR exposures, a significant drop in absorbance at 440 nm was observed, demonstrating the rapid production of ^1^O_2_ under the combined effects of US and NIR on the bTiO_2_ nanoparticles (Figure S10d).

In addition to ^1^O_2_ detection, hydroxyl radical (^•^OH) was also evaluated to assess the ROS generation capabilities of the nanoparticles. The detection of ^•^OH was performed using methylene blue (MB) as a probe (Figure S10e).^36,37^ When ^•^OH reacts with MB, a degradation process occurs and leads to a decrease in the absorbance of MB at 665 nm. The results showed that the bTiO_2_ and bTiO_2_@MS-NH_2_ nanoparticles demonstrated the most pronounced reduction in the absorbance of MB under US and/or NIR exposures compared to wTiO_2_ and wTiO_2_@MS-NH_2_ nanoparticles, indicating their greater efficiency in generating ^•^OH (Figure S10f–h). These results revealed the high ^1^O_2_ and ^•^OH generation efficiency of bTiO_2_ nanoparticles when exposed to US and NIR.

The mechanisms of ROS generation by black TiO_2_ nanoparticles under ultrasound and NIR light were further illustrated.^38^ Under ultrasound irradiation, the inertial cavitation effect induced by ultrasound in liquid media leads microbubbles to grow and then collapse rapidly. When these bubbles implode, they release enormous energy, producing transient temperatures of up to 10,000 K and pressures near 81 MPa, which directly cleave water molecules into hydroxyl radicals (^•^OH) and hydrogen atoms (H^•^). Concurrently, the inertial cavitation process is accompanied by sonoluminescence, which can activate the sonosensitizer bTiO_2_ nanoparticles whose energy levels match the emitted photons to generate electron–hole pairs and enhance the production of ROS. On the other hand, for PDT, bTiO_2_ nanoparticles act as photosensitizers by absorbing photons, which promote electrons from the valence band to the conduction band, leaving holes in the valence band.^39^ The excited electrons interact with surrounding oxygen molecules to produce singlet oxygen (^1^O_2_), while the holes oxidize water molecules and generate hydroxyl radicals (^•^OH).

### 3.3 Preparations and Characterizations of Hydrogels

Hydrogel formulations with various polymer and nanoparticle amounts were adjusted in different proportions, and the optimal mechanical properties were determined through compression and rheological tests. The polymeric network of hydrogels was established through hydrazone bonds formed between the aldehyde groups of PDA and the hydrazide groups of PDH. The results revealed that the combination of 7 wt % PDA and 7 wt % PDH exhibited the best mechanical performance among the tests, showing a compression modulus of ∼60 kPa and a storage modulus of ∼3970 Pa (Figure S11a–c). On the other hand, amine-functionalized bTiO_2_@MS-NH_2_ nanoparticles were further introduced into the hydrogel network, creating imine bonds between the amines of bTiO_2_@MS-NH_2_ nanoparticles and the aldehydes of PDA. PDA/PDH nanocomposite hydrogel (7 wt %/7 wt %) containing bTiO_2_@MS-NH_2_ (1 wt %) presented the highest mechanical strength among the tested groups, showing compression modulus of ∼83 kPa and storage modulus of ∼4600 Pa (Figure S11d–f). These results revealed that the introduction of a suitable amount of bTiO_2_@MS-NH_2_ nanoparticles enhanced the mechanical properties of the hydrogels due to the imine bonds between the nanoparticles and the polymers, while excessive content of nanoparticles generated steric hindrance that disrupted the hydrazone cross-link formation between polymers to reduce the mechanical properties of the hydrogels. Thus, 7 wt % PDA, 7 wt % PDH, and 1 wt % nanoparticles were finalized as the nanocomposite hydrogel formula for this study.

Five types of hydrogels were synthesized by combining PDA and PDH in the absence or presence of nanoparticles (i.e., wTiO_2_, wTiO_2_@MS-NH_2_, bTiO_2_, or bTiO_2_@MS-NH_2_), resulting in the formation of PDA/PDH, PDA/PDH/wTiO_2_ (PDA/PDH/WT), PDA/PDH/wTiO_2_@MS-NH_2_ (PDA/PDH/WTN), PDA/PDH/bTiO_2_ (PDA/PDH/BT), and PDA/PDH/bTiO_2_@MS-NH_2_ (PDA/PDH/BTN) hydrogels. The FT-IR spectra of the hydrogels showed that the peak at 1720 cm^–1^ corresponded to the carbonyl (C=O) bonds presented in PDH was shifted to 1728 cm^–1^, indicating the formation of new hydrazone bonds within the hydrogel (Figure S12). Additionally, the peak at 1640 cm^–1^ was a combination of the C=C bonds on the PDA aromatic rings, the –CO–NH groups in PDH, and the C=N bonds in the hydrogel.^40–42^

### 3.4 Microstructures and Properties of Hydrogels

The microstructures of hydrogels were characterized using SEM, microcomputed tomography (micro-CT), and mercury intrusion porosimetry (MIP), where the hydrogels were lyophilized before measurement. SEM is a widely used tool for qualitative analysis of hydrogel microstructures. On the other hand, micro-CT and MIP provide quantitative insights into pore architecture. Micro-CT enables noninvasive 3D imaging of pore size and porosity distribution,^43^ MIP measures pore size by tracking mercury intrusion under increasing pressure.^44^

SEM was used to measure the cross sections of the lyophilized hydrogels, and was mainly used to reveal the porous hydrogel structures. The pore sizes of PDA/PDH, PDA/PDH/WT, PDA/PDH/WTN, PDA/PDH/BT, and PDA/PDH/BTN lyophilized hydrogels determined through SEM analysis were ∼39, 42, 33, 42, and 27 μm, respectively (Figure 2a,c). Micro-CT images provided additional visualization of the pore size distribution of the 3D lyophilized hydrogel structures, with color images showing blue for larger pores, green for medium-sized pores, and red for smaller pores (Figure 2b). The pore sizes of PDA/PDH, PDA/PDH/WT, PDA/PDH/WTN, PDA/PDH/BT, and PDA/PDH/BTN lyophilized hydrogels were ∼118, 129, 106, 135, and 92 μm, respectively (Table S2). The total porosity of these lyophilized hydrogels was above 75%, and the open porosity was greater than the closed porosity, indicating that the hydrogels possessed a three-dimensional interconnected network. The MIP results showed that the median pore diameters of PDA/PDH, PDA/PDH/WT, PDA/PDH/WTN, PDA/PDH/BT, and PDA/PDH/BTN lyophilized hydrogels were ∼35, 44, 33, 46, and 28 μm, respectively (Figure 2d,e and Table S3). The total pore area and the porosity of the lyophilized hydrogels were all above 0.7 m^2^ g^–1^ and 78.7%, respectively. The observed trends of the porous structures of lyophilized hydrogels determined by SEM, micro-CT, and MIP were consistent, supporting the reliability of these techniques for characterizing hydrogel porosity. Furthermore, in the MIP analysis, lyophilized hydrogels with smaller median pore diameters typically contain more uniformly distributed small pores, contributing to a significant increase in the total intrusion volume. Thus, the PDA/PDH/BTN lyophilized hydrogel with the smallest median pore diameter (∼28 μm) and the highest total intrusion volume (∼4.0 mL g^–1^) among the five lyophilized hydrogels presented a more refined and densely packed pore structure that led to the enhanced total intrusion volume. Overall, bTiO_2_@MS-NH_2_ nanoparticles acted as secondary cross-linkers in the polymeric networks to construct densely cross-linked structures by introducing imine bonds in the PDA/PDH/BTN hydrogel.

**Figure 2:**
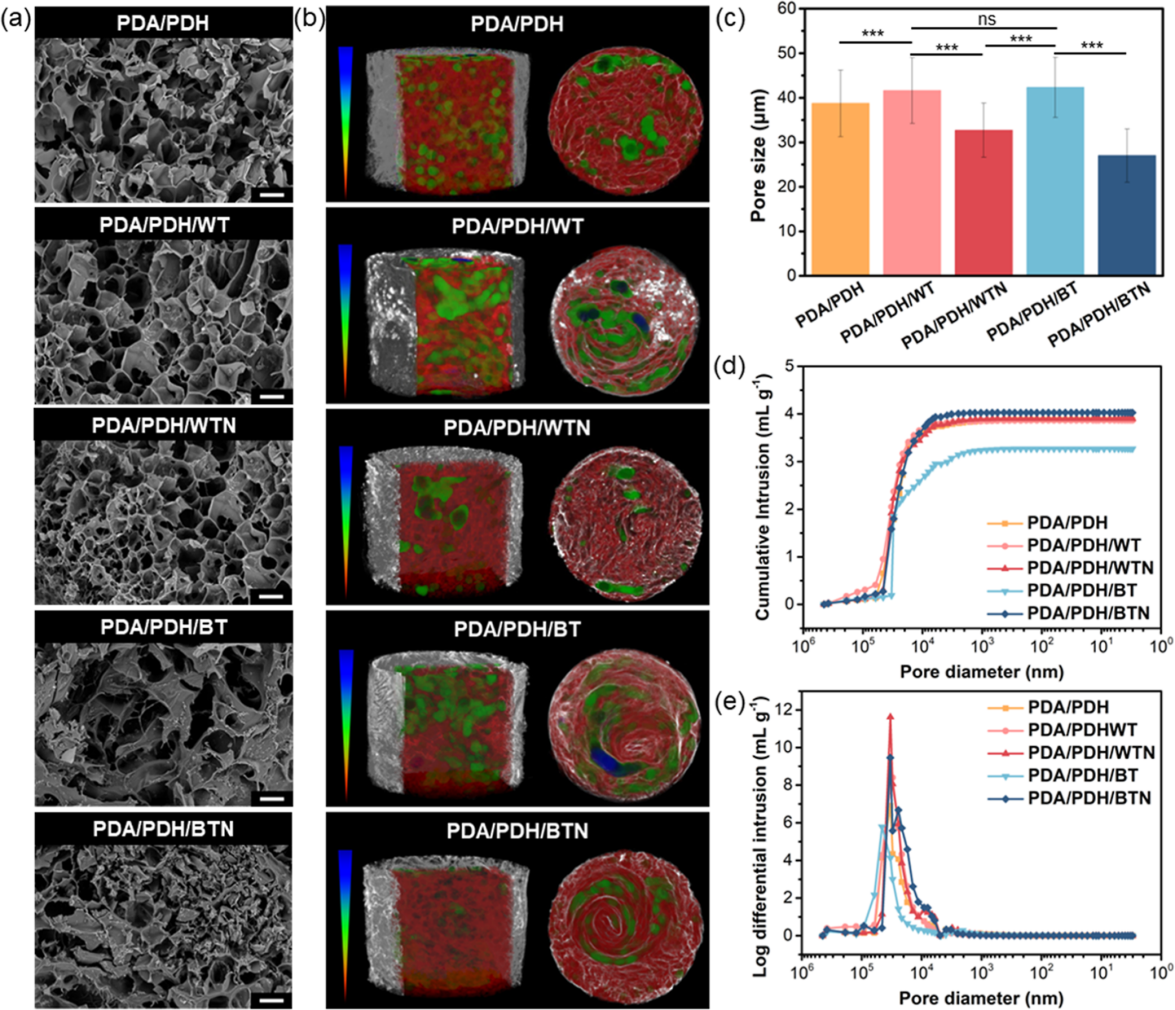
(a) SEM images (scale bar: 50 μm), (b) micro-CT images, and (c) the average pore sizes of hydrogels determined by SEM analysis. Pore size analysis was based on 900 measurements (*n* = 900), obtained by performing 30 repeated measurements on each of 30 randomly selected pores from SEM images. (d) Intrusion-desorption curves and (e) differential intrusion curves by MIP tests. Significant results were indicated: ***p < 0.001 and ns for no significant difference.

The distribution of TiO_2_ nanoparticles within the polymeric network can be characterized by analyzing the elemental distribution in lyophilized hydrogels using SEM–EDS (scanning electron microscopy with energy dispersive X-ray spectroscopy) mapping. Elemental analysis of the TiO_2_-containing hydrogels revealed the presence of carbon (C), nitrogen (N), oxygen (O), and titanium (Ti), with the Ti signal specifically attributed to the TiO_2_ NPs (Figure S13). The uniform dispersion of Ti observed in the EDS images confirmed that the TiO_2_ nanoparticles were homogeneously distributed throughout the cross-linked polymeric matrix.

The mechanical properties of the hydrogels were assessed using compression tests. The compression moduli of the PDA/PDH, PDA/PDH/WT, PDA/PDH/WTN, PDA/PDH/BT, and PDA/PDH/BTN hydrogels were ∼57, 19, 91, 20, and 83 kPa, respectively (Figure 3a,b). The rheological properties of the hydrogels were investigated using an oscillation time sweep, showing the storage modulus (*G*′) values of PDA/PDH, PDA/PDH/WT, PDA/PDH/WTN, PDA/PDH/BT, and PDA/PDH/BTN hydrogels were ∼3900, 3400, 4700, 3500, and 4500 Pa, respectively (Figure 3c). Oscillation strain sweeps further revealed that the hydrogels reached a flow point (where *G*′ = *G*″) at higher strain values, transitioning to liquid-like behavior, indicating energy dissipation and microstructure disruption. The flow points of PDA/PDH, PDA/PDH/WT, PDA/PDH/WTN, PDA/PDH/BT, and PDA/PDH/BTN hydrogels were ∼58%, 59%, 103%, 70%, and 93%, respectively (Figure 3d and Table S4).

**Figure 3:**
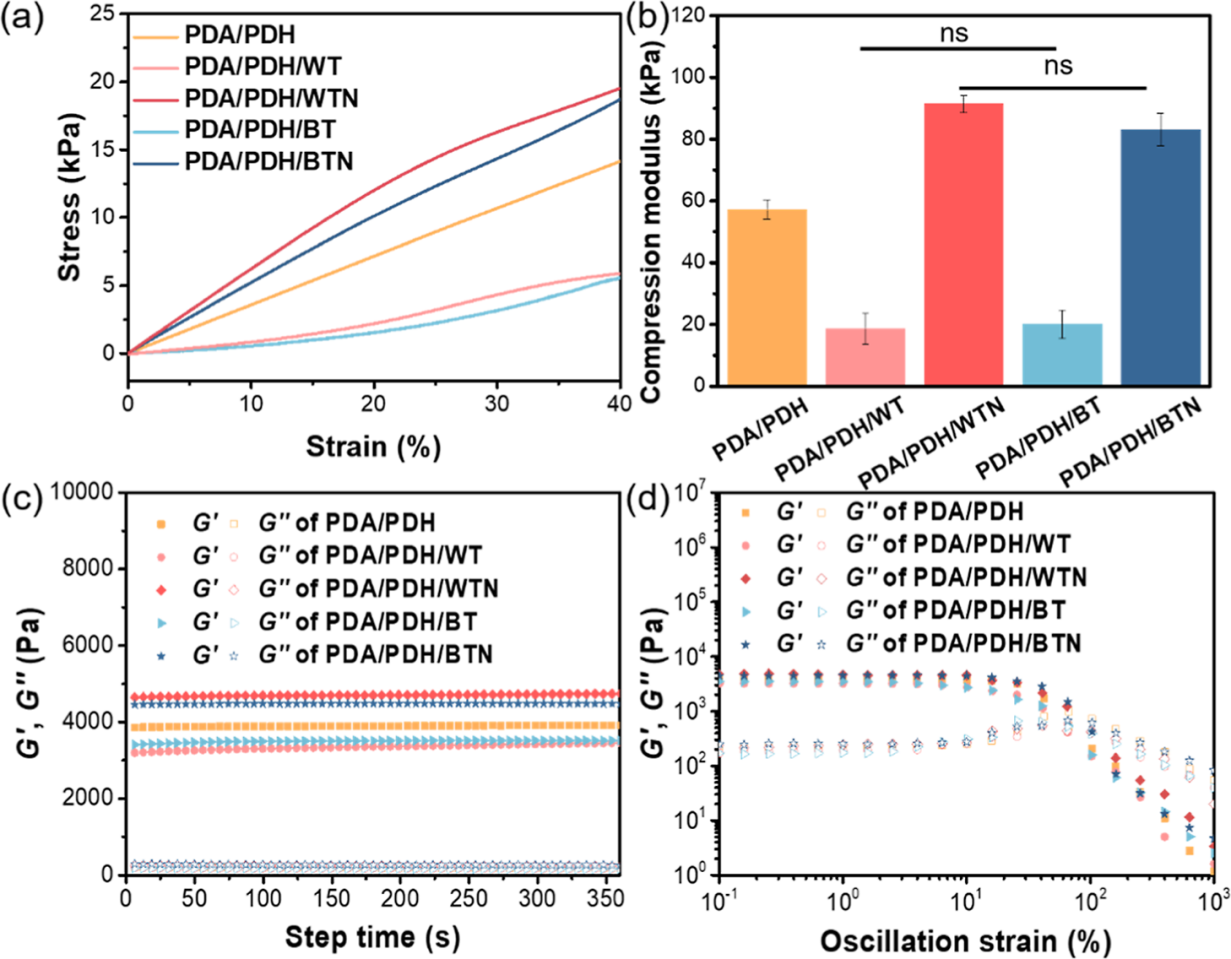
(a) Representative stress–strain curves in the compression tests of hydrogels. (b) Compression modulus, (c) oscillation time sweep, and (d) oscillation strain sweep of hydrogels.

To investigate the impact of the silica shell of bTiO_2_ nanoparticles on hydrogel mechanics, we synthesized amine-free silica-coated bTiO_2_ (BTS) and compared its compression modulus within hydrogels against those formed with bTiO_2_ (BT) and amine-functionalized bTiO_2_@MS-NH_2_ (BTN). Our findings revealed that the PDA/PDH/BTS hydrogel (∼23 kPa) possessed a compression modulus comparable to the PDA/PDH/BT hydrogel (∼20 kPa) (Figure S14). Significantly, both were mechanically weaker than the PDA/PDH/BTN hydrogel (∼89 kPa). These results confirmed that the unfunctionalized silica shell does not influence the mechanical properties of the hydrogels.

The compression and rheological studies revealed that the PDA/PDH/WT and PDA/PDH/BT hydrogels possessed relatively weak mechanical properties, which also correlated to their loose network structures observed in the structural observation. This was primarily because the nanoparticles interfered with the hydrazone-cross-linked hydrogel network, resulting in larger pore sizes that reduced the mechanical properties of the hydrogels. On the other hand, the amine-modified nanoparticles in the PDA/PDH/WTN and PDA/PDH/BTN hydrogels not only disrupted the interactions between polymers but also acted as cross-linkers to create connections between nanoparticles and polymers. Consequently, the PDA/PDH/WTN and PDA/PDH/BTN hydrogels presented superior mechanical properties than PDA/PDH/WT and PDA/PDH/BT hydrogels due to the additional imine bonds generated between the amine-modified nanoparticles and PDA.

The thermal stability of polymers and hydrogels was evaluated using thermogravimetric analysis (TGA) (Figure S15a,b). The thermal degradation temperature (*T*_d_) of PDH (∼271.3 °C) was observed to be higher than that of PDA (∼265.1 °C), which can be attributed to the resonance effect between nitrogen atoms in PDH and adjacent carbonyl groups. This resonance stabilizes the electron distribution within the bonds, increasing the bonding energy and thermal stability of PDH compared to PDA during thermal degradation.^45^ The *T*_d_ of PDA/PDH, PDA/PDH/WT, PDA/PDH/WTN, PDA/PDH/BT, and PDA/PDH/BTN hydrogels were ∼267.5, 260.3, 259.9, 261.1, and 260.4 °C, respectively. Overall, the hydrogels exhibited thermal degradation temperatures within a narrow range of 260–268 °C. The minimal variation observed among the samples might be attributed to the low concentration of nanoparticles incorporated into the hydrogel matrix.

The glass transition temperature (*T*_g_) and melting temperature (*T*_m_) of polymers and hydrogels were evaluated using differential scanning calorimetry (DSC). The *T*_g_ and *T*_m_ values of PDA were significantly higher (*T*_g_ = 65.2 °C, *T*_m_ = 129.2 °C) compared to PDH (*T*_g_ = 45.4 °C, *T*_m_ = 114.5 °C) (Figure S15c,d). The increased *T*_g_ and *T*_m_ of PDA relative to PDH can be attributed to the higher molecular weight of the polydextran used during the PDA synthesis, as generally polydextran with a higher molecular weight exhibits stronger intermolecular interactions. In the PDA/PDH hydrogel, the *T*_g_ was recorded at 49.8 °C, which fell between the *T*_g_ values of PDA and PDH, as anticipated for a combination of the two materials. The formation of hydrazone bonds in PDA/PDH hydrogel also contributed to an increase in *T*_g_, though it remained lower than PDA, which could be due to the incorporation of PDH that reduced the overall structural rigidity. The *T*_g_ values of the nanocomposite hydrogels did not exhibit significant changes and remained lower than that of PDA/PDH hydrogel. Specifically, the *T*_g_ values of PDA/PDH/WT, PDA/PDH/WTN, PDA/PDH/BT, and PDA/PDH/BTN hydrogels were 44.9, 45.0, 44.7, and 46.2 °C, respectively. These slight variations were attributed to the introduction of nanoparticles and the presence of imine bonds, which presented a minimal impact on the *T*_g_. Regarding the *T*_m_, the PDA/PDH presented a similar *T*_m_ (130.7 °C) to PDA (129.2 °C) but higher than PDH (114.5 °C). Conversely, the *T*_m_ of the PDA/PDH/WT (120.2 °C) and PDA/PDH/BT (123.1 °C) hydrogels were lower than that of PDA/PDH hydrogel (130.7 °C), suggesting that the incorporation of nanoparticles disrupted the hydrogel networks and reduced their thermal stability. However, PDA/PDH/WTN (*T*_m_ = 132.0 °C) and PDA/PDH/BTN (*T*_m_ = 149.3 °C) hydrogels exhibited higher *T*_m_ values than the PDA/PDH hydrogel (130.7 °C). This enhancement could be due to the improved interactions between the amine-modified nanoparticles and the polymeric network, which increased the thermal stability of the hydrogels. Notably, the PDA/PDH/BTN hydrogel achieved the highest *T*_g_ (46.2 °C) and *T*_m_ (149.3 °C) among the nanocomposite hydrogels, indicating that bTiO_2_@MS-NH_2_ nanoparticles provided stronger intermolecular interactions and a more robust cross-linked network, enhancing both the rigidity and thermal stability of the overall hydrogel structure.

### 3.5 Swelling Behavior, Degradation, and Stability of Hydrogels

The swelling behavior of the hydrogels was assessed by immersing the lyophilized samples in phosphate-buffered saline (PBS) solution (pH 7.4) at 37 °C. The swelling ratios for the PDA/PDH, PDA/PDH/WT, PDA/PDH/WTN, PDA/PDH/BT, and PDA/PDH/BTN hydrogels were determined to be 6.3 ± 0.3, 6.4 ± 0.7, 6.0 ± 0.4, 6.5 ± 0.2, and 5.8 ± 0.4, respectively (Figure S16a). Additionally, the water contents of the PDA/PDH, PDA/PDH/WT, PDA/PDH/WTN, PDA/PDH/BT, and PDA/PDH/BTN hydrogels were measured as 84.0 ± 0.6%, 84.1 ± 1.8%, 83.2 ± 1.1%, 84.7 ± 0.4%, and 82.7 ± 1.2%, respectively (Figure S16b). Notably, the water content in all hydrogel formulations remained above 80%, suggesting that these hydrogels possess favorable moisture retention properties. To assess the time-dependent water content of the hydrogels, we first immersed lyophilized samples in water for 2 h. Following this immersion, the hydrogels were removed from the water, and their weight was subsequently monitored for up to 6 h at 25 °C. The results demonstrated that the hydrogels exhibited less than 1% weight loss over the 6 h period, indicating their effectiveness in retaining water (Figure S16c).

The stability of the hydrogels was determined by immersing them in PBS and serum-containing media and checking their morphological changes in the macroscale. The hydrogels maintained structural integrity in both solutions after 7 days (Figure S17a). A quantitative investigation of hydrogel stability was performed by measuring the remaining mass of the hydrogels after incubating the hydrogels in PBS at 37 °C, showing the remaining mass percentages of the five hydrogels consistently remained above 70% (Figure S17b). Additionally, these hydrogels exhibited slow degradation over 30 days, indicating a high level of stability. It was noticed that there was no significant difference in degradation among the five hydrogels due to the high stability of hydrogels with hydrazone bond cross-linking.

### 3.6 Self-Healing, Shear-Thinning, and Injectable Properties of Hydrogels

The self-healing and shear-thinning behavior of the hydrogels was evaluated through rheological tests. The self-healing behavior of the hydrogel was assessed through cyclic strain time sweep measurements, applying alternating low and high strain conditions. The reversible reconstruction of the hydrogel network was evidenced by the rapid recovery of the storage modulus (*G*′) and loss modulus (*G*″) of the five hydrogels upon returning to low strain, demonstrating the self-healing capability of hydrogels (Figure S18). To quantitatively evaluate the self-healing efficiency of the PDA/PDH/BTN hydrogel, samples were physically cut and rejoined to simulate damage, followed by compression modulus measurements at various healing intervals. The self-healing efficiency was determined to be ∼10%, 80%, 91%, 93%, and 97% following 1, 2, 4, 8, and 24 h of healing, respectively, showing a continual recovery of mechanical strength (Figure S19).

The hydrogel also exhibited shear-thinning behavior as viscosity decreased when shear rates increased (Figure S20a). To further analyze this shear-thinning behavior quantitatively, the power law index was applied using the equation τ = κγ^*n*^, where τ is the shear stress (Pa), κ is the consistency coefficient (Pa·s), γ is the shear rate (s^–1^), and *n* is the power law index. Newtonian fluids are known to exhibit a power law index of 1, and as the value approaches zero, the material is classified as shear-thinning or pseudoplastic.^46^ The power law indices of the PDA/PDH, PDA/PDH/WT, PDA/PDH/WTN, PDA/PDH/BT, and PDA/PDH/BTN hydrogels were 0.102, 0.268, 0.103, 0.211, and 0.142, respectively, confirming their inherent shear-thinning properties (Figure S20b). A lower power law index indicates a stronger shear-thinning ability.^47^ These results showed that the hydrogels with amine modification nanoparticles (i.e., PDA/PDH/WTN and PDA/PDH/BTN hydrogels) exhibited better shear-thinning behavior under applied shear stress due to the reversible dynamic bonding (imine bonds) between the nanoparticles and polymers, in contrast, the unmodified nanoparticles form interference agents to impair the ability of the hydrogel to undergo shear-thinning under stress, resulting in weaker shear-thinning behavior.

To evaluate the injectability of the hydrogels, a syringe equipped with an 18G needle was used to apply a consistent shear force. The injection force was measured using a mechanical testing system, providing quantifiable data for precise comparison. The injection profiles of the hydrogels revealed an initial increase in injection force, followed by a plateau. The plateau values, representing the dynamic glide force, were recorded as ∼6.7, 5.6, 9.5, 5.8, and 8.5 N for PDA/PDH, PDA/PDH/WT, PDA/PDH/WTN, PDA/PDH/BT, and PDA/PDH/BTN hydrogels, respectively (Figure S21). The results indicated that these five hydrogels exhibited injectability, with higher injection force needed for PDA/PDH/WTN and PDA/PDH/BTN hydrogels due to their dense network structure. Overall, the combined self-healing, shear-thinning, and injectable functionalities of hydrogels make them suitable for applications such as extrusion-based 3D printing, where material recovery and dynamic cross-linked networks are essential.

### 3.7 pH-Responsiveness and Photothermal Effect of Hydrogels

The acidic environments generated during bacterial growth^48,49^ can potentially accelerate the degradation of hydrogels to facilitate the diffusion of ROS. Here, given the pH-sensitive noncovalent and dynamic covalent interactions in the hydrogel networks, PDA/PDH/BTN hydrogel was used as a representative example for the pH-responsiveness test. The rheological properties of PDA/PDH/BTN hydrogel were analyzed after immersion in PBS solutions at pH 5.5, 6.5, and 7.4, with the *G*′ values measuring ∼3080, 3940, and 4660 Pa, respectively (Figure S22). These results indicated that the hydrogels exhibited a reduced *G*′ under more acidic conditions, which may be attributed to the hydrolysis of imine and hydrazone bonds within the hydrogel network, leading to a softening of the overall structure.

The photothermal conversion of hydrogels was also investigated. For the NIR absorption of the hydrogels, the hydrogel was placed between two indium tin oxide (ITO) glass substrates for measurement, and a blank ITO background correction was performed before measuring the absorption of the hydrogels. PDA/PDH/BTN hydrogel exhibited higher absorbance than PDA/PDH and PDA/PDH/WTN hydrogels in the 800–1200 nm range due to the presence of bTiO_2_ nanoparticles (Figure S23), indicating that PDA/PDH/BTN hydrogel was capable of absorbing NIR for photothermal conversion. The results showed that the temperature of PDA/PDH/BTN hydrogel, which was immersed in PBS, was increased under NIR irradiation (808 nm) compared to PDA/PDH and PDA/PDH/WTN hydrogels (Figure 4a). Thermal images also showed that the temperature of the PDA/PDH/BTN hydrogel increased in a time-dependent manner under NIR irradiation (Figure 4b). The PDA/PDH/BTN hydrogel was further immersed in PBS solution and visually observed for temperature changes under NIR irradiation at varying power intensities (Figure 4c). The observations indicated that a higher power density of NIR irradiation resulted in an increased temperature of the PDA/PDH/BTN hydrogel, showing that temperature changes could be regulated by controlling the power intensity. Furthermore, the photothermal stability of PDA/PDH/BTN hydrogels was demonstrated under cyclic irradiation (Figure 4d). The hydrogels maintained stable performance over four heating/cooling cycles, with the temperature reaching the same level in each cycle and the curves remaining consistent. These findings revealed the excellent photothermal stability of PDA/PDH/BTN hydrogel, highlighting its significant potential for photothermal treatments.

**Figure 4:**
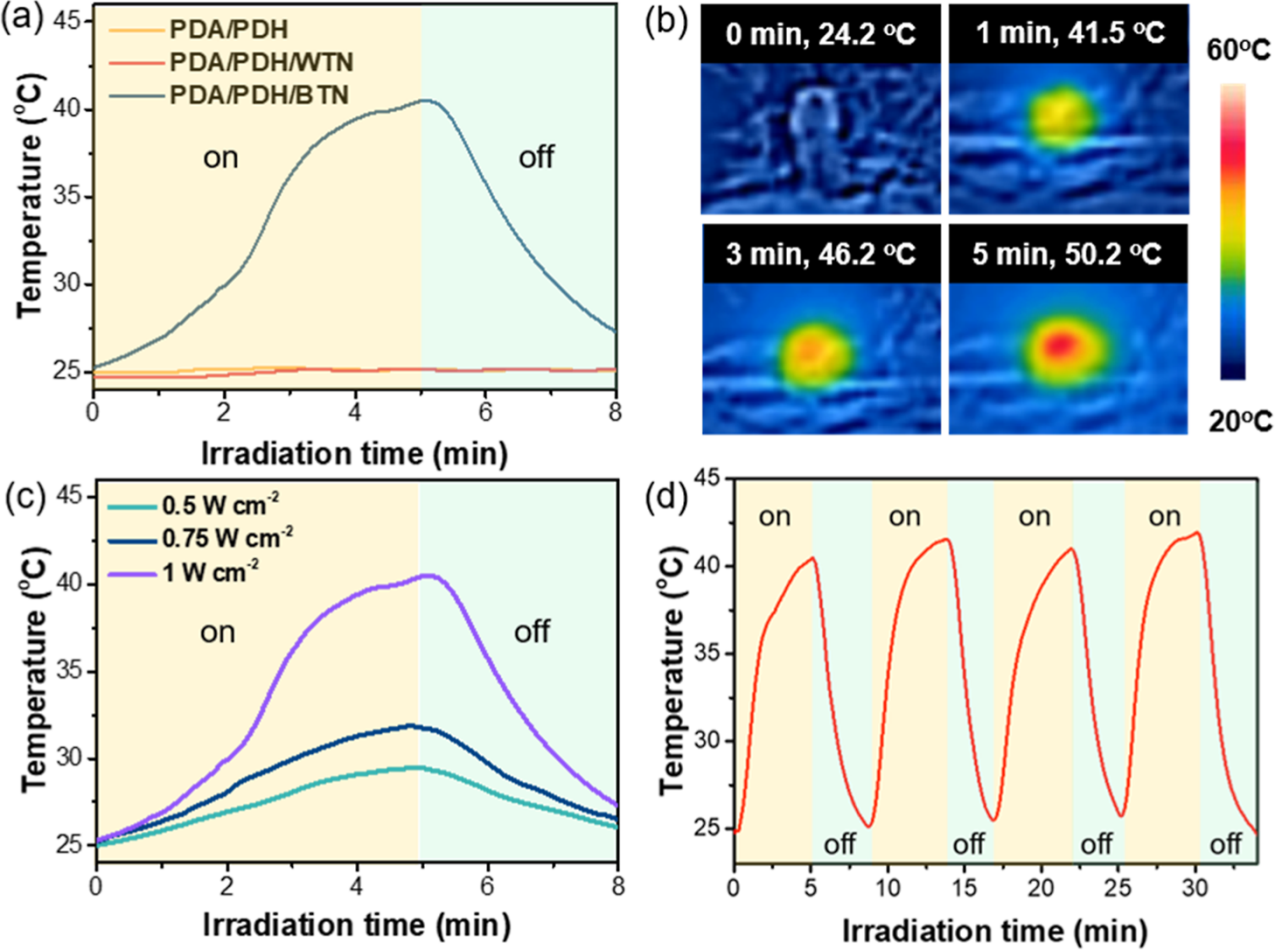
(a) Temperature curves of PDA/PDH, PDA/PDH/WTN, and PDA/PDH/BTN hydrogels under NIR irradiation (808 nm, 1 W cm^–2^). (b) Thermal images of PDA/PDH/BTN hydrogel under NIR irradiation (808 nm, 1 W cm^–2^). (c) PDA/PDH/BTN hydrogel under different power intensities of NIR irradiation (808 nm). (d) Photothermal conversion cycling test of PDA/PDH/BTN hydrogel under NIR irradiation (808 nm, 1 W cm^–2^).

### 3.8 ROS Generation of Hydrogels

The sonodynamic and photodynamic properties of hydrogels were also investigated using RNO/imidazole and MB as the probes for ^1^O_2_ and ^•^OH, respectively. When PDA/PDH, PDA/PDH/WTN, and PDA/PDH/BTN hydrogels were under US irradiation (1 MHz, 1 W cm^–2^, and 50% duty cycle), the RNO/imidazole probe solution of PDA/PDH/BTN hydrogel exhibited the lowest absorbance, indicating the highest ^1^O_2_ production (Figures 5a and S24a–c), which agreed with the high ^1^O_2_ generation efficiency of bTiO_2_@MS-NH_2_ nanoparticles. The minor absorbance decrease observed in PDA/PDH hydrogel was attributed to the generation of ^1^O_2_ via inertial cavitation bubbles induced by ultrasound, leading to a reduction in the absorbance of the probe. Additionally, PDA/PDH/BTN hydrogel can absorb 808 nm NIR light to generate ^1^O_2_ compared to PDA/PDH and PDA/PDH/WTN hydrogels (Figures 5b and S24d–f). When hydrogels were exposed to both US and NIR, a substantial drop in absorbance at 440 nm was observed on the PDA/PDH/BTN hydrogel, demonstrating the rapid production of ^1^O_2_ under the combined effects of US and NIR irradiations (Figures 5c and S24g–i). It was also noticed that PDA/PDH/BTN hydrogel produced more ^1^O_2_ when subjected to combined US and NIR irradiations than when exposed to a single type of stimulation (Figure S25a). The ^1^O_2_ generation efficiency of the PDA/PDH/BTN hydrogel under different power densities of US and NIR irradiations was also demonstrated (Figure S25b,c). The results showed that ^1^O_2_ production significantly rose when the power densities of US and NIR increased, indicating a positive correlation between ^1^O_2_ generation efficiency and energy input.

**Figure 5:**
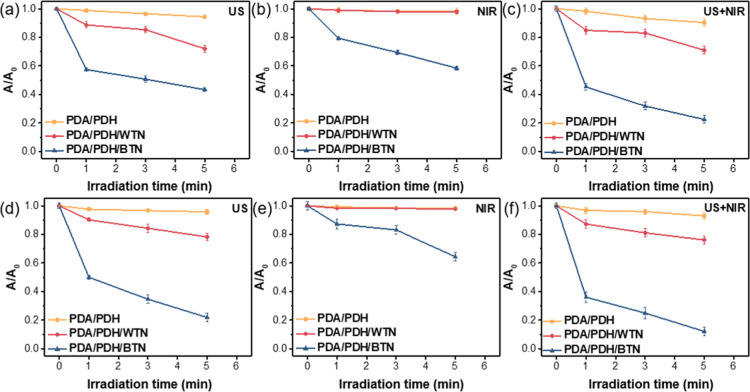
Time-dependent absorbance spectra of PDA/PDH, PDA/PDH/WTN, and PDA/PDH/BTN hydrogels reacting with RNO/imidazole under (a) US irradiation (1 MHz, 1 W cm^–2^ and 50% duty cycle), (b) NIR irradiation (808 nm, 1 W cm^–2^), and (c) both NIR and US irradiations. Time-dependent absorbance spectra of PDA/PDH, PDA/PDH/WTN, and PDA/PDH/BTN hydrogels reacting with MB under (d) US irradiation (1 MHz, 1 W cm^–2^ and 50% duty cycle), (e) NIR irradiation (808 nm, 1 W cm^–2^), and (f) both NIR and US irradiations.

PDA/PDH/BTN hydrogel also demonstrated the most pronounced reduction in the absorbance of MB when exposed to US and/or NIR compared to PDA/PDH and PDA/PDH/WTN hydrogels, indicating its greater efficiency in generating ^•^OH (Figures 5d–f and S26). Again, PDA/PDH/BTN hydrogel produced more ^•^OH under the combined US and NIR irradiations compared to the exposure with a single type of stimulation (Figure S27a). The US and NIR power densities dependence on the ^•^OH production of PDA/PDH/BTN hydrogel was also demonstrated (Figure S27b,c). These results demonstrated the superior ROS generation capability of the PDA/PDH/BTN hydrogel, highlighting its potential for sonodynamic and photodynamic therapies. It should be noted that the heat can be generated by ultrasound, showing that the temperature changes of the PBS and the hydrogels immersed in the PBS solution were ∼14 °C (Figure S28).

### 3.9 *In Vitro* Antibacterial Effect of Hydrogels

Ultrasound-based biomedical applications have been well established,^50^ including the use of ultrasound to activate mechanophores for payload release^51^ and to generate ROS for cancer therapy.^52^ The SDT mechanisms have been well discussed in the literature, showing that ultrasound primarily achieves its therapeutic effects through inertial cavitation and the generation of ROS.^38,53,54^ Under ultrasonic stimulation, inertial cavitation can be induced, in which microbubbles undergo rapid expansion and collapse, releasing a large amount of energy. This process creates localized high temperatures and pressures that can disrupt cellular membranes, denature proteins, and even lead to cell death due to strong shear forces. The collapse of inertial cavitation bubbles also initiates a series of chemical reactions that result in the generation of ROS. Two main pathways have been proposed for ROS generation. The first is sonoluminescence, where the light emitted from collapsing bubbles activates sonosensitizers, leading to the formation of electron–hole pairs. These reactive species further interact with molecular oxygen and water to produce singlet oxygen (^1^O_2_) and hydroxyl radicals (^•^OH). The second mechanism is pyrolysis, in which the high temperatures generated during inertial cavitation cause thermal decomposition of water molecules and sonosensitizers, producing free radicals that contribute to oxidative stress and cell damage. Overall, ultrasound kills bacteria through several mechanisms, including mechanical forces, thermal effects, and ROS generation. This study specifically investigates the role of ROS generated by different nanocomposite hydrogels under consistent ultrasound settings for antibacterial treatment.

The antibacterial effect of the hydrogels against *E. coli* and *S. aureus* was evaluated by immersing the hydrogels in the bacterial suspension and subjecting them to US (1 MHz, 1 W cm^–2^) and/or NIR irradiation (808 nm, 1 W cm^–2^) (Figure 6a). The colony-forming unit (CFU) counting result for PDA/PDH hydrogel was similar to the control group under the treatments, potentially due to PDA/PDH hydrogel not having inherent antibacterial properties. It was also noticed that the control and PDA/PDH groups exhibited a significant bacterial reduction in the CFU counts under US and US + NIR treatments, which should be due to the inertial cavitation bubbles and shear stress caused by ultrasound to damage the bacteria. On the other hand, PDA/PDH/WTN hydrogel, which contained wTiO_2_ nanoparticles that responded to the US but not to NIR, showed a lower bacterial survival rate compared to the control and PDA/PDH groups under US and US + NIR treatment due to more ROS produced to kill bacteria. Overall, PDA/PDH/WTN hydrogel presented lower CFU counts in the US (7.2 log CFU mL^–1^ for *S. aureus* and 8.2 log CFU mL^–1^ for *E. coli*) and US + NIR treatment (7.2 log CFU mL^–1^ for *S. aureus* and 8.2 log CFU mL^–1^ for *E. coli*) groups compared to the nontreatment (7.6 log CFU mL^–1^ for *S. aureus* and 9.4 log CFU mL^–1^ for *E. coli*) and NIR treatment (7.6 log CFU mL^–1^ for *S. aureus* and 9.3 log CFU mL^–1^ for *E. coli*) groups.

**Figure 6:**
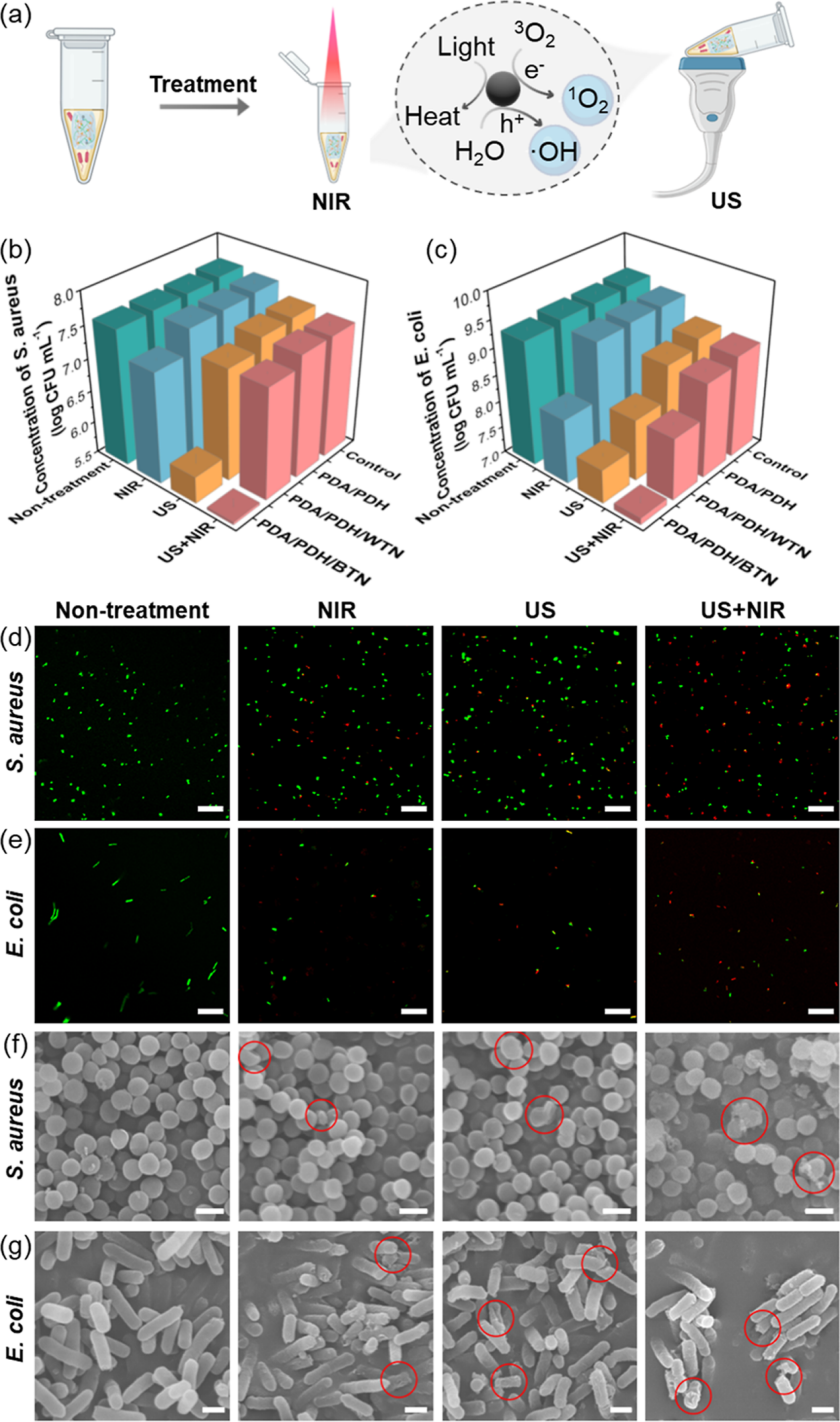
*In vitro* antibacterial efficacy of hydrogels. (a) Schematic illustration of the mechanism of hydrogels for antibacterial activity under US and NIR irradiation. Quantitative analysis of the antibacterial effect of (b) *S. aureus* and (c) *E. coli* through the spread plate method. Live/dead staining of (d) *S. aureus* and (e) *E. coli* under different treatments of PDA/PDH/BTN (scale bar: 20 μm). SEM images of (f) *S. aureus* and (g) *E. coli* under different treatments of PDA/PDH/BTN (scale bar: 1 μm). US: treat by ultrasound (1 MHz, 1 W cm^–2^, and 50% duty cycle) for 5 min; NIR: treat by 808 nm laser (1 W cm^–2^) for 5 min. Red circles indicate areas of cell membrane disruption.

For PDA/PDH/BTN hydrogel, there was no significant difference in the CFU counts between the nontreatment and the control groups, with ∼7.7 log CFU mL^–1^ for *S. aureus* and ∼9.2 log CFU mL^–1^ for *E. coli* (Figures 6b,c and S29, S30). In contrast, the ROS and heat generated by PDA/PDH/BTN hydrogel under US and/or NIR treatment could effectively inhibit bacterial growth. It was noticed that the temperature of PDA/PDH/BTN hydrogel immersed in the bacteria suspension rose to ∼45 °C upon NIR irradiation for 5 min, showing the promise of photothermal treatment (Figure S31). For the NIR treatment, the CFU counts of *S. aureus* and *E. coli* were decreased to 7.2 and 8.2 log CFU mL^–1^, respectively. For the US treatment, the CFU counts of *S. aureus* and *E. coli* were reduced to 5.9 and 7.6 log CFU mL^–1^, respectively. Further analysis revealed that the antibacterial effect of the US treatment group was superior to that of the NIR treatment group. This can be attributed to the fact that US stimulation induces a more effective ROS generation than NIR irradiation, which was consistent with the ROS generation analysis. Furthermore, under the synergistic effect of US and NIR (US + NIR treatment), very few bacterial colonies were observed on the agar plate (Figure S32), with quantification revealing CFU counts of *S. aureus* and *E. coli* were 5.5 and 7.1 log CFU mL^–1^, respectively. The results confirmed that the synergistic effect of US and NIR treatment generated significantly higher ROS levels to enhance the antibacterial efficacy of PDA/PDH/BTN hydrogel.

Live/dead bacterial analysis was also performed to investigate the extent of bacterial death by PDA/PDH/BTN hydrogel under different treatments (Figure 6d,e). The nontreatment group exhibited only green fluorescence, while increased red fluorescence was observed under both US and NIR treatments. The US + NIR group showed the most intense red fluorescence, confirming the superior antibacterial effect of PDA/PDH/BTN hydrogel under the cotreatment of US and NIR. Furthermore, SEM was employed to evaluate the antibacterial effect of PDA/PDH/BTN hydrogel under the combined irradiation of US and NIR (Figure 6f–g). The surface of bacteria subjected to US or NIR treatment showed significant wrinkling and distortion, while the structural dissolution of the bacterial surface was more pronounced in the US + NIR treatment group. These findings confirmed that ROS and heat generated by PDA/PDH/BTN hydrogel under US + NIR treatment caused oxidative and heat damage to the bacterial cell, leading to cell membrane rupture and even complete cell collapse.^55–57^

### 3.10 *Ex Vivo* Antibacterial Effect of Hydrogels

*Ex vivo* models serve as a bridge between *in vitro* and *in vivo* studies, providing a biologically relevant environment for evaluating therapeutic efficacy.^58^ Here, an *ex vivo* porcine skin model was employed to assess the antibacterial efficacy of PDA/PDH/BTN hydrogel (Figures 7a and S33). To simulate skin injuries prone to bacterial infections, a standardized wound model was established by creating circular wounds on the porcine skin, and then the bacterial suspension was introduced onto the wounds. The antibacterial efficacy of PDA/PDH/BTN hydrogel was evaluated by quantifying the bacterial load within the wound area under different treatment conditions.

**Figure 7:**
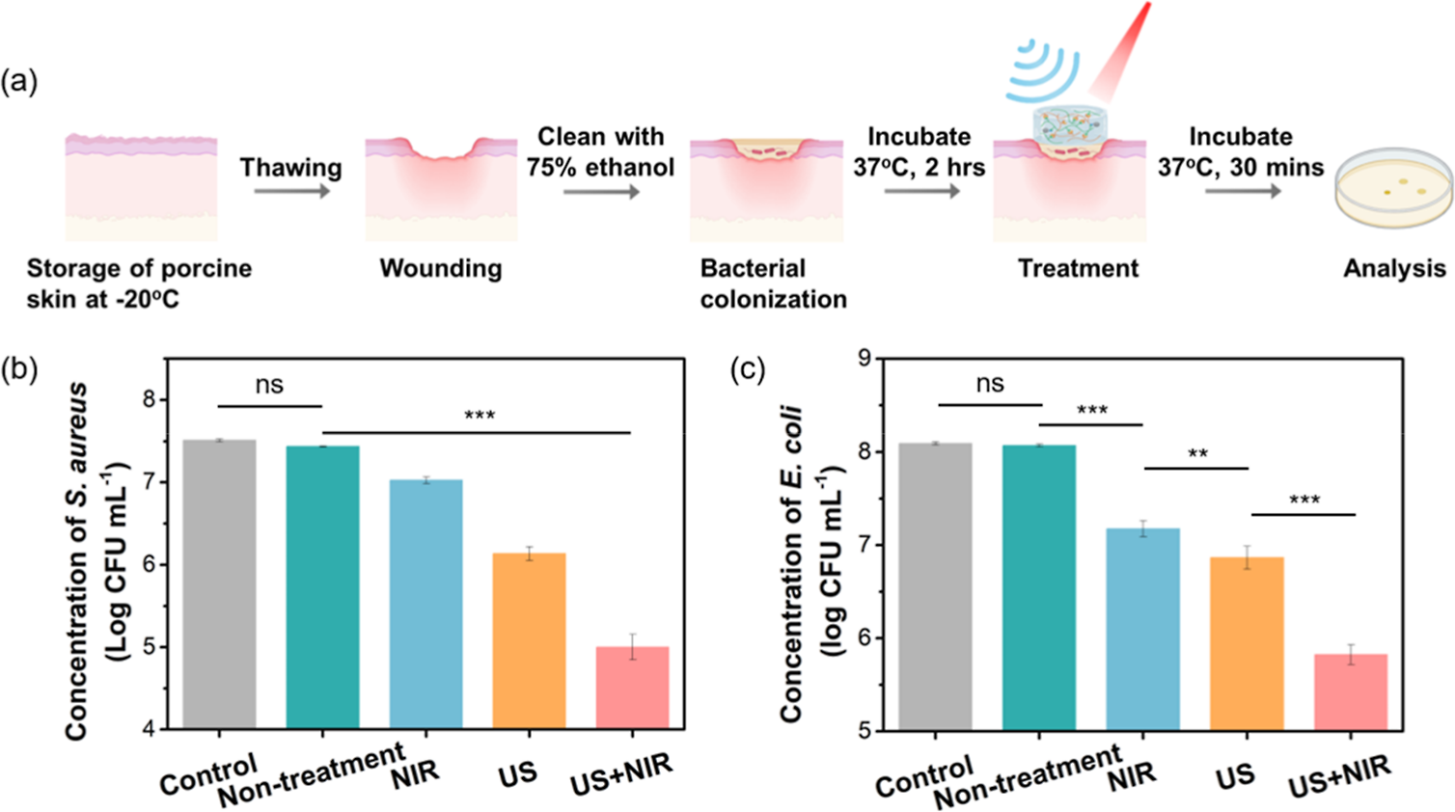
*Ex vivo* antibacterial efficacy of PDA/PDH/BTN. (a) Schematic illustration demonstrating the experimental setup and workflow of the *ex vivo* wound model. Quantitative analysis of the antibacterial effect of (b) *S. aureus* and (c) *E. coli* through the spread plate method. US: treat by ultrasound (1 MHz, 1 W cm^–2^, and 50% duty cycle) for 5 min; NIR: treat by 808 nm laser (1 W cm^–2^) for 5 min. Significant results were indicated: **p < 0.01, ***p < 0.001, and ns for no significant difference.

The *ex vivo* results demonstrated that the CFU counts of bacteria on the wounds of the nontreatment group were slightly lower than those in the bacterial suspension group, showing *S. aureus* and *E. coli* exhibited a bacterial load exceeding 7.4 and 8.1 log CFU mL^–1^, respectively. This observation can be attributed to the porous structure of the hydrogel, which may absorb a small fraction of the bacterial suspension to cause a slight reduction in CFU counts (Figures 7b,c and S34). Upon US and/or NIR treatment, the PDA/PDH/BTN hydrogel generated ROS and heat to inhibit bacterial growth effectively. For NIR treatment samples, the CFU counts of *S. aureus* and *E. coli* were reduced to 7.0 and 7.2 log CFU mL^–1^, respectively. Similarly, under US irradiation, the CFU counts of *S. aureus* and *E. coli* decreased to 6.1 and 6.8 log CFU mL^–1^, respectively. Notably, bacterial colonies were nearly undetectable on agar plates under US + NIR treatment, with CFU quantification revealing a significant reduction in *S. aureus* (5 log CFU mL^–1^) and *E. coli* (5.8 log CFU mL^–1^) (Figures 7b,c and S34). These results confirmed that the synergistic effect of US and NIR irradiation led to a substantially higher ROS generation to enhance the antibacterial efficacy of PDA/PDH/BTN hydrogel.

The PDA/PDH/BTN hydrogel, by taking advantage of the US- and NIR-responsive properties of bTiO_2_ nanoparticles, simultaneously exhibited SDT, PDT, and PTT, making it significantly more effective than single-modal treatments in antibacterial applications. In SDT, the US stimulates ROS generation, which leads to oxidative stress that impairs bacterial metabolism, causing cell membrane damage and rupture. In PDT and PTT, PDA/PDH/BTN hydrogel produces ROS and generates localized heating under NIR irradiation, disrupting bacterial cell structures and reducing bacterial viability. Therefore, the synergistic SDT/PDT/PTT effect significantly reduces bacterial survival rates, with visible membrane shrinkage and structural disintegration, confirming the potent antibacterial efficacy of PDA/PDH/BTN hydrogel. Compared to conventional single-modal PDT or PTT hydrogel systems using the NIR-absorbing agents (e.g., graphene oxide^59,60^ and Au-modified nanohydroxyapatite^61^), PDA/PDH/BTN hydrogel not only maximizes ROS production through multiple mechanisms but also enhances bacterial membrane permeability through the acoustic effects of SDT, thus improving the overall efficacy of treatments. This synergistic SDT/PDT/PTT combined approach provides superior bactericidal activity and reduces the probability of bacterial resistance, positioning PDA/PDH/BTN hydrogel as a highly promising and innovative antibacterial material.

Additionally, PDA/PDH/BTN hydrogel exhibited a stronger antibacterial effect against *S. aureus* than against *E. coli*. Based on *in vitro* results, *S. aureus* and *E. coli* showed a 2.2-log and 2.1-log reduction after US + NIR treatment, respectively. Similarly, in *ex vivo* tests, *S. aureus* experienced a 2.4-log reduction, compared to a 2.3-log reduction for *E. coli*. This difference was primarily attributed to the structural distinctions between Gram-positive and Gram-negative bacterial cell walls. *S. aureus*, as a kind of Gram-positive bacteria, has a relatively simple cell envelope composed of a thick peptidoglycan layer and teichoic acids surrounding the cytoplasmic membrane. On the other hand, *E. coli* is a kind of Gram-negative bacteria that possesses a more complex and multilayered cell wall structure. The outer membrane of *E. coli* comprises a lipid bilayer containing lipopolysaccharides (LPS), which serves as an additional protective barrier to enhance their resistance to hostile environmental conditions.^62^

A key advantage of SDT over PDT is its ability to activate sonosensitizers and generate ROS in deep tissues.^63–65^ To further evaluate this capability, we conducted ROS measurements by covering the sample tube with layers of pork skin to simulate tissue penetration and compared the ROS generation under either SDT or PDT conditions. Here, three conditions were evaluated: no pig skin, a single layer, and a double layer of pig skin covering the sample tubes (Figure S35a). The results demonstrated that under US irradiation (SDT), the efficiency of ^1^O_2_ and ^•^OH generation by PDA/PDH/BTN hydrogel slightly decreased with increasing skin thickness (Figure S35b,c). For example, the ^•^OH generation by PDA/PDH/BTN hydrogel was reduced to 57% and 48% after covering one (∼2.8 mm) and two layers (∼7.2 mm) of pig skin, respectively. Notably, even with two layers of pig skin, PDA/PDH/BTN hydrogel generated a higher ^•^OH amount under SDT compared to that under NIR irradiation (PDT) without any skin coverage. These results indicate that SDT possesses superior tissue-penetrating ability and ROS generation efficiency compared to PDT, highlighting its potential for effective deep-tissue therapeutic applications.

Previously reported SDT/PDT/PTT antibacterial treatments are fabricated using nanomaterials as therapeutic agents. For instance, Cheng et al. fabricated a dispersion of nanoparticles as SDT/PDT/PTT antibacterial agents, where polydopamine was involved in forming mesoporous TiO_2_@polydopamine nanoparticles.^22^ The mesoporous TiO_2_@polydopamine nanoparticles have been demonstrated to generate ROS and heat under 808 nm laser and ultrasound irradiation to perform SDT/PDT/PTT effects and accelerate wound healing. Liu et al. develop CuS/curcumin particles for SDT/PDT/PTT by using a photosono interfacial engineering strategy to allow the particles to exhibit enhanced electron–hole separation to kill both *S. aureus* and *E. coli* under 808 nm NIR light and ultrasound.^23^ However, these nanomaterial-based SDT/PDT/PTT systems face the challenges of diffusing beyond the target area, which may reduce their therapeutic effectiveness and raise toxicity concerns. This study pioneers the use of hydrogels as carriers for inorganic photo/sono-sensitizers in the synergistic SDT/PDT/PTT antibacterial treatment. Hydrogels offer a stable platform for nanomaterials and can be directly applied as wound dressings to localize treatment at the infection site and be easily removed once therapy is completed, enhancing treatment safety and effectiveness. Furthermore, PDA/PDH/BTN hydrogel with a dynamic cross-linked network exhibited unique self-healing and injectability features, advancing SDT/PDT/PTT antibacterial treatments beyond existing nanomaterial-based systems. The self-healing property of the PDA/PDH/BTN hydrogels allows this therapeutic platform to recover from damage, extending its usable life. The injectability of the PDA/PDH/BTN hydrogels enables their application in diverse environments, including deep-infected tissues, and allows them to be molded into various shapes, increasing their versatility across different medical conditions. Finally, the biocompatibility and moisture-retaining properties of hydrogels are desirable for applications in wound care, facilitating faster and more effective healing after the antibacterial treatment. Therefore, PDA/PDH/BTN hydrogels present a promising alternative to traditional functional nanomaterials for SDT/PDT/PTT antibacterial treatments. Besides, this study also broadens the versatility of the TiO_2_-containing nanocomposite hydrogels by enabling them to generate ROS under NIR irradiation for effective antibacterial action.^66,67^ The NIR-responsive TiO_2_-containing nanocomposite hydrogels effectively compensate for the limitations of the US-based therapy, specifically its poor focusing ability and the requirement for direct contact, improving treatment precision and efficiency in killing bacteria. Overall, these features strongly support the potential of PDA/PDH/BTN hydrogel for advanced antibacterial wound dressings and infection control applications to effectively eliminate bacteria while avoiding the use of antibiotics, significantly reducing the risk of bacterial resistance and overcoming the limitations of single-mode therapy that may lead to bacterial adaptation.

Overall, hydrogel structures have attracted considerable attention for antibacterial applications owing to their distinctive physicochemical properties. Their high water content allows hydrogels to maintain a moist environment conducive to wound healing, while also serving as a physical barrier to bacterial invasion.^68^ Furthermore, hydrogels can be engineered to possess intrinsic antibacterial activity^69^ or be loaded with antibacterial agents^70^ to enhance efficacy. Notably, stimuli-responsive hydrogels enable controlled drug release in response to environmental cues and can be coloaded with multiple antibacterial agents to achieve synergistic therapeutic effects.^71,72^ Despite these advantages, several limitations persist. Hydrogels with insufficient mechanical strength may be unsuitable for application in load-bearing or highly dynamic regions. Additionally, rapid or unpredictable degradation can compromise structural integrity and reduce therapeutic effectiveness at the treatment site. Their antibacterial action is generally limited to surface-level activity, which may be inadequate for addressing bacteria embedded within deep tissues or biofilms. Moreover, many hydrogels are sensitive to environmental factors such as drying, temperature fluctuations, and sterilization methods, presenting challenges for long-term storage and clinical implementation.

In most current biomedical research, the combined application of PTT, PDT, and SDT, activated via light and ultrasound, has demonstrated superior therapeutic efficacy compared to any single modality alone.^23,73–76^ This enhancement has been claimed as synergistic interactions rather than mere additive effects. Each modality operates through distinct but complementary mechanisms, enabling them to overcome individual limitations and significantly amplify the overall antibacterial response. For example, PTT-induced heat enhances bacterial cell membrane permeability, facilitating deeper penetration of ROS generated by PDT and SDT.^77,78^ Conversely, ROS-mediated damage from PDT and SDT might also increase the susceptibility of bacteria to thermal stress induced by PTT. Additionally, SDT contributes mechanical forces through ultrasound-induced inertial cavitation,^79^ which not only disrupts bacterial membranes and aggregates but also promotes oxygen diffusion,^80^ further intensifying the effects of PDT and SDT to reach deeper infected areas and eradicate otherwise shielded bacterial populations. Overall, the triple-modal antibacterial therapy facilitates the simultaneous or sequential application of heat, oxidative stress, and mechanical disruption to target multiple essential bacterial structures and metabolic pathways. This multifaceted assault also significantly reduces the likelihood of resistance development, offering a promising strategy in the face of the growing antimicrobial resistance crisis.

### 3.11 *In Vitro* Cytocompatibility of Hydrogels

Mouse embryonic fibroblasts (MEFs) were employed as a cellular model to evaluate the *in vitro* cytocompatibility of PDA/PDH, PDA/PDH/WTN, and PDA/PDH/BTN hydrogels. Hydrogel extracts were obtained by incubating the materials in serum-supplemented media for 24 h before their use in cell culture assays. The alarmBlue assay revealed a progressive increase in metabolic activity of MEFs across all samples over time (Figure 8a), and the live/dead fluorescent staining also showed that the MEFs continued to proliferate for up to 5 days (Figure 8b), with live cells appearing in green and dead cells in red. These results confirm the biocompatibility of these hydrogels, supporting their potential application in biomedical fields.

**Figure 8:**
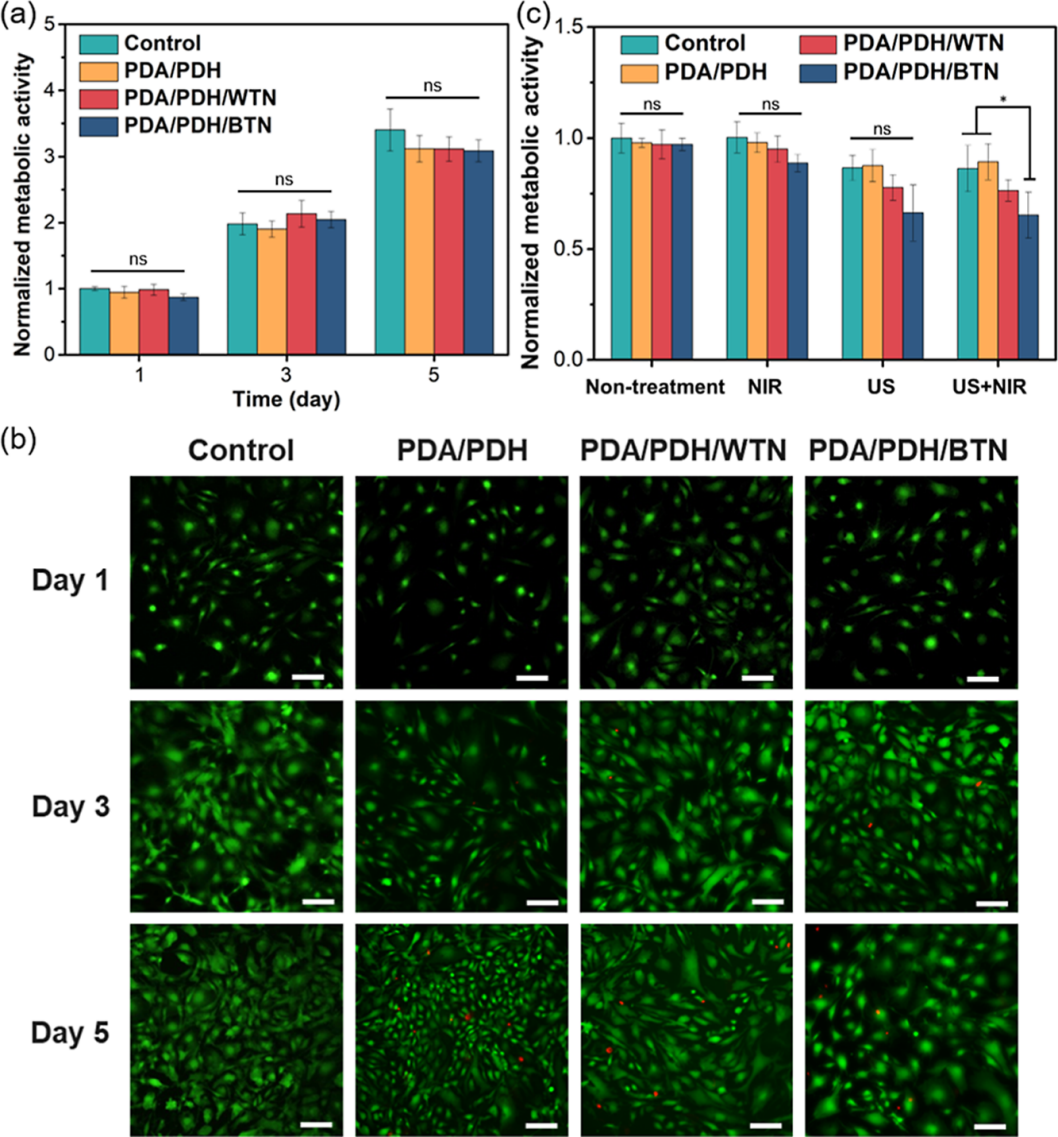
Cytocompatibility tests of the hydrogels. (a) Normalized metabolic activity of MEFs cultured in the extract solutions of the hydrogels. (b) Live/dead staining images of MEFs cultured in the extract solutions of the hydrogels, with calcium-AM labeling live cells in green and ethidium homodimer-1 labeling dead cells in red (scale bar: 100 μm). (c) Normalized metabolic activity of MEFs cultured on the surface of hydrogels under different treatments. Data are presented as mean and standard deviation (*n* = 4). Significant results were indicated: *p < 0.05 and ns for no significant difference.

To evaluate the cytocompatibility of different hydrogel compositions under various stimulation conditions, the hydrogels were coated with collagen before cell seeding to facilitate cell adhesion on the hydrogel surface. In the nontreatment condition, all groups exhibited excellent cell viability under the nontreatment condition, indicating good compatibility regardless of hydrogel composition (Figure 8c). Upon exposure to NIR irradiation, most groups maintained metabolic activity levels comparable to the control group, suggesting that NIR alone does not exhibit significant cytotoxicity. A slight decrease in cell viability was observed in the PDA/PDH/BTN group, likely attributed to localized heating and ROS generation induced by the material under NIR stimulation. Nevertheless, no statistically significant differences were observed among the groups under NIR conditions. Under US treatment, a slight reduction in cell viability was observed in both the PDA/PDH/WTN and PDA/PDH/BTN groups. However, this difference was not statistically significant, suggesting that while ultrasound may introduce mild stress through acoustic inertial cavitation, localized heating, and shear forces, these effects were not sufficient to cause significant cytotoxicity among the tested groups. The observed decrease in the PDA/PDH/WTN and PDA/PDH/BTN groups may be attributed to their capacity for ROS generation. In contrast, when combined US and NIR stimulation was applied, a statistically significant reduction in cell viability was observed in the PDA/PDH/BTN group compared to other groups (*p < 0.05), indicating that the additional effects of ROS production and thermal effect resulted in the highest level of cell damage under this condition.

## 4 Conclusion

In this study, the limitation of the TiO_2_-containing nanocomposite hydrogels requiring UV excitation for ROS generation was effectively addressed by introducing bTiO_2_ nanoparticles as innovative sensitizers. The bTiO_2_ nanoparticles in the hydrogel matrix presented increased ROS production for SDT and PDT under ultrasound and NIR stimulation due to a reduced recombination rate of electron–hole pairs. Also, the bTiO_2_-containing nanocomposite hydrogels can perform photothermal conversion under NIR irradiation for PTT. Particularly, incorporating amine-modified bTiO_2_ nanoparticles into the PDA/PDH hydrogels through imine bonds significantly improved the mechanical properties and stability of the network. Our comprehensive investigation also revealed that PDA/PDH/BTN hydrogel outperformed PDA/PDH/WTN hydrogel regarding ROS generation, photothermal conversion, and antibacterial activity. The cytocompatibility test also showed that PDA/PDH/BTN hydrogel was a nontoxic candidate for biomedical applications. Taken together, we have developed the PDA/PDH/BTN nanocomposite hydrogels with dual external stimuli responsiveness (i.e., ultrasound and NIR) and dynamic features (i.e., self-healing and injectability) as an all-in-one TiO_2_-containing hydrogel platform for SDT/PDT/PTT triple-modal therapy in the bacteria treatment. The consistent antibacterial results observed across our *in vitro* and *ex vivo* studies, along with findings from relevant *in vivo* literature,^81^ −^82,83,84^ support the potential of our hydrogels for therapeutic use, while *in vivo* wound healing assessments would still be needed to facilitate the utility of this hydrogel system in clinical application. Moreover, while this study specifically focuses on the role of ROS generated by nanocomposite hydrogels for antibacterial applications, future research should also explore other ultrasound-induced factors (e.g., acoustic inertial cavitation, shear stress, and thermal effects) that may contribute to hydrogel-based antibacterial therapy.

## Supplementary Material



## Notes

^1^H NMR and FT-IR spectra of polydextran, PDA, PDH: Figures S1 and S2. EPR, XRD, absorption spectra of nanoparticles: Figure S3. SEM and TEM–EDS elemental mapping analyses of nanoparticles: Figures S4 and S5. Sedimentation behavior and hydrodynamic diameter distributions: Figures S6 and S7. XPS spectra of bTiO_2_@MS-NH_2_ nanoparticles: Figure S8. Thermal images and thermocouple readings of nanoparticles under NIR irradiation: Figure S9. ROS assays (RNO/imidazole, MB) under NIR and US irradiation: Figure S10. Mechanical properties of hydrogels: Figure S11. FT-IR spectra and SEM–EDS elemental mapping of hydrogels: Figures S12 and S13. Compression modulus, TGA and DSC analyses: Figures S14 and S15. Swelling, stability, and degradation tests of hydrogels: Figures S16 and S17. Self-healing properties of hydrogels: Figures S18 and S19. Viscosity and injection profiles of hydrogels: Figures S20 and S21. Rheological changes under different pH: Figure S22. UV–vis–NIR spectra and ROS generation under irradiation: Figure S23–S27. Thermal imaging of hydrogels under US irradiation: Figure S28. Antibacterial efficacy against *S. aureus* and *E. coli* (*in vitro* and *ex vivo*): Figure S29–S34. ROS generation under NIR and US irradiation through porcine skin: Figure S35. Hydrodynamic diameter and PDI of nanoparticles: Table S1. Micro-CT analysis of pore size and porosity of hydrogels: Table S2. MIP analysis of pore volume, size, and porosity of hydrogels: Table S3. Rheological properties of hydrogels: Table S4 (PDF)
