# Supplementary material for: Ultrasound- and NIR-Responsive Polydextran/Black TiO2 Nanocomposite Hydrogels for Triple-Modal Antibacterial Therapy
Source: ACS Appl Mater Interfaces. 2025 Aug 19;17(35):49910–29. doi: 10.1021/acsami.5c15341 (PMC12412109; doi:10.1021/acsami.5c15341)
Supplement: Supplementary file 1 [file am5c15341_si_001.pdf]

Supporting Information

Ultrasound- and NIR-responsive polydextran/black TiO_2 nanocomposite hydrogels for triple-modal antibacterial therapy

Tzu-Ying Wang[a], Yu-Ning An[a] and Yi-Cheun Yeh[a*]

[a] Institute of Polymer Science and Engineering, National Taiwan University, Taipei 10617, Taiwan

*Corresponding authors:

Yi-Cheun Yeh, E-mail: yicheun@ntu.edu.tw

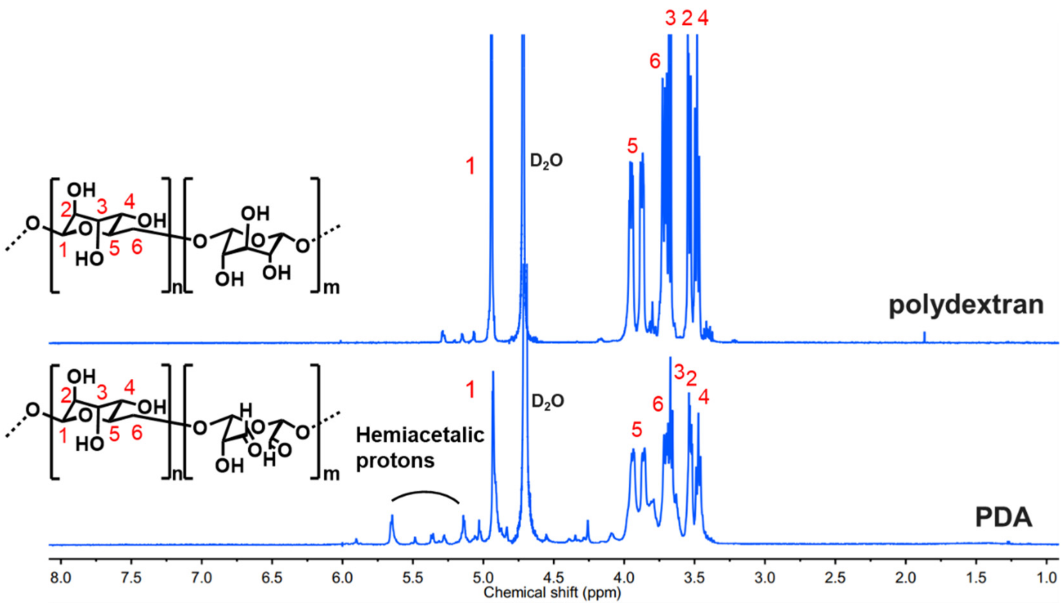

Figure S1. ^{1}H NMR spectra of polydextran and PDA.

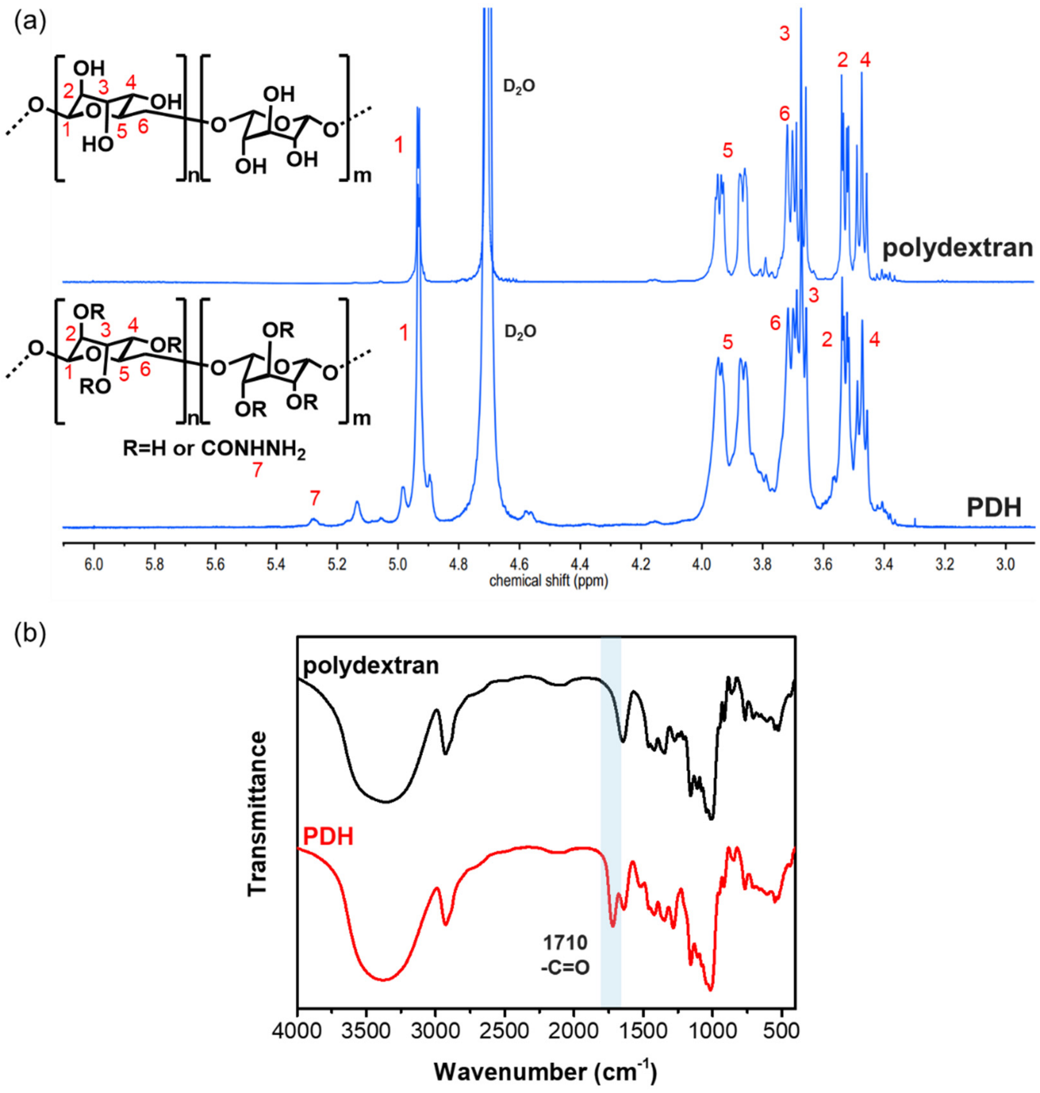

Figure S2. (a) ^{1}H NMR and (b) FT-IR spectra of polydextran and PDH.

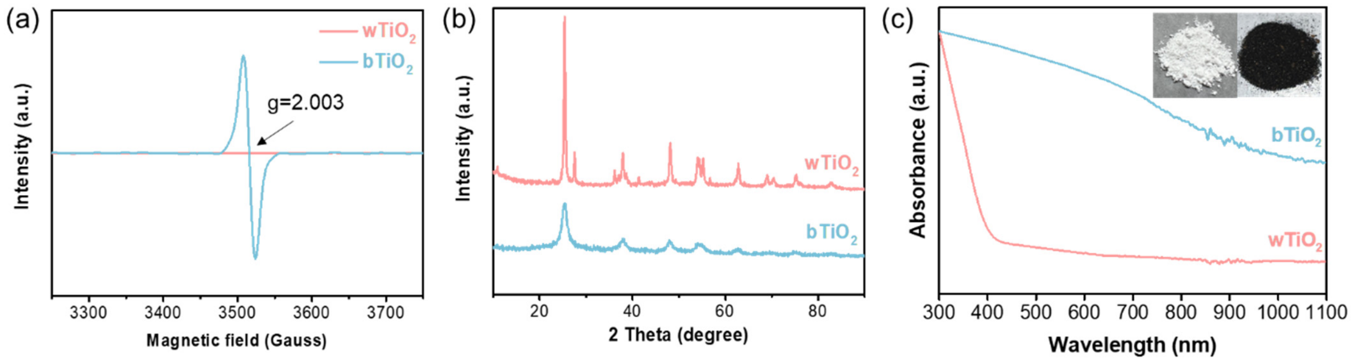

Figure S3. (a) EPR, (b) XRD, and (c) absorption spectra of nanoparticles.

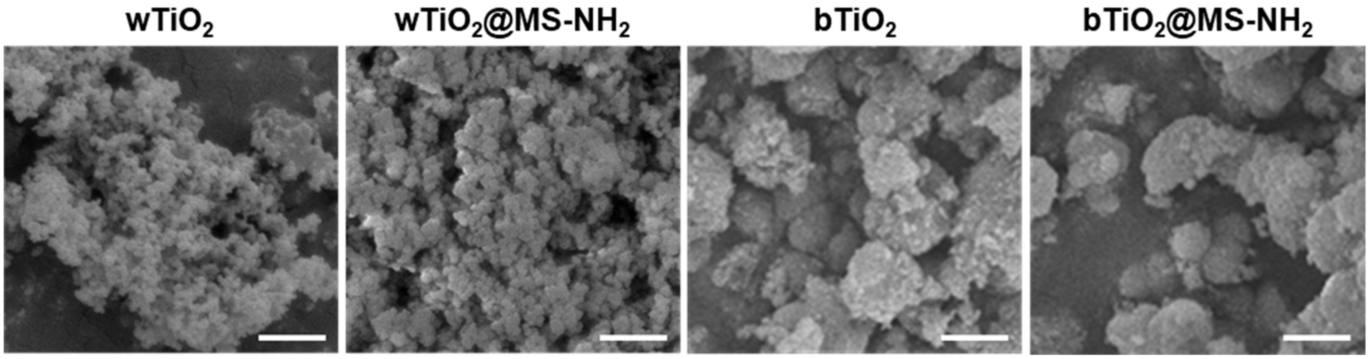

Figure S4. SEM images of nanoparticles (scale bar: 500 nm).

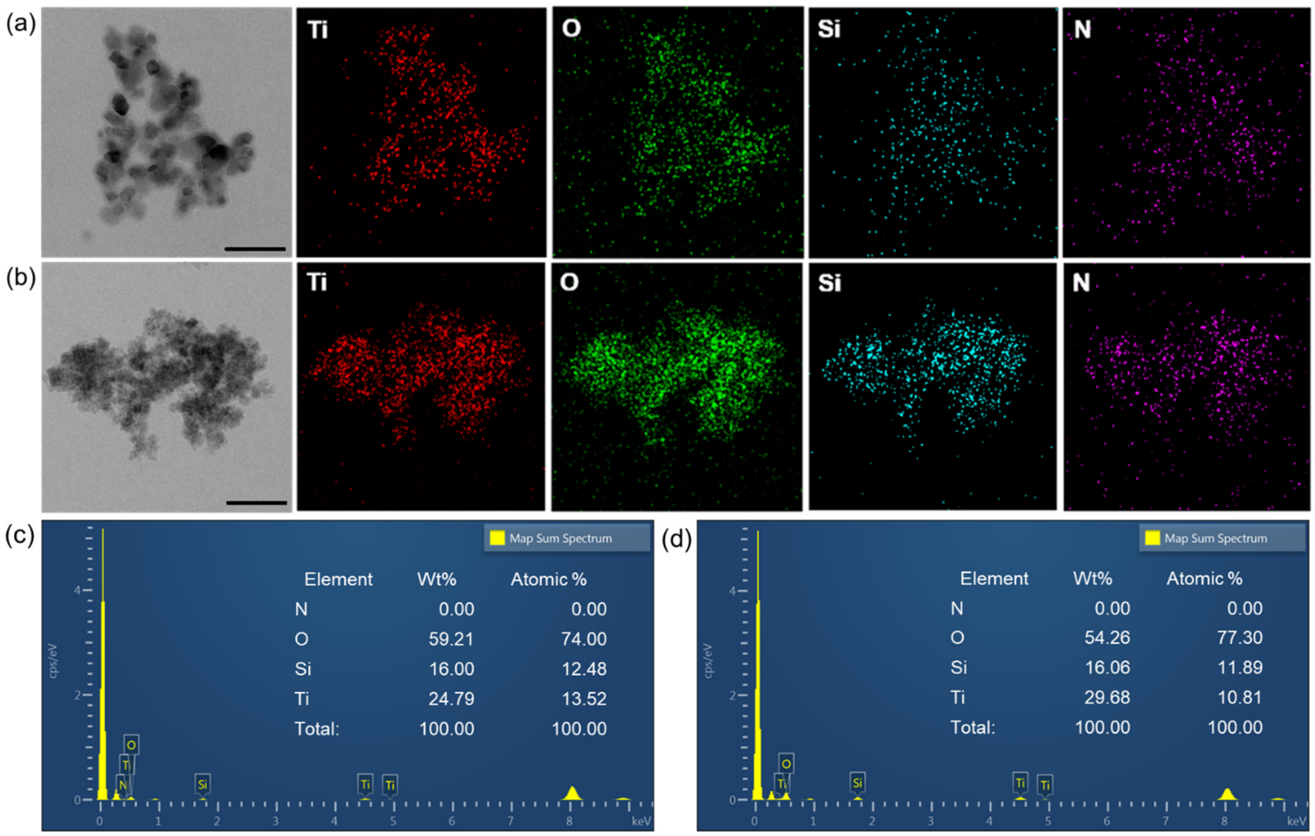

Figure S5. The elemental mapping analysis of (a) $wTiO_2@MS-NH_2$ and (b) $bTiO_2@MS-NH_2$ nanoparticles (scale bar: 100 nm). The TEM-EDS analysis of (c) $wTiO_2@MS-NH_2$ and (d) $bTiO_2@MS-NH_2$ nanoparticles.

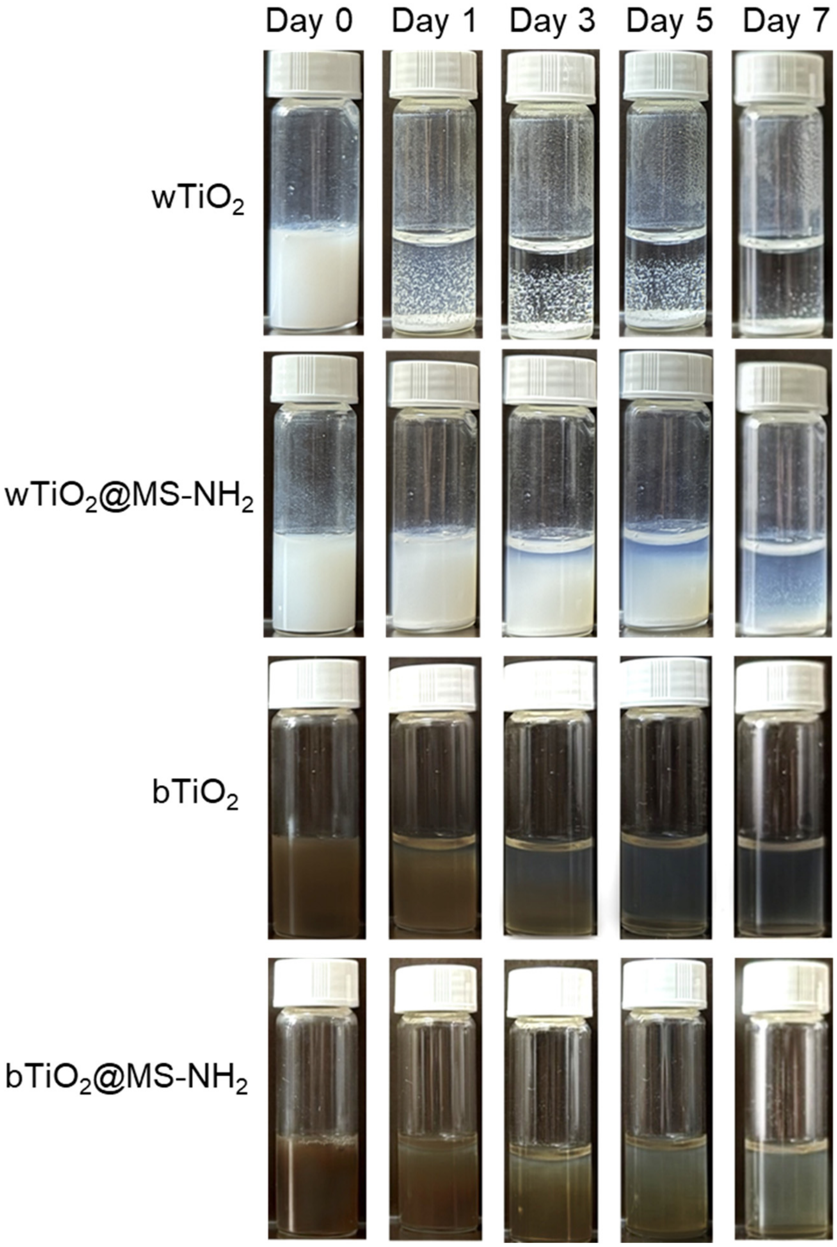

Figure S6. Sedimentation behavior of nanoparticles at a concentration of 1 mg mL^{-1}.

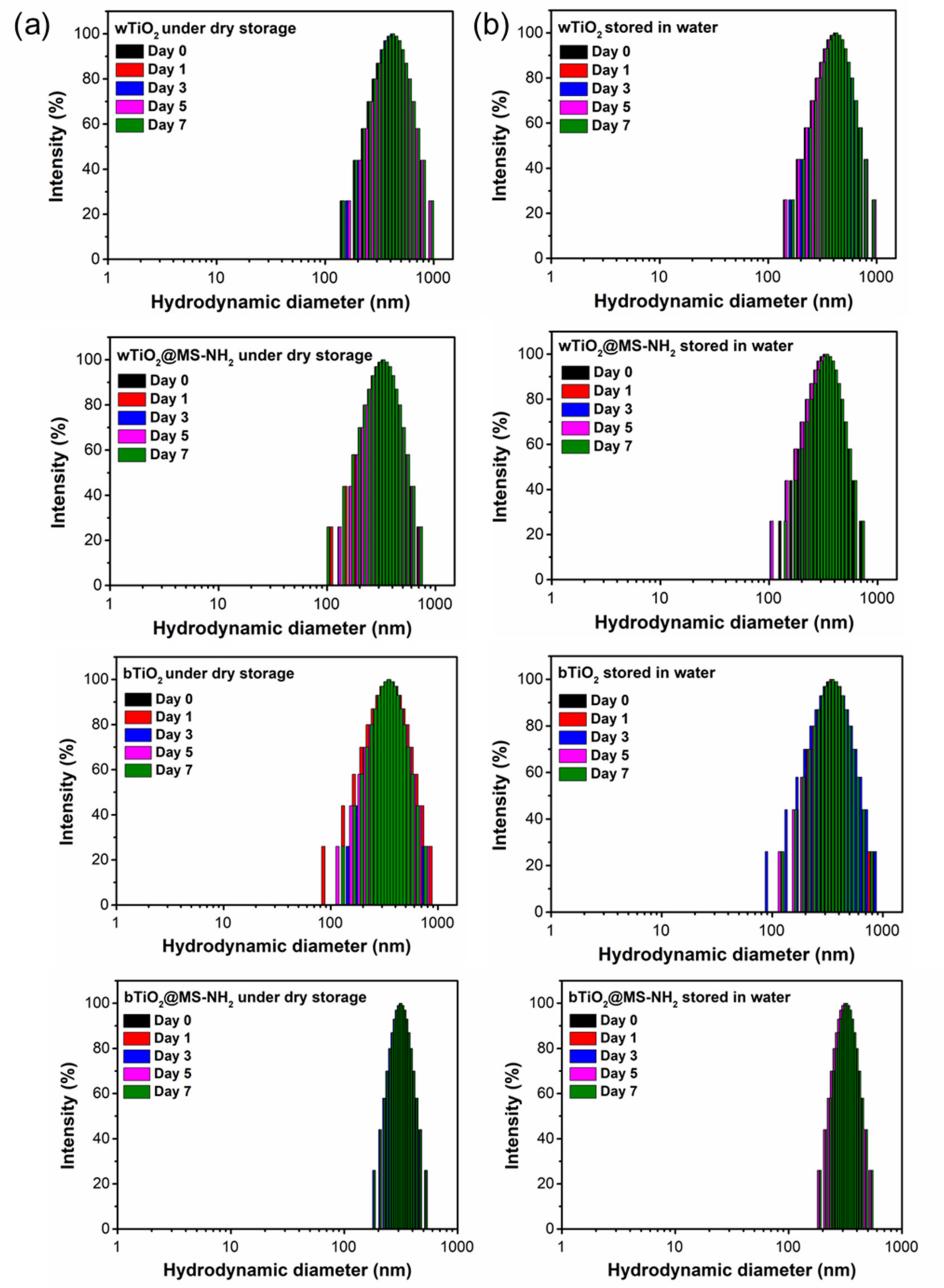

Figure S7. Hydrodynamic diameter distributions of nanoparticles (a) under dry storage and (b) stored in water.

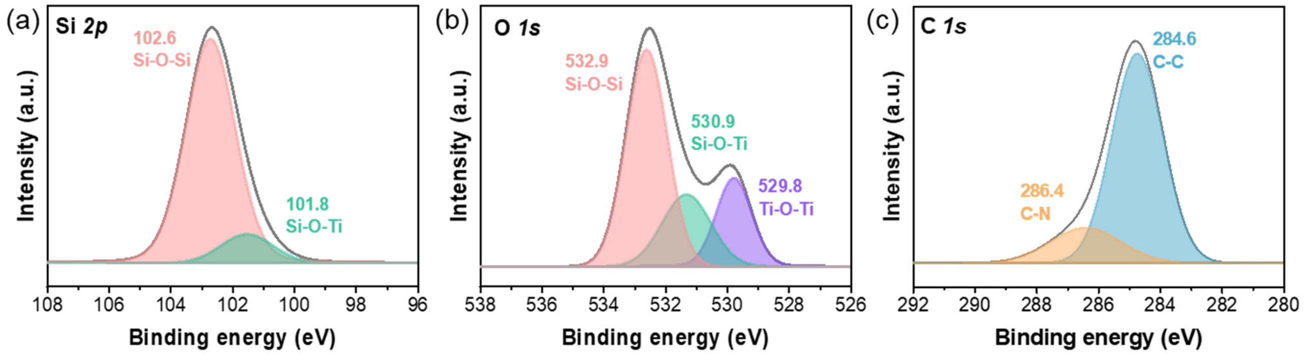

Figure S8. High-resolution XPS spectra for (a) Si *2p*, (b) O *1s*, and (c) C *1s* of $bTiO_2$@MS-NH_2 nanoparticles.

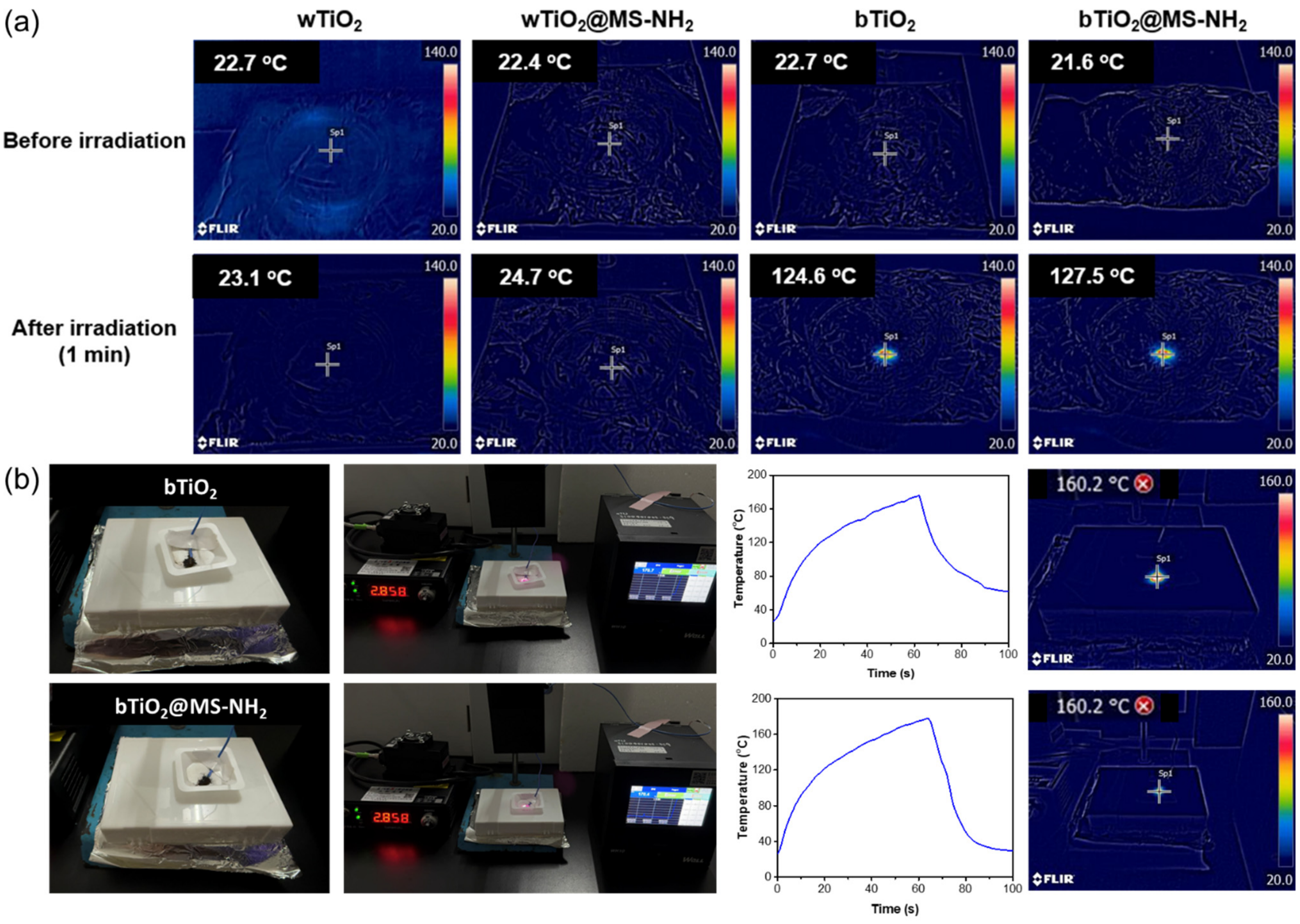

Figure S9. (a) Thermal images of nanoparticles (15 mg) under NIR irradiation (808 nm, 1 W cm^{-2}). (b) Thermal imaging and thermocouple readings of $bTiO_2$ and $bTiO_2$@MS-NH_2 nanoparticles (40 mg) under NIR irradiation (808 nm, 1 W cm^{-2}).

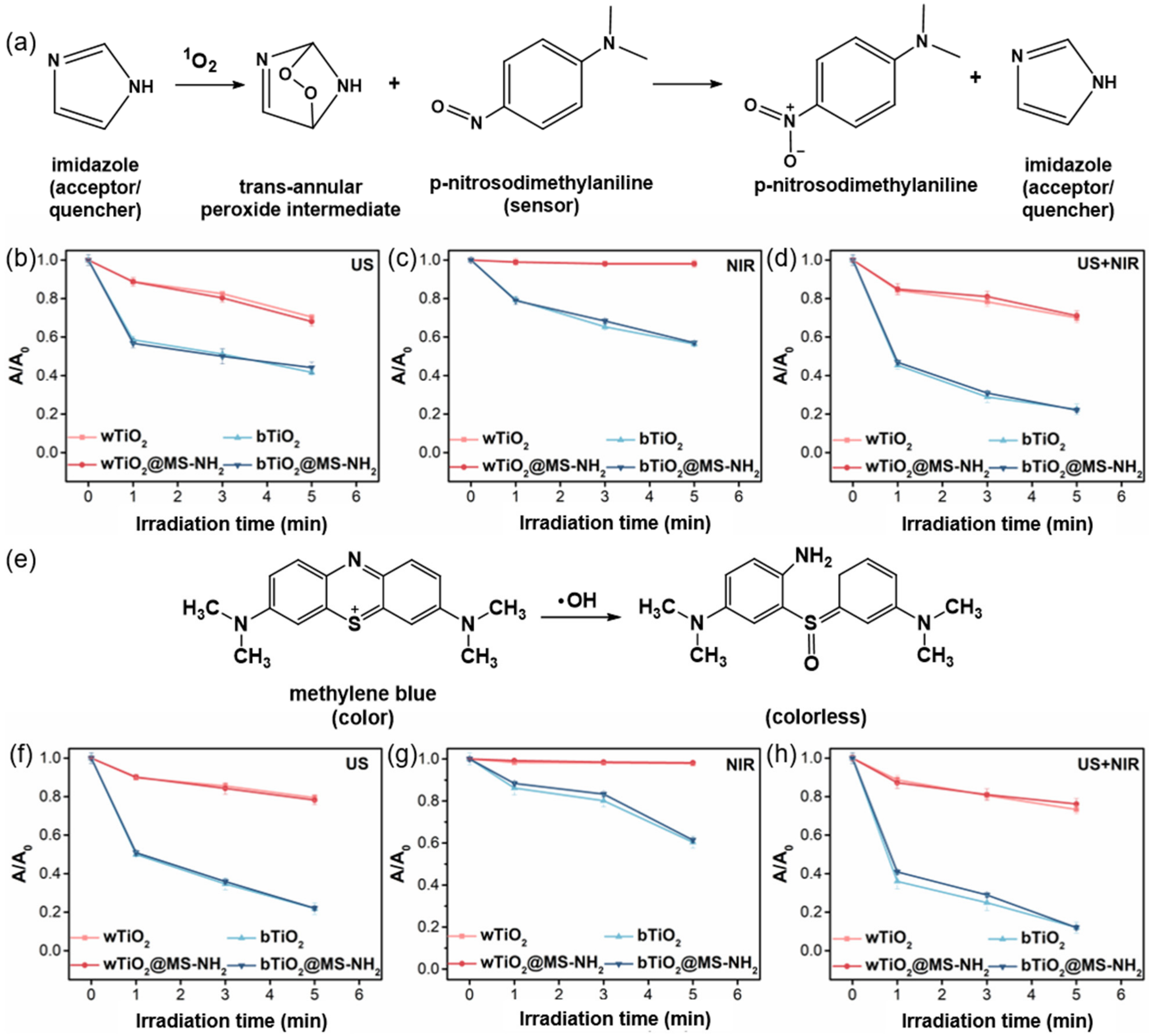

Figure S10. (a) The mechanism of RNO/imidazole assay. Time-dependent absorbance spectra of nanoparticles reacting with RNO/imidazole under (b) US irradiation (1 MHz, 1 W cm^{-2}, and 50% duty cycle), (c) NIR irradiation (808 nm, 1 W cm^{-2}), and (d) both NIR and US irradiations. (e) The mechanism of the methylene blue assay. Time-dependent absorbance spectra of nanoparticles reacting with MB under (f) US irradiation (1 MHz, 1 W cm^{-2}, and 50% duty cycle), (g) NIR irradiation (808 nm, 1 W cm^{-2}), and (h) both NIR and US irradiations.

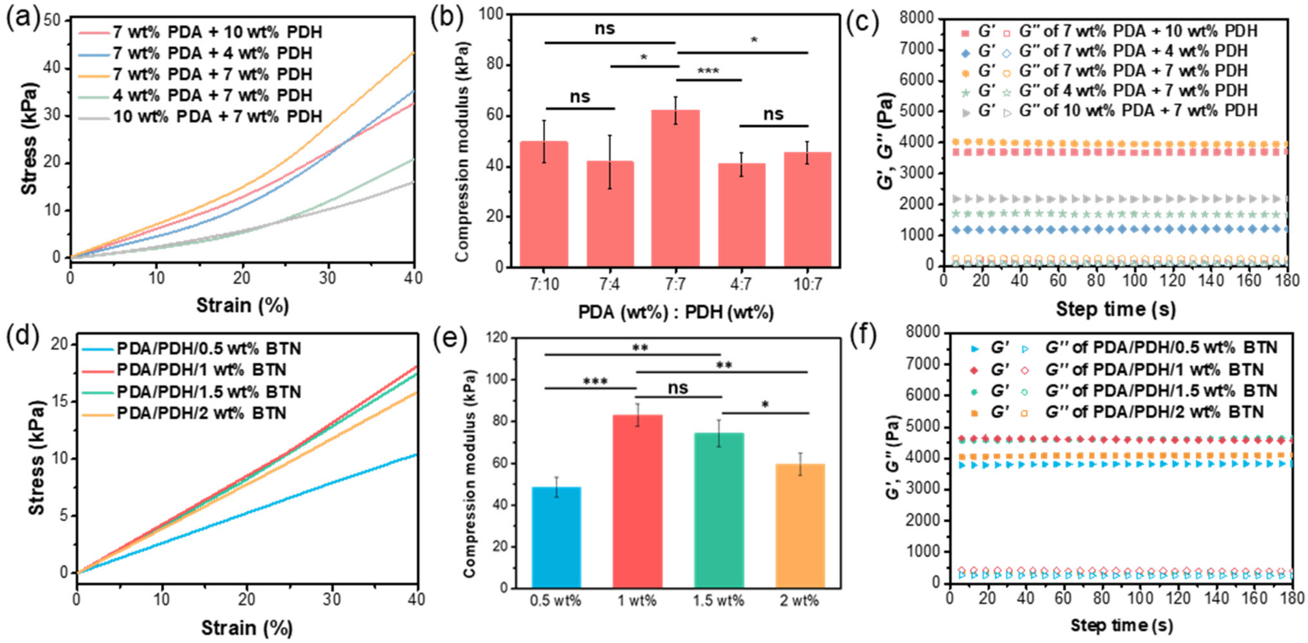

Figure S11. (a) Representative stress-strain curves in the compression tests, (b) compression modulus, and (c) time sweep of PDA/PDH hydrogels in various conditions. (d) Representative stress-strain curves in the compression tests, (e) compression modulus, and (f) time sweep of PDA/PDH/BTN hydrogel in various conditions (scale bar: 50 μm). Significance was set at *$p < 0.05$, **$p < 0.01$, and ***$p < 0.001$, and ns for no significant difference.

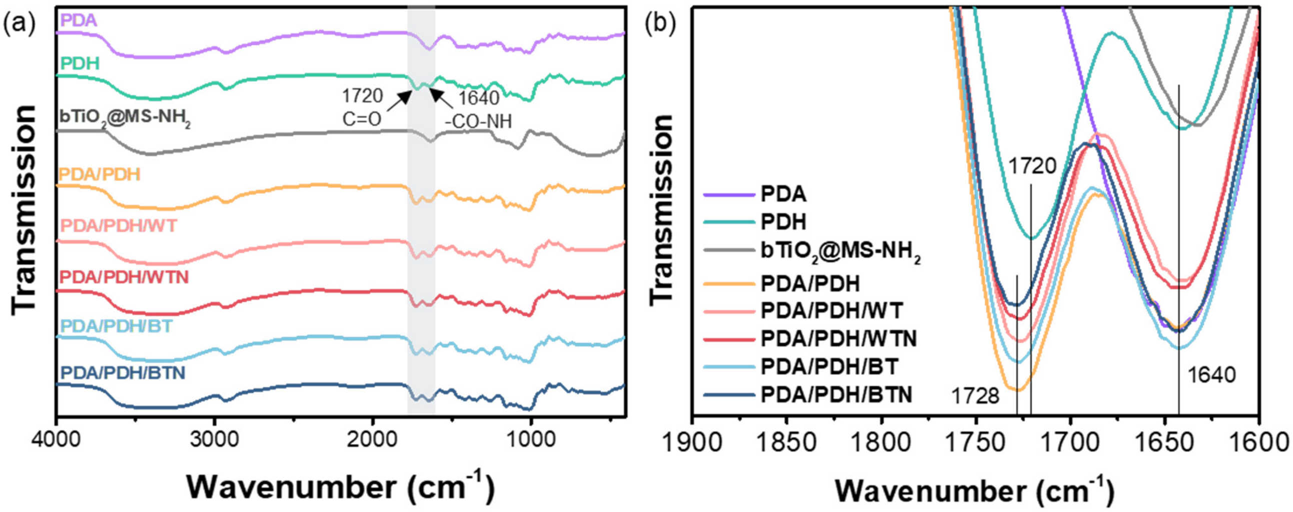

Figure S12. (a) FT-IR spectra and (b) overlaid FT-IR spectra (region 1900-1600 cm^{-1}) of PDA, PDH, $bTiO_2$@MS-NH_2, PDA/PDH, PDA/PDH/WT, PDA/PDH/WTN, PDA/PDH/BT, and PDA/PDH/BTN hydrogels.

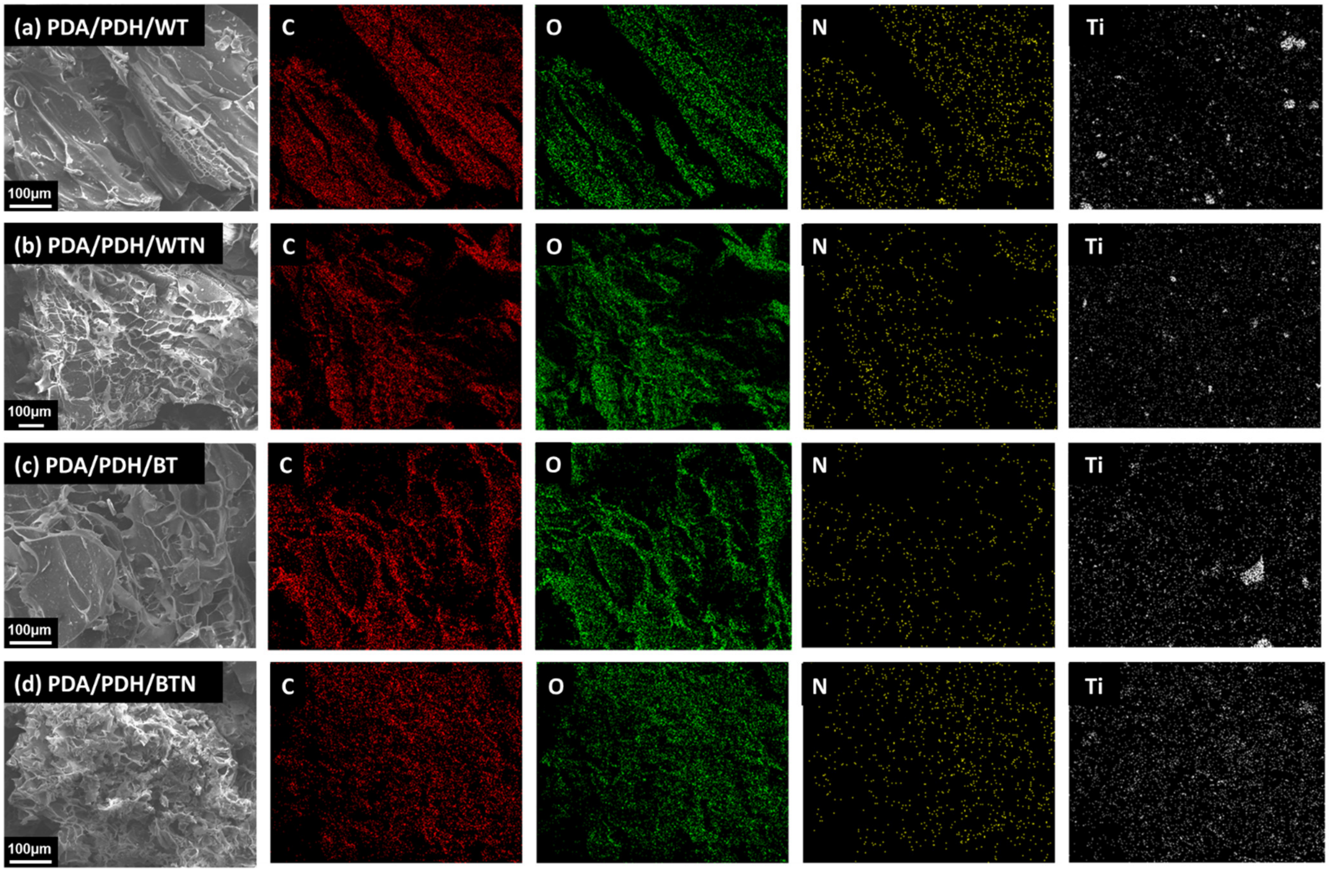

Figure S13. The SEM-EDS elemental mapping analysis of (a) PDA/PDH/WT, (b) PDA/PDH/WTN, (c) PDA/PDH/BT, and (d) PDA/PDH/BTN lyophilized hydrogels.

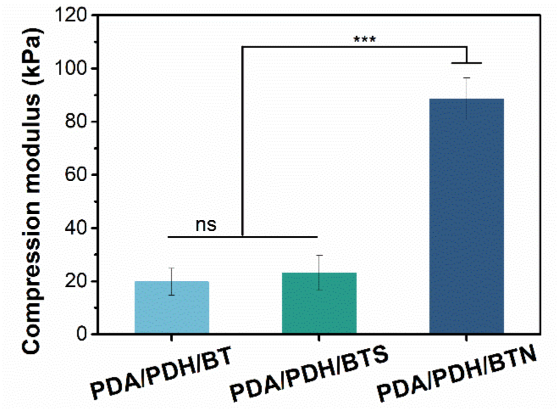

Figure S14. Compression modulus of PDA/PDH/BT, PDA/PDH/BTS (amine-free silica-coated $bTiO_2$, BTS), and PDA/PDH/BTN hydrogels.

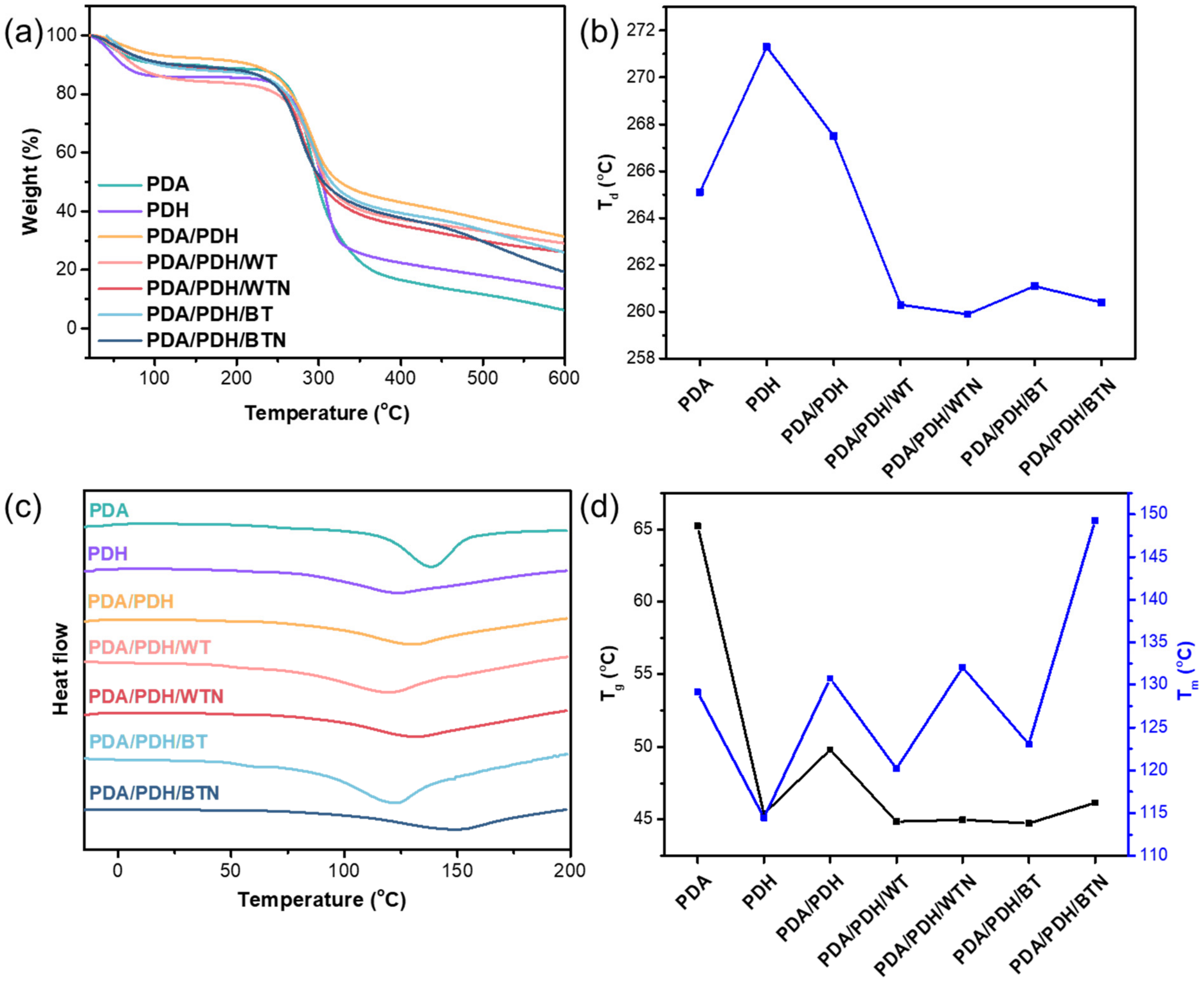

Figure S15. (a) TGA curves and (b) thermal degradation temperatures (T_d) of polymers and hydrogels from TGA analysis. (c) DSC curves and (d) glass transition temperatures (T_g) and melting temperatures (T_m) of polymers and hydrogels from DSC analysis.

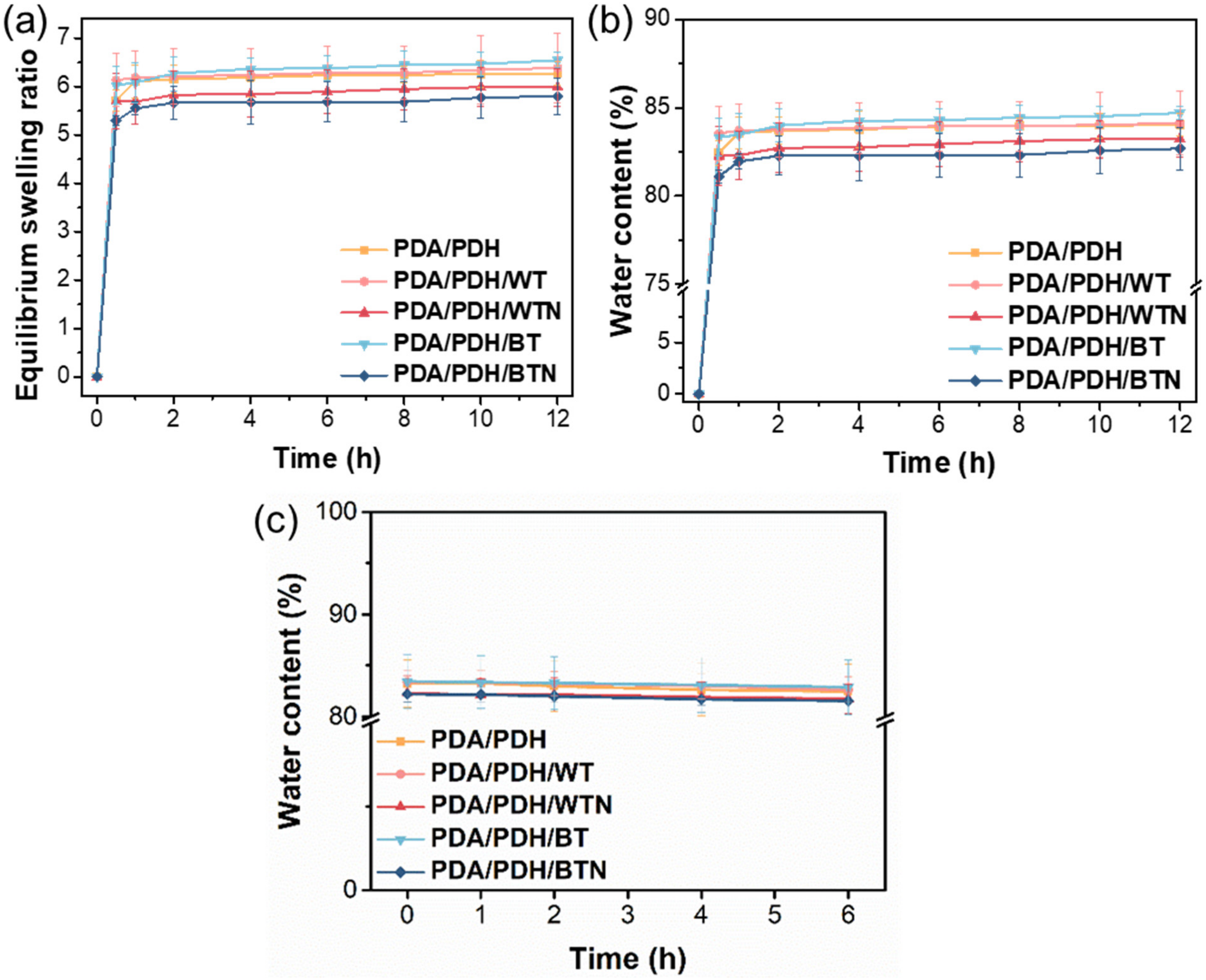

Figure S16. (a) Swelling ratios and (b) water contents of hydrogels. (c) The water contents of hydrogels after immersion were monitored for up to 6 hrs at 25 °C

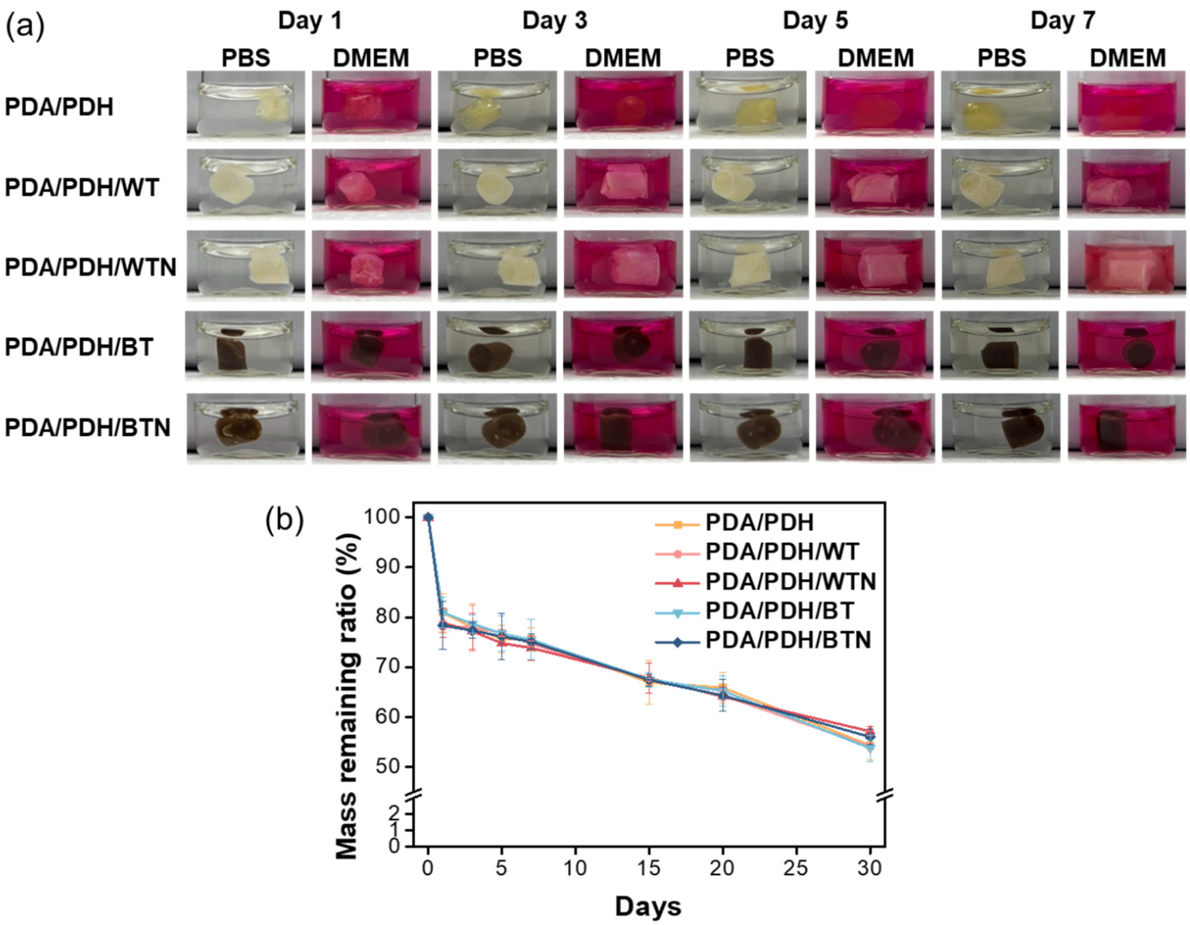

Figure S17. (a) Stability and (b) degradation tests of hydrogels.

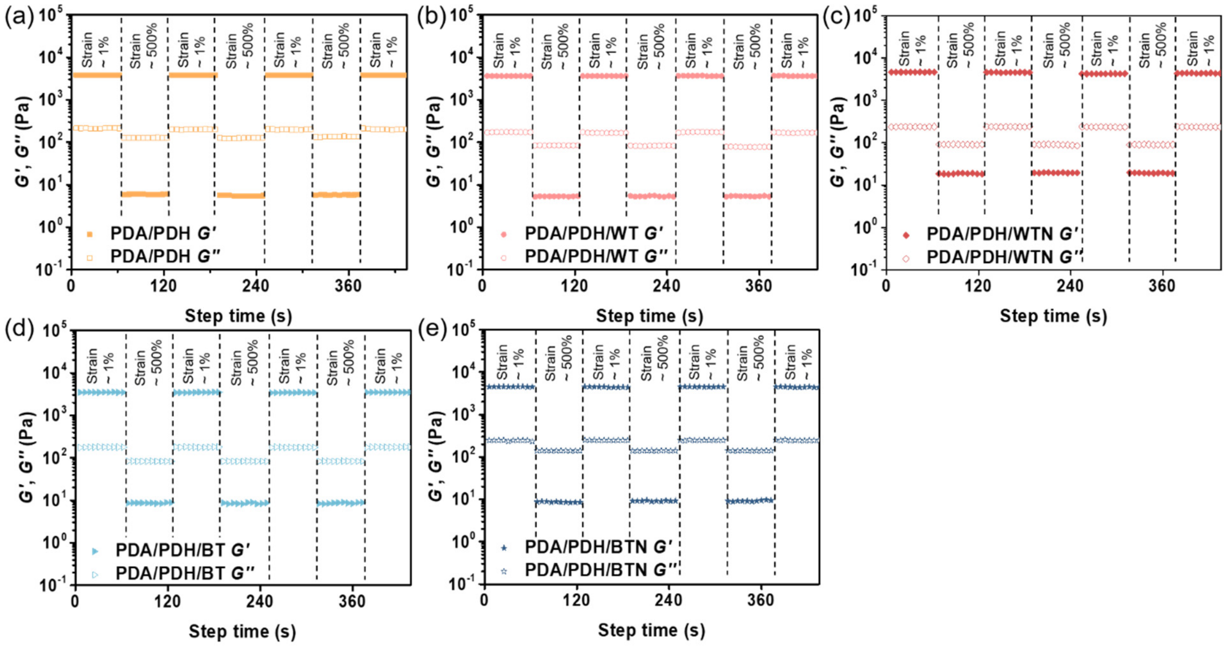

Figure S18. Cyclic strain time sweep of (a) PDA/PDH, (b) PDA/PDH/WT, (c) PDA/PDH/WTN, (d) PDA/PDH/BT, and (e) PDA/PDH/BTN hydrogels.

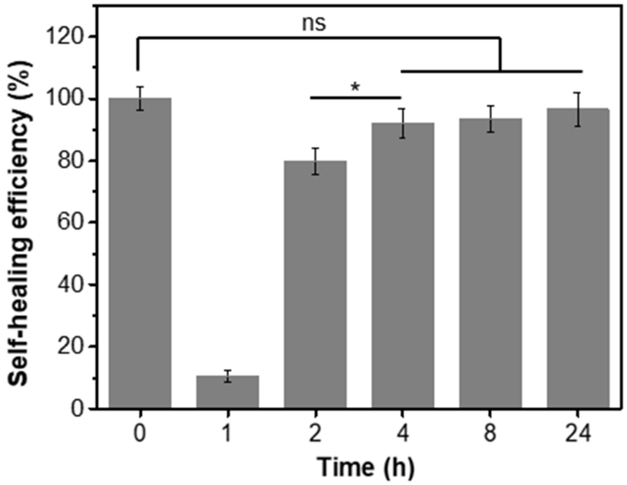

Figure S19. Self-healing efficiency tests of PDA/PDH/BTN hydrogel. Significant results were indicated: *$p < 0.05$ and ns for no significant difference.

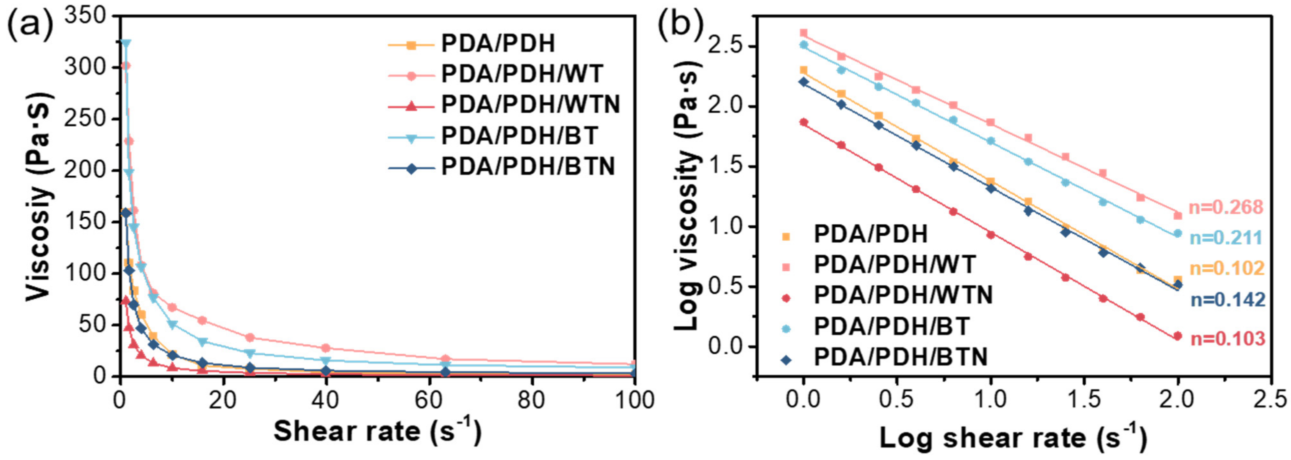

Figure S20. (a) The viscosity test of hydrogels, with the shear rate set between 0.1 and 100 s^{-1}. (b) Log viscosity- log shear rate curves of hydrogels.

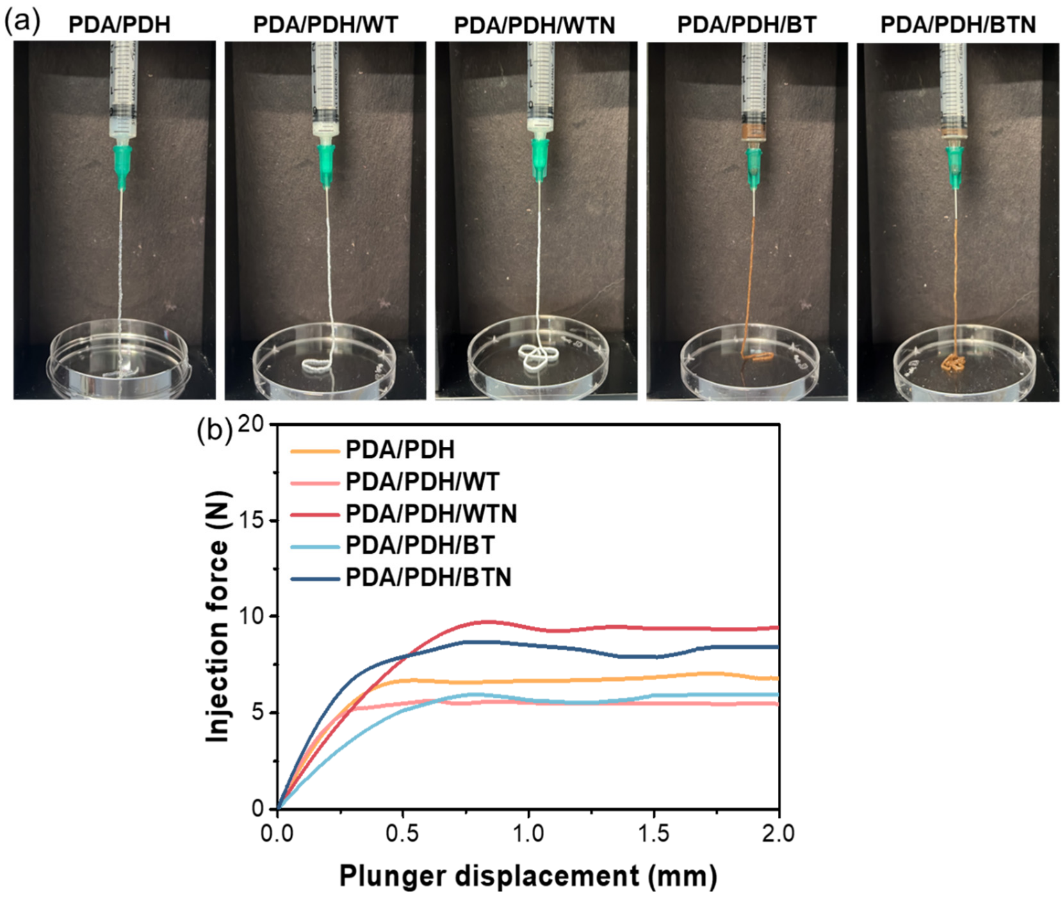

Figure S21. (a) Injection images and (b) injection profiles of hydrogels.

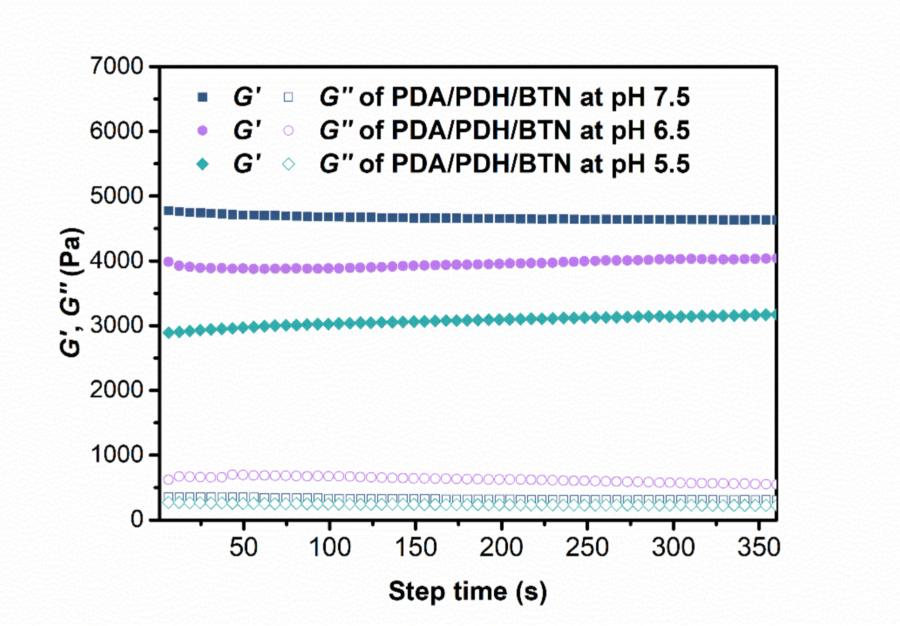

Figure S22. *G'* and *G"* changes of PDA/PDH/BTN hydrogel after immersion in different pH values of PBS solutions at 37 °C for 2 hrs.

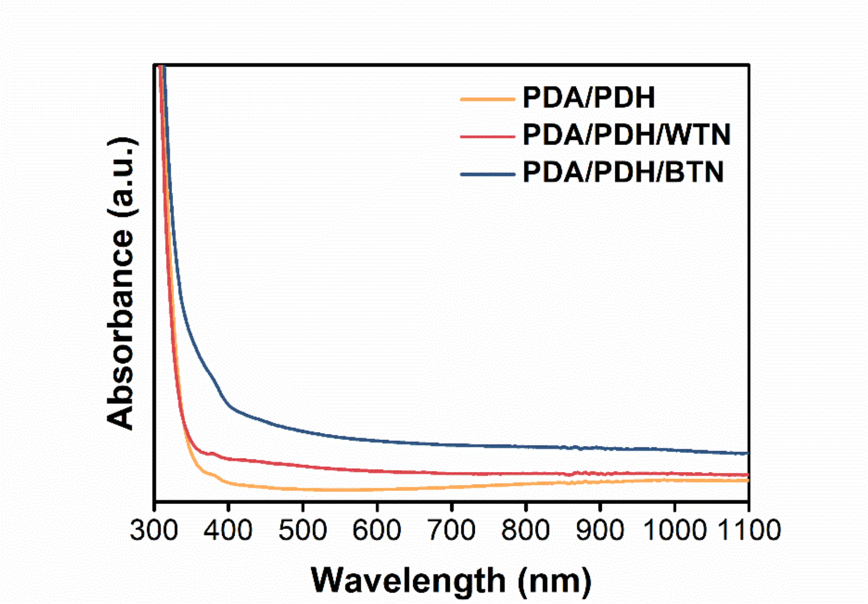

Figure S23. UV-Vis-NIR spectra of PDA/PDH, PDA/PDH/WTN, and PDA/PDH/BTN hydrogels.

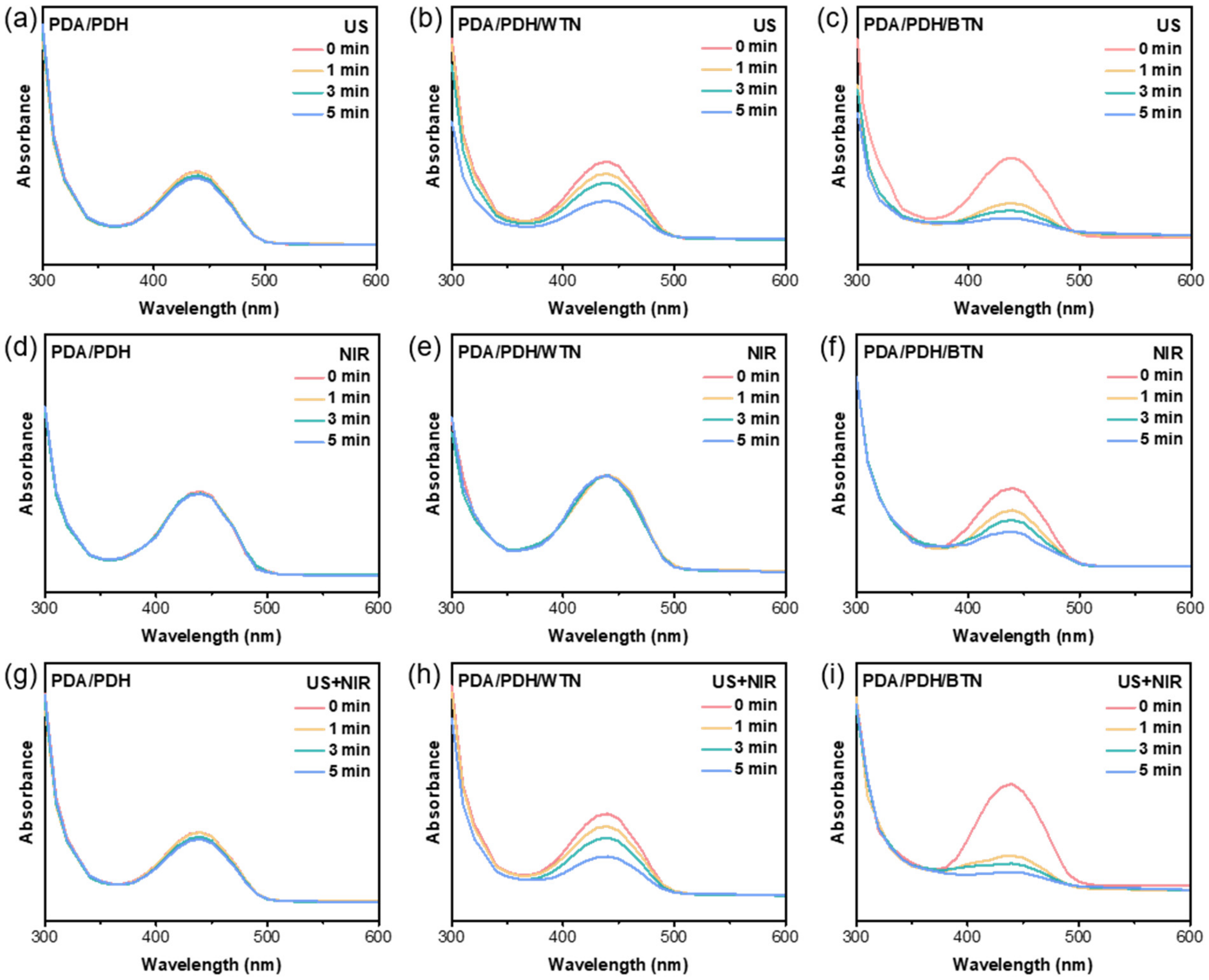

Figure S24. UV–vis spectra of PDA/PDH, PDA/PDH/WTN, and PDA/PDH/BTN hydrogels reacting with RNO/imidazole under different irradiation conditions: (a-c) US irradiation (1 MHz, 1 W cm^{-2}, 50% duty cycle), (d-f) NIR irradiation (808 nm, 1 W cm^{-2}), and (g-i) combined NIR and US irradiation.

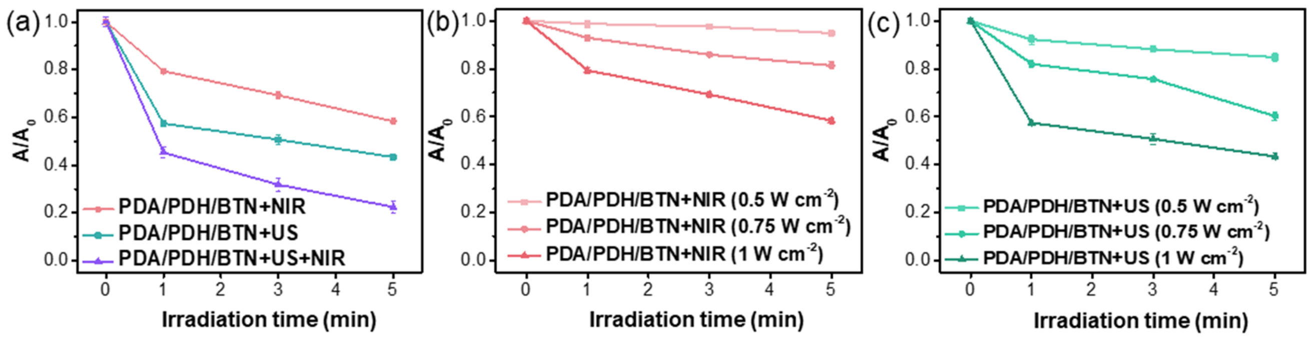

Figure S25. (a) Singlet oxygen generation of PDA/PDH/BTN hydrogel with different triggers. Singlet oxygen generation of PDA/PDH/BTN hydrogel under different power densities of (b) NIR and (c) US irradiation.

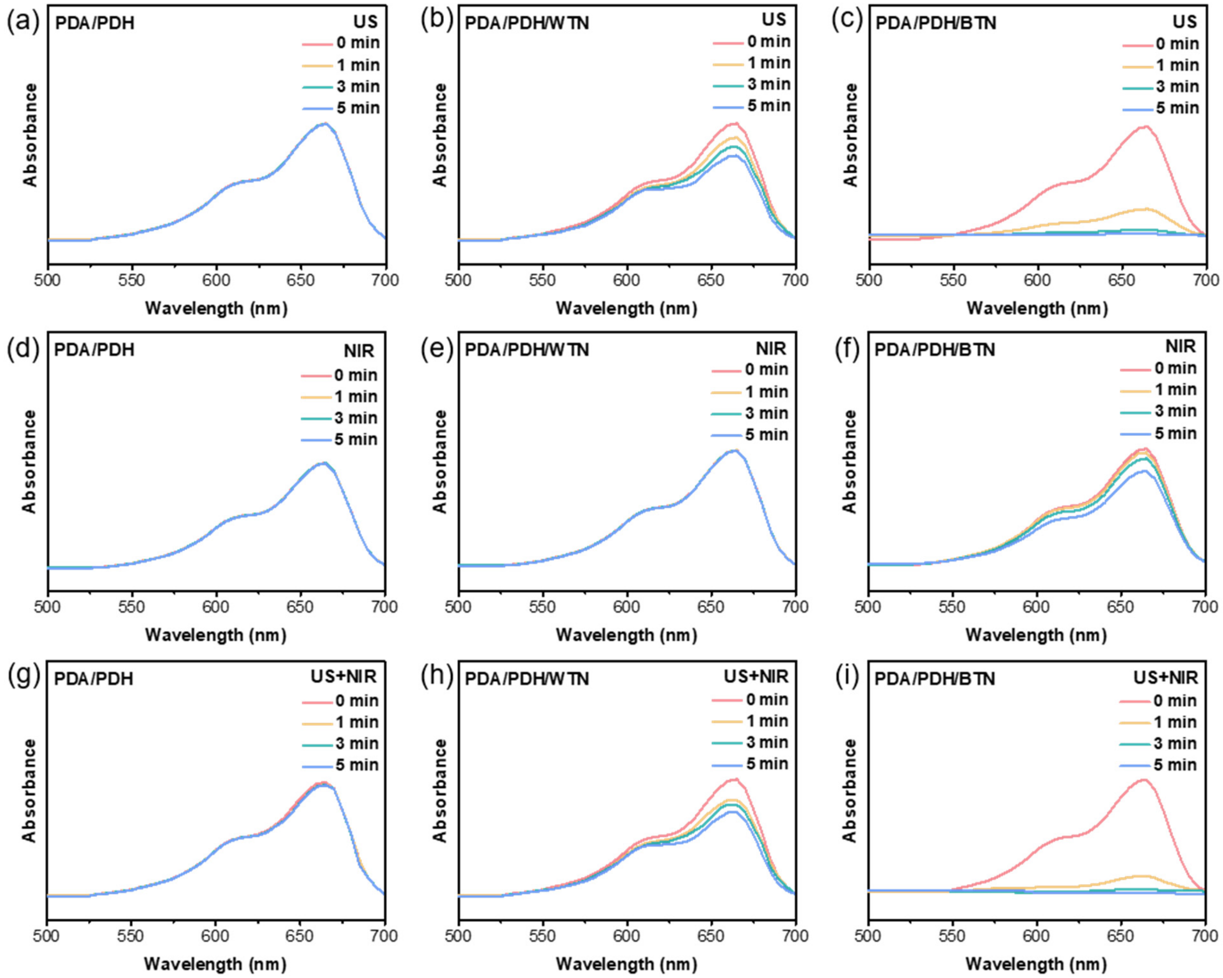

Figure S26. UV–vis spectra of PDA/PDH, PDA/PDH/WTN, and PDA/PDH/BTN hydrogels reacting with MB under different irradiation conditions: (a-c) US irradiation (1 MHz, 1 W cm^{-2}, 50% duty cycle), (d-f) NIR irradiation (808 nm, 1 W cm^{-2}), and (g-i) combined NIR and US irradiation.

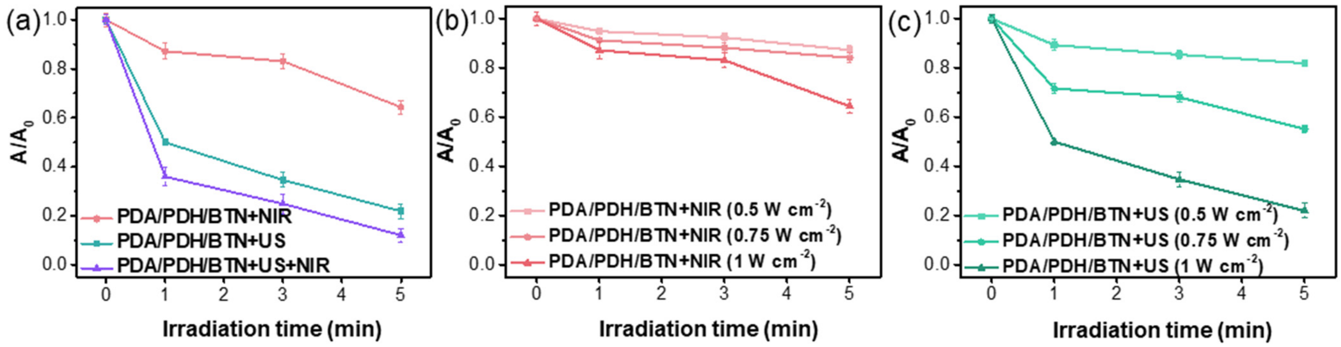

Figure S27. (a) Hydroxyl radical generation of PDA/PDH/BTN hydrogel with different triggers. Hydroxyl radical generation of PDA/PDH/BTN hydrogel under different power densities of (b) NIR and (c) US irradiation.

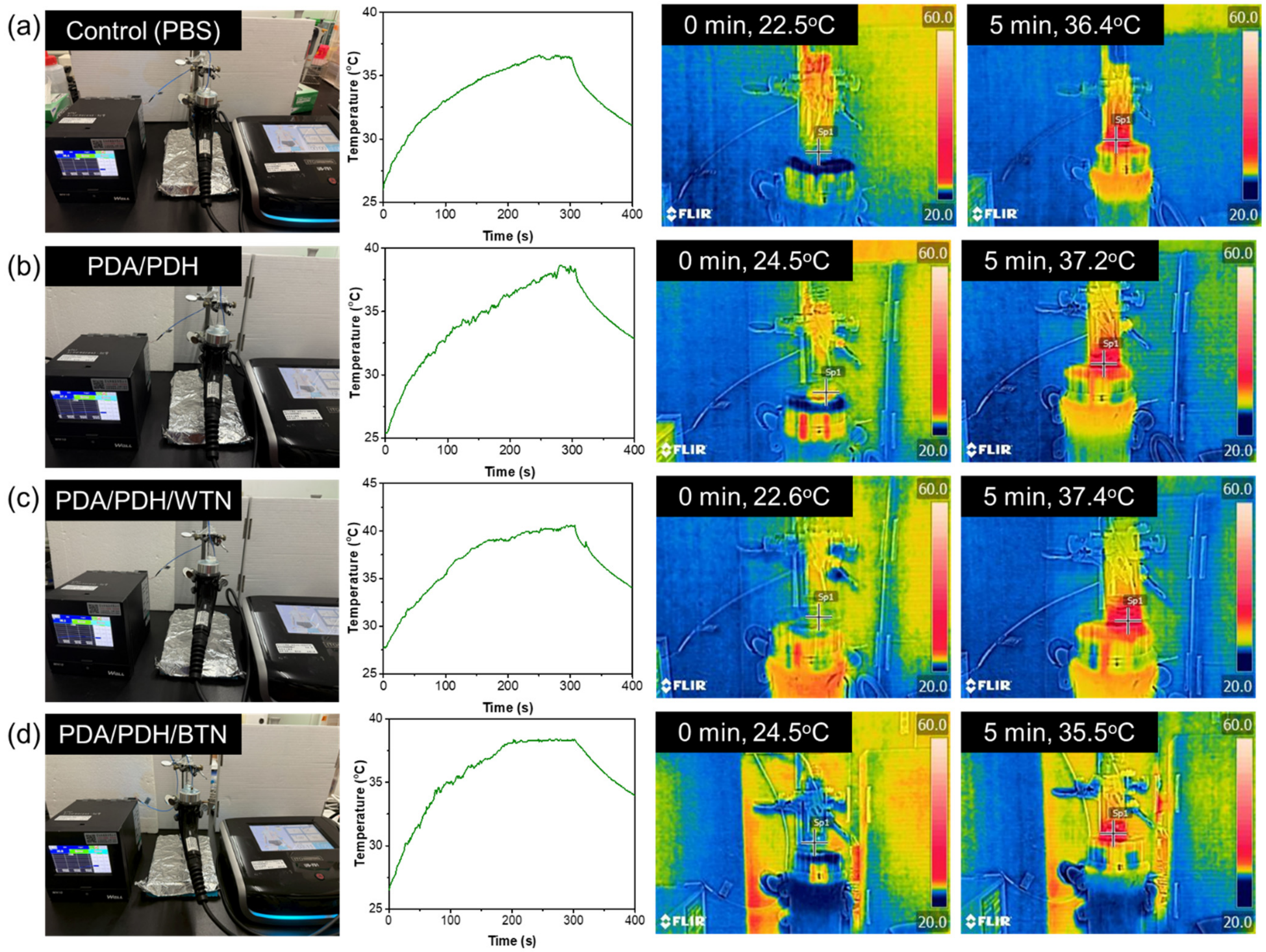

Figure S28. Thermal images of (a) PBS. Thermal images of (b) PDA/PDH, (c) PDA/PDH/WTN, and (d) PDA/PDH/BTN hydrogels immersed in PBS solutions under US irradiation (1 MHz, 1 W cm^{-2}, 50% duty cycle).

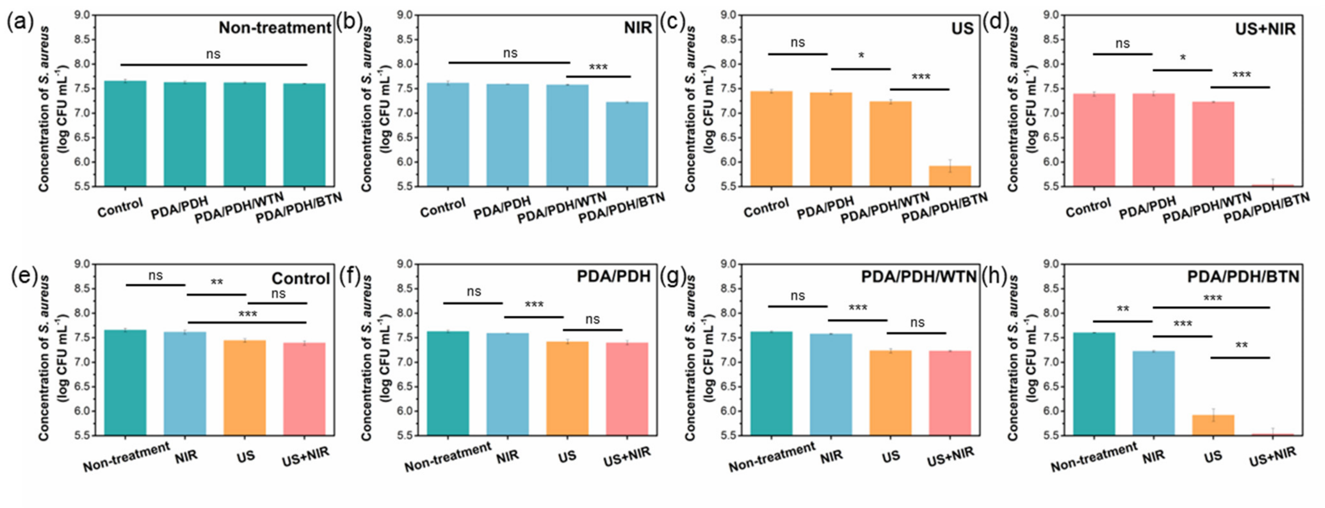

Figure S29. Quantitative analysis of *S. aureus* via spread plate method under (a) non-treatment, (b) NIR, (c) US, and (d) US+NIR treatment of hydrogels. Quantitative analysis of *S. aureus* under different treatments of (e) bacterial suspension, (f) PDA/PDH, (g) PDA/PDH/WTN, and (h) PDA/PDH/BTN hydrogels. Significant results were indicated: *p< 0.05, **p< 0.01, ***p< 0.001, and ns for no significant difference.

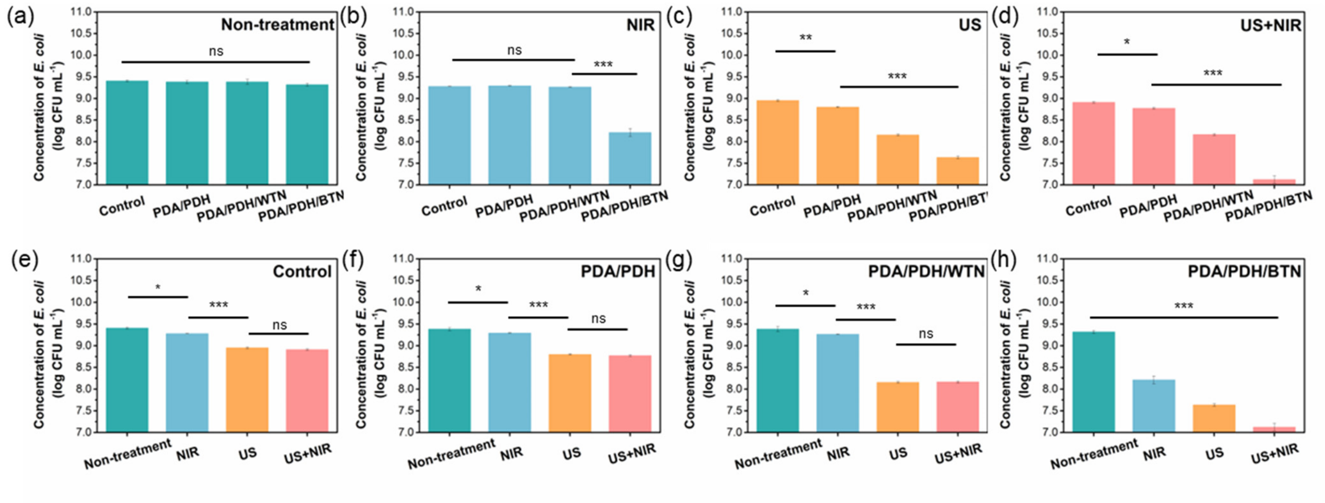

Figure S30. Quantitative analysis of *E. coli* via spread plate method under (a) non-treatment, (b) NIR, (c) US, and (d) US+NIR treatment of hydrogels. Quantitative analysis of *E. coli* under different treatments of (e) bacterial suspension, (f) PDA/PDH, (g) PDA/PDH/WTN, and (h) PDA/PDH/BTN hydrogels. Significant results were indicated: *$p< 0.05$, **$p< 0.01$, ***$p< 0.001$, and ns for no significant difference.

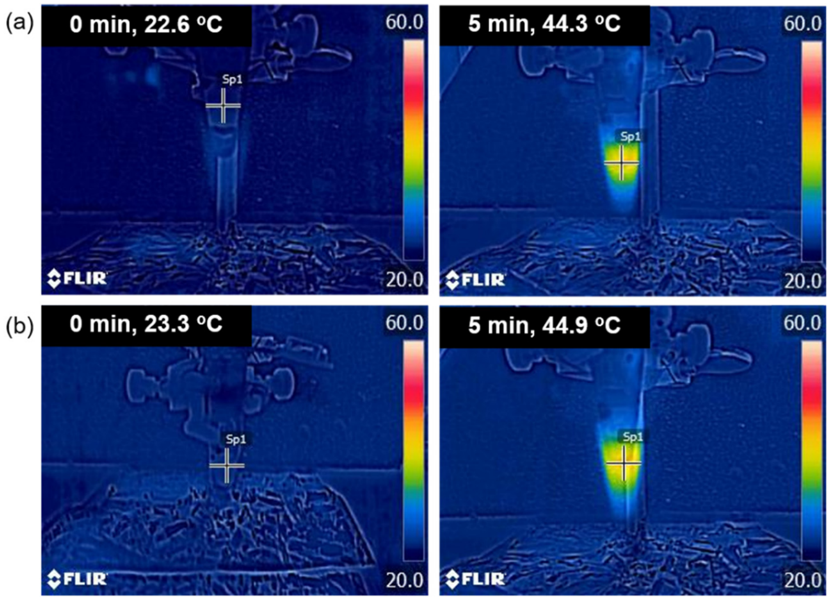

Figure S31. Thermal images of PDA/PDH/BTN hydrogel immersed in (a) *S. aureus* and (b) *E. coli* suspension under NIR irradiation (808 nm, 1 W cm^{-2}).

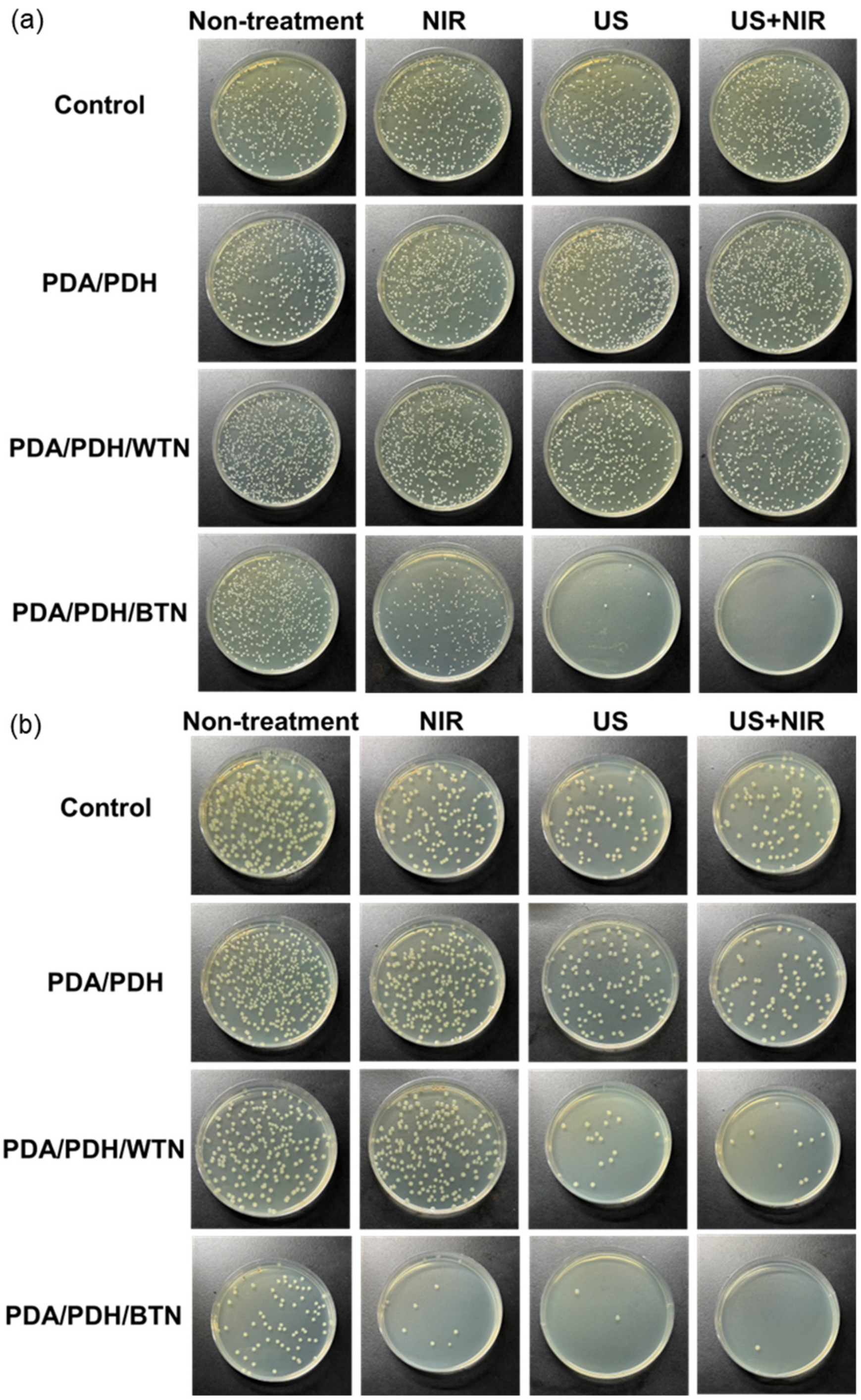

Figure S32. *In vitro* antibacterial efficacy of hydrogels. The images of (a) *S. aureus* and (b) *E. coli* colonies on agar plates after different treatments. US: treat by ultrasound (1 MHz, 1 W cm^{-2}, 50% duty cycle) for 5 mins; NIR: treat by 808 nm laser (1 W cm^{-2}) for 5 mins. The bacterial samples were diluted using sterile PBS: *S. aureus* at 10^4-fold and *E. coli* at 10^6-fold.

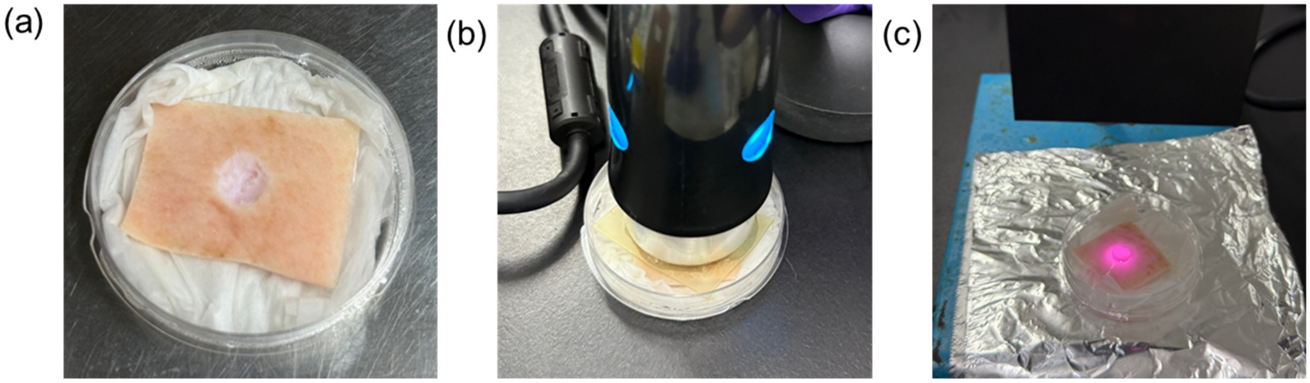

Figure S33. (a) The image of the wound model on porcine skin. The image of PDA/PDH/BTN hydrogel under (b) US (1 MHz, 1 W cm^{-2}, and 50% duty cycle) and (c) NIR (808 nm, 1 W cm^{-2}) treatment.

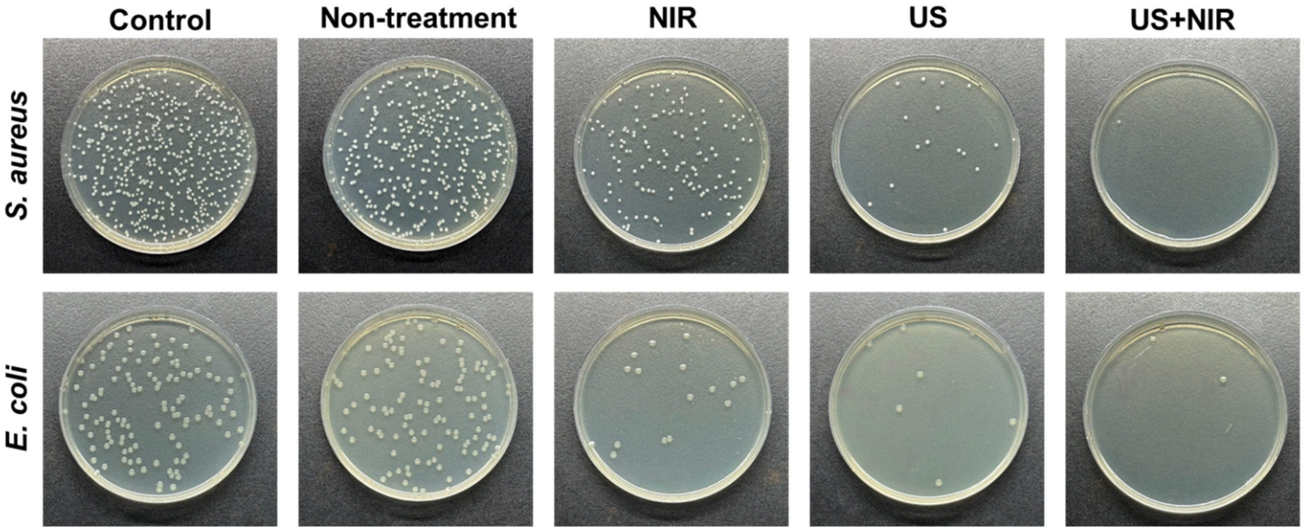

Figure S34. *Ex vivo* antibacterial efficacy of PDA/PDH/BTN hydrogel. The images of *S. aureus* and *E. coli* colonies on agar plates after different treatments. US: treat by ultrasound (1 MHz, 1 W cm^{-2}, 50% duty cycle) for 5 mins; NIR: treat by 808 nm laser (1 W cm^{-2}) for 5 mins. The bacterial samples were diluted using sterile PBS: *S. aureus* at 10^4-fold and *E. coli* at 10^5-fold.

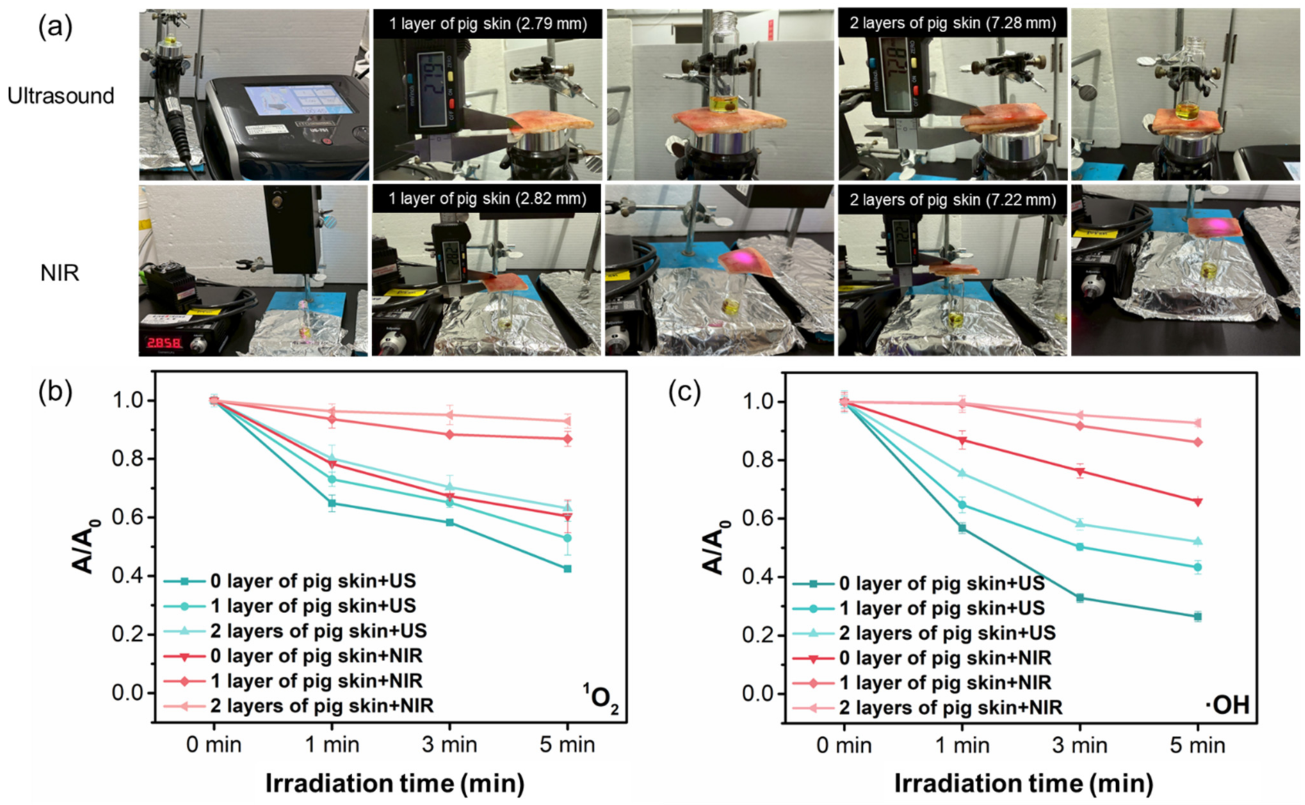

Figure S35. ROS generation under US and NIR irradiation through porcine skin (0, 1, and 2 layers): (a) schematic illustration of the experimental setup, (b) singlet oxygen ($^1O_2$) generation, and (c) hydroxyl radical (•OH) generation.

Table S1. Hydrodynamic diameter (D_h) and polydispersity index (PDI) of nanoparticles after dry storage and storage in water, measured in water.

	$wTiO_2$				$wTiO_2$@MS-NH_2			
	Dry storage		Storage in water		Dry storage		Storage in water	
	D_h	PDI	D_h	PDI	D_h	PDI	D_h	PDI
Day 0	417.2	0.183	417.6	0.194	331.5	0.138	328.5	0.141
Day 1	414.8	0.202	418.3	0.198	333.0	0.142	331.4	0.153
Day 3	413.1	0.198	414.0	0.202	329.5	0.151	330.5	0.146
Day 5	419.4	0.182	417.0	0.201	330.0	0.140	331.2	0.149
Day 7	416.7	0.193	420.2	0.182	329.1	0.142	332.5	0.159
	$bTiO_2$				$bTiO_2$@MS-NH_2			
	Dry storage		Storage in water		Dry storage		Storage in water	
	D_h	PDI	D_h	PDI	D_h	PDI	D_h	PDI
Day 0	347.9	0.189	348.2	0.183	313.1	0.128	314.0	0.121
Day 1	349.9	0.193	347.6	0.194	311.6	0.123	315.9	0.126
Day 3	345.9	0.188	353.6	0.201	310.2	0.119	316.4	0.120
Day 5	348.9	0.191	349.9	0.179	316.8	0.121	310.9	0.117
Day 7	347.4	0.188	348.4	0.187	312.2	0.124	313.1	0.124

Table S2. The average pore size and porosity of hydrogels analyzed by micro-CT.

	Average pore size (μm)	Closed porosity (%)	Open porosity (%)	Total porosity (%)
PDA/PDH	117.9	0.013	77.2	77.2
PDA/PDH/WT	129.0	0.003	81.1	81.1
PDA/PDH/WTN	106.0	0.008	78.3	78.2
PDA/PDH/BT	135.2	0.014	80.3	80.3
PDA/PDH/BTN	92.0	0.005	76.0	76.0

Table S3. The intrusion volume, pore size, and porosity of hydrogels were analyzed by MIP.

	Total intrusion volume (mL g^{-1})	Total pore area (m^2 g^{-1})	Median pore diameter (volume) (μm)	Porosity (%)
PDA/PDH	3.9	0.8	34.5	78.7
PDA/PDH/WT	3.9	0.7	44.2	81.4
PDA/PDH/WTN	3.9	0.8	32.9	94.5
PDA/PDH/BT	3.3	0.9	46.2	97.5
PDA/PDH/BTN	4.0	0.8	28.1	95.0

Table S4. Rheological properties of hydrogels.

	***G'* (Pa)**	***G''* (Pa)**	**Flow point (%)**
PDA/PDH	3882.7 ± 116.5	216.2 ± 4.6	58.0 ± 4.6
PDA/PDH/WT	3226.1 ± 96.8	193.6 ± 5.8	59.1 ± 5.2
PDA/PDH/WTN	4680.6 ± 118.4	245.7 ± 4.3	102.8 ± 5.3
PDA/PDH/BT	3293.8 ± 82.4	173.6 ± 5.2	69.7 ± 4.9
PDA/PDH/BTN	4510.4 ± 90.2	255.0 ± 6.6	92.9 ± 4.8
